# Chaga mushroom: a super-fungus with countless facets and untapped potential

**DOI:** 10.3389/fphar.2023.1273786

**Published:** 2023-12-05

**Authors:** Eric Fordjour, Charles F. Manful, Rabia Javed, Lakshman W. Galagedara, Chad W. Cuss, Mumtaz Cheema, Raymond Thomas

**Affiliations:** ^1^ Biotron Experimental Climate Change Research Centre, Department of Biology, University of Western Ontario, London, ON, Canada; ^2^ School of Science and the Environment, Grenfell Campus, Memorial University of Newfoundland, Corner Brook, NL, Canada

**Keywords:** Chaga, mycochemistry, ethnomycology, pharmacology, agricultural uses, traditional uses

## Abstract

*Inonotus obliquus* (Chaga mushroom) is an inexpensive fungus with a broad range of traditional and medicinal applications. These applications include therapy for breast, cervix, and skin cancers, as well as treating diabetes. However, its benefits are virtually untapped due to a limited understanding of its mycochemical composition and bioactivities. In this article, we explore the ethnobotany, mycochemistry, pharmacology, traditional therapeutic, cosmetic, and prospective agricultural uses. The review establishes that several secondary metabolites, such as steroids, terpenoids, and other compounds exist in chaga. Findings on its bioactivity have demonstrated its ability as an antioxidant, anti-inflammatory, antiviral, and antitumor agent. The study also demonstrates that Chaga powder has a long history of traditional use for medicinal purposes, pipe smoking rituals, and mystical future forecasts. The study further reveals that the applications of Chaga powder can be extended to industries such as pharmaceuticals, food, cosmetics, and agriculture. However numerous publications focused on the pharmaceutical benefits of Chaga with few publications on other applications. Overall, chaga is a promising natural resource with a wide range of potential applications and therefore the diverse array of therapeutic compounds makes it an attractive candidate for various applications such as plant biofertilizers and active ingredients in cosmetics and pharmaceutical products. Thus, further exploration of Chaga’s potential benefits in agriculture and other industries could lead to exciting new developments and innovations.

## 1 Introduction

*Inonotus obliquus* (Chaga mushroom) is a sterile tree-destroying fungus that parasitizes the trunks of living birches. It belongs to the family Hymenochaetaceae and thrives in humid parts of Europe, Asia, and North America (Razumov et al., 2020; Abu-Reidah et al., 2021). The genus *Inonotus* includes about 100 species in a broad sense all of which have been identified as plant pathogens (Zhou and Wang, 2015). The distinction reflects changes in different groupings of the species over time as taxonomists have gained a better understanding of the relationships between different fungal species (May et al., 2019). The species *obliquus* is well-known for its medicinal and pharmacological properties (Balandaykin and Zmitrovich, 2015).

The preparation methods for indigenous herbal and non-medicinal uses of Chaga vary according to tribes and cultures. In China, Chaga mushrooms are processed for multiple applications by heating and crushing in water (Hwang et al., 2019). The same extraction method is applied in South Korea for similar purposes (Hong et al., 2015). In some tribes, Chaga mushrooms are used to boost the immune system and treat various diseases including diabetes and tumors (Abu-Reidah et al., 2021). In folk medicine, chaga is commonly consumed as a dietary mushroom as studies show it contains high levels of antioxidants, proteins, minerals, fiber, and vitamins. Apart from diet, it also plays a role as a functional food ingredient with several reported health-promoting activities including anti-cancer, anti-inflammatory, antioxidant, and antithrombotic (Lindequist et al., 2005; Rhee et al., 2008; Abu-Reidah et al., 2021). Other studies report the mushrooms’ general impact on the immune system and its potential in improving insulin resistance in type 2 diabetes (Géry et al., 2018; Abu-Reidah et al., 2021).

A comprehensive literature review (1900–2022) was conducted to assess the biological activities, mycochemical constituents, medicinal, and some nutritional potential of Chaga mushroom to supplement prior attempts focused on medicinal value (Milyuhina et al., 2022; Teplyakova et al., 2022). Globally, Chaga mushrooms are known for their therapeutic benefits, and novel bioactive metabolites in pharmaceutical, chemical, and cosmetic products (Peng and Shahidi, 2022). The application of Chaga is not limited to the medical field alone. The antioxidant properties of the fungus have demonstrated potential in animal production (Li et al., 2022). The addition of the mushroom to a nutrient matrix for livestock has been shown to increase the bioavailability of vitamin A in the digestive tract of livestock (Li et al., 2022). With these demonstrated benefits, the mushroom can be harnessed for use in other fields. Therefore, exploring the use of chaga in production of fertilizer can reduce environmental contamination that pose threat to both animal and plant and the overreliance on chemical fertilizer. This article therefore presents a summary of the biological functions of the Chaga mushroom linked to medical and agricultural applications.

## 2 Methodology

A systematic and comprehensive search strategy was employed to source all relevant materials. This review covers material published between the years 1900 and Nov. 2022, including periodicals, books and/or chapters, essays, research articles, review papers, theses, and minutes from meetings. Only information written in the English language was located using web search engines such as Google, Google Scholar, Pub Med, Google Books, Semantic Scholar, Science Direct, Scopus, Worldwide Science, Web of Science, JSTOR, and ResearchGate. Approximately 90 scientific documents were used for this review. The word “Chaga” was also cross-referenced with related phrases, such as medicinal mushrooms, phytochemistry, biological qualities, and nutritional properties using Boolean operators such as ‘AND’, ‘OR’, ‘NOT’, or ‘AND NOT’.

## 3 Ethnomycology description

### 3.1 Scientific classification and common names of *Inonotus obliquus*

**FIGURE 1:**
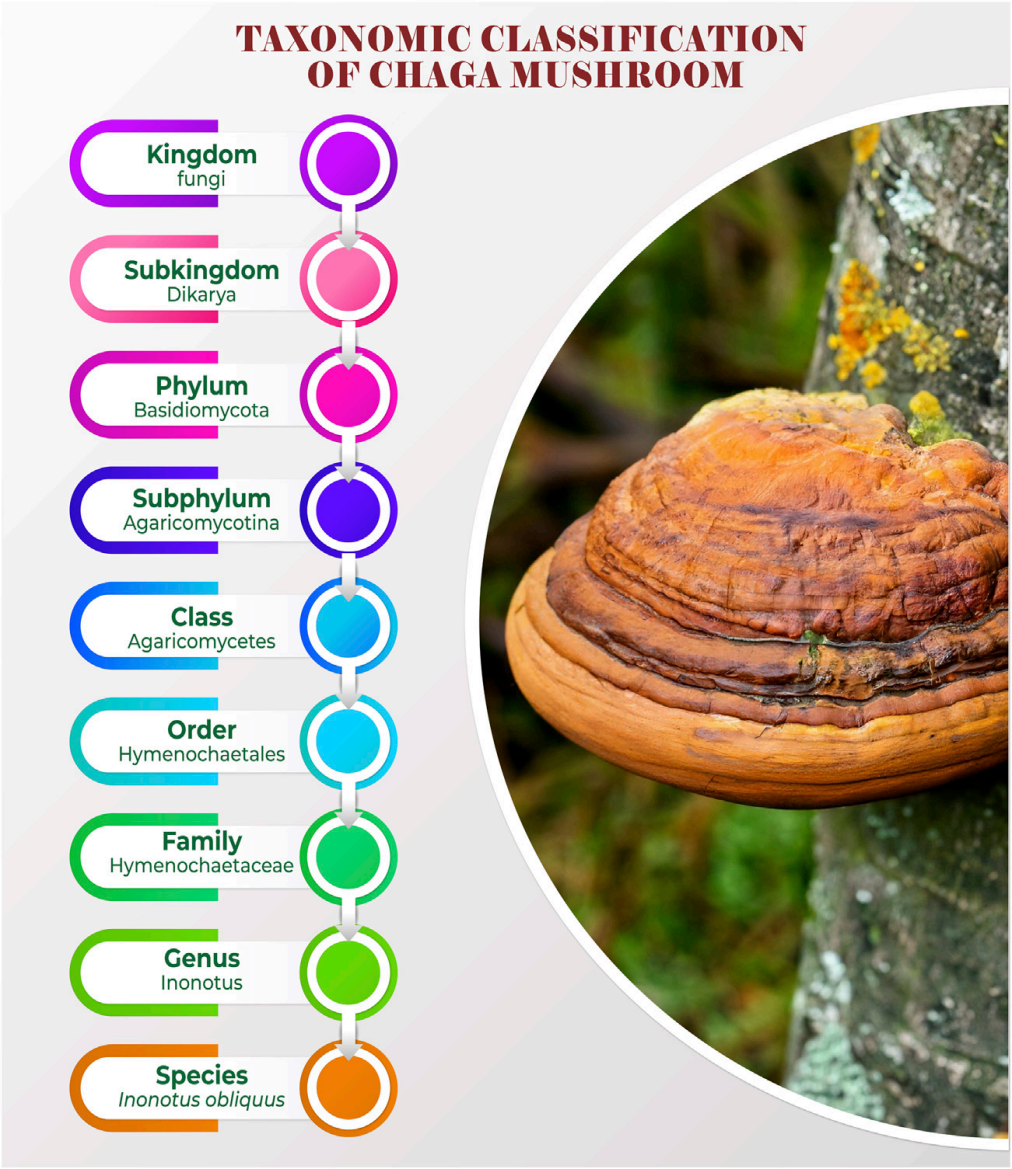
Taxonomic classification of Chaga mushroom.

According to Federhen (2012) scientific nomenclature “INOS” denotes fibre, “NOTON” denotes back, and “OBLIQUE” denotes unevenness of the sides (Figure 1) (Federhen, 2021).

Common names include Chaga, “Tschaga, Tschagapilz” (Russian), black birch touchwood, malalon mushroom, sterile conk trunk (North America/Europe), “Kreftjuilce” (Cancer polypore) (Norway), “Tikkatee” (Finland) and “Kabanoanatake” (Japan) (Zabel, 1976; Lee et al., 2008; Zhong et al., 2009; Beltrame et al., 2021). Chaga has several common names: in Russia, the fungus is called Chaga, which is derived from “Komi-Permyak,” the language of the Kama Basin, west of the Ural Mountains, but in England and Canada, Chaga is referred to as the birch’s sterile conk trunk (Zabel, 1976; Lee et al., 2008; Zhong et al., 2009; Beltrame et al., 2021). Owing to its therapeutic uses in modern medicine, Chaga is also known as the “Mushroom of Immortality”, in Japan, it is known as the “Diamond of the Forest” and the “King of Plants” in China (Nakajima et al., 2007; Zyryanova et al., 2010).

### 3.2 Distribution and ecology

**FIGURE 2:**
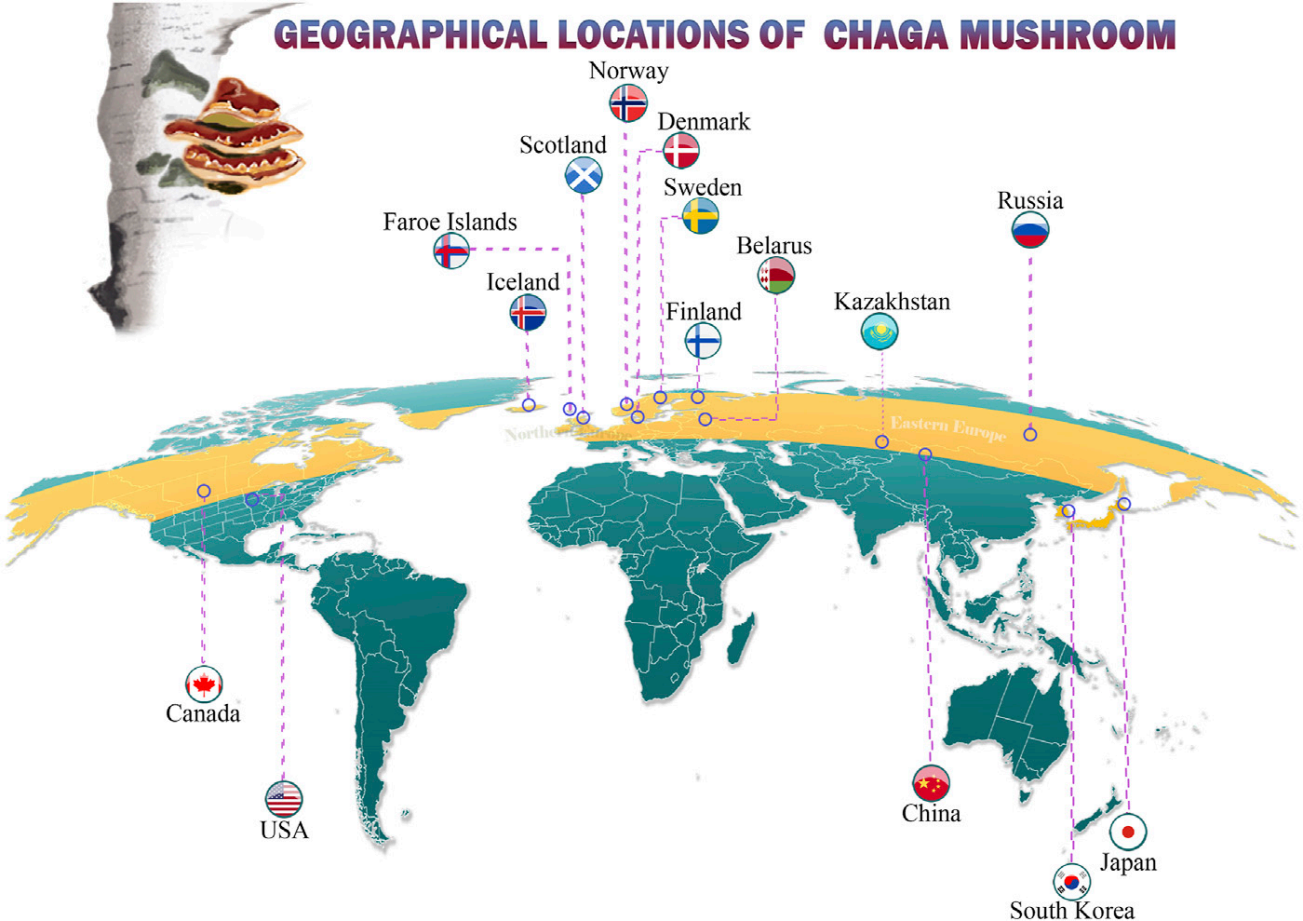
Geographical locations of Chaga mushroom.

Chaga is commonly found in cooler climates, extending from the meridian zone in the mountains to the Northern hemisphere in subarctic regions (Figure 2). This mushroom is well known on three continents, including the cold climate regions of North America at latitudes of 45⁰ N– 50⁰ N (Canada, the United States of America), Asia (Russia, Kazakhstan, Siberia, South Korea, Japan), and Central and Northern Europe. Although Western and Southern Europe have cold climates, Chaga is rare in these regions perhaps owing to the length of unfavorable climatic conditions (Zhong et al., 2009; Abu-Reidah et al., 2021; Wasser, 2021).

## 4 The myth, rituals, and indigenous medicinal properties of chaga mushrooms

In addition to their nutritional value, Chaga mushrooms are featured in various myths and were used by indigenous peoples in the management and treatment of various ailments. These myths and their uses vary among tribes, countries, and localities. In Canada, the Métis, Cree, Ojibway, Denesuline peoples of Northern Saskatchewan, and the Gitksan peoples of British Columbia all have myths about Chaga’s creation (Zmitrovich et al., 2020). It is said that “Wisakecak,” (a mythological character), was responsible for hurling the scab of Chaga onto the birch tree (Rogers, 2016; Peng and Shahidi, 2022). Some historians argue that Wisakecak discovered a birch tree while taking a stroll in the forest. He had been eating a piece of dried meat while wandering through the forest, and as he wandered, he became sleepy and absentmindedly threw the meat onto the tree trunk before settling under the tree and sleeping. While he slept, the meat began to rot, and the spores of the Chaga fungus began to grow on it (Il’ina & Uljašev, 2012). When Wisakecak awoke and saw the Chaga on the tree, he mistook it for the dried meat he had thrown and attempted to consume it. However, he quickly realized his mistake and spat it out, but not before some of the spores had entered his body. He then realized that the Chaga had healing properties and decided to leave it on the tree for the benefit of humanity (Rogers, 2012). In Anishinaabe culture, Chaga is considered a powerful medicinal mushroom and is often used in traditional medicine. Indigenous people also used Chaga mushrooms in pipe rituals because they produce a sweet smell (Zmitrovich et al., 2020). The legend of Wisakecak and the Chaga mushroom is a reminder of the importance of respecting the natural world and the role that traditional knowledge plays in understanding the benefits of natural remedies (Wasser and Volz, 2017).

In Northern Saskatchewan, the Denesuline people use two long strands of Chaga powder to predict the future. The Denesuline would sometimes lay out two rows of powdered Chaga, one for each possible future event. Each line was set on fire at the same time, and whichever one finished first was thought to determine what would happen next (Chung et al., 2010; Peng and Shahidi, 2022). Again, the Denesuline of Saskatchewan used a divination procedure with finely split intestinal fungus (Rogers, 2006). Each pile represented a separate event that occurred at the same time and is illuminated from different ends. The pile that burns through first determines what action takes place initially. “ETSEN DEK, ON”, or “it stinks while it is burning,” is the name given to this occurrence (Rogers, 2006).

**FIGURE 3:**
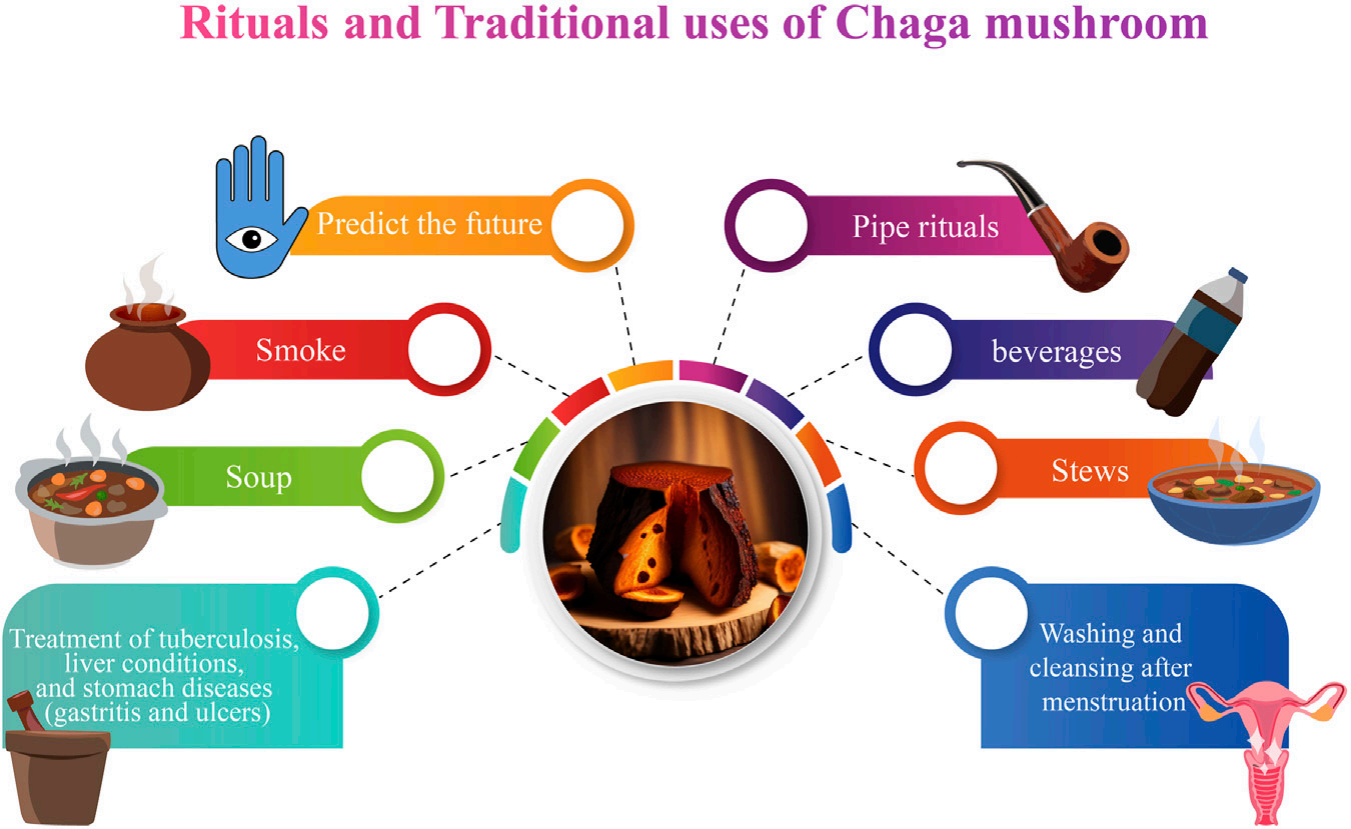
Traditional and ritual uses of Chaga mushroom.

According to historical records (Figure 3), the Khanty people of Western Siberia were the first to use Chaga medicinally, perhaps in the 12th century (Saar, 1991). The native Siberians would grind it up and add it to their everyday beverages, soups, and stews. Despite living in a difficult environment, the Siberians discovered that consuming chaga regularly protected against the beginning of degenerative diseases. They used it to increase vitality and live a long, healthy life. Modern Russians have noticed that cancer is not prevalent where the Chaga was traditionally utilized (Pilz, 2004). According to Moss, (2016), the Khanty tribe regularly consumed Chaga in several forms, including tea, “soap water,” and smoke (Moss, 2016). The tea was produced by slicing Chaga into tiny pieces with a knife, and the pieces were placed into boiling water and simmered for a few minutes. Chaga tea was consumed to aid in digestion, satiate appetite, and detoxify. In the 16th century, the Russian First Nations used Chaga to treat tumors related to angiogenesis. In Siberia, indigenous people used Chaga mushrooms to treat tuberculosis, liver conditions, and stomach diseases (gastritis and ulcers) (Shikov et al., 2014). In modern-day Russia, Chaga is used by hunters and foragers to increase their capacity to work, and to promote endurance (Shikov et al., 2014).

To make “soap water”, the fungus was first placed in the fire. After it becomes charred like smoldering charcoal, it was placed in a bucket of boiling water and swirled until it was broken up into little chunks. The black water that was produced because of this process has excellent cleansing and disinfecting properties (Lukina, 1975). Women who bathed themselves with such water were never sick, and the water was also used to cleanse the vaginal area after menstruation and childbirth. In earlier times, it was also used to wash the hands, feet, and sometimes the whole body in place of soap (Lukina, 1975). Saar (1991) reported that Chaga was also used for ceremonial washing and cleansing after menstruation. It was possible that a newly born child might also be washed as part of this ceremony (Shikov et al., 2014).

In northern America, Chaga is consumed as a tea or powder, inhaled, and smoked by the Indigenous people. “Posahkan” and “Wiskakecak” are the preferred names given by Cree healers due to Chaga’s healing virtues. The Métis also use Chaga for treating cancers of the breast, liver, colon, skin, cervix, and lungs, as well as for diabetes (Barkwell, 2018). Notably, in North America and especially in Canadian indigenous tribes including the Cree, Ojibway, Denesuline, and Gitksan, Chaga was used for the treatment and management of several diseases, including rheumatic and joint pain, infections, and tooth pain (Rogers, 2012). Additionally, Chaga mushroom tea was used by both the Ojibway and the Denesuline people as an antiviral agent. The Tanaina tribe in south-central Alaska in the United States of America also uses Chaga to treat toothaches (Turner and Cuerrier, 2022). The various uses are summarised in Table 1.

**TABLE 1:** Use of Chaga as a folk medicine across cultures.

S/N	Disease and symptoms	Method of administration	Tribe/Country	References
1	Anthelmintic	Tea	Khanty/Siberia	Saar, (1991)
2	Heart disease	Tea		
3	Liver disease	Tea		
4	Disease prevention and death	Smoke		
5	Stomach disease	Tea		
6	Tuberculosis	Tea		
7	Washing of external sexual organs during menstruation and after birth	Soap water		
8	Washing of body	Soap water		
9	Arterial diseases	Chaga infusions	Siberia	Gorbunova et al. (2005)
10	Healing wounds	Lotions-balsams	Siberia	
11	Joint diseases	Chaga syrup	Siberia	
12	Rheumatic pain	Burn a piece of Chaga black coal	Gitksan of British Columbia/Canada	Rogers, (2012)
13	Counterirritant in arthritis (Mugwort)	Moxibustion treatment	Cree/Canada	Rogers, (2012)
14	Toothache	Tea	Tanaina/United States of America	(Turner and Cuerrier, 2022)

In China and Korea, empirical evidence suggests that for centuries, Chaga was traditionally taken as a tea to treat several ailments including pathogenic infections, gastrointestinal disorders, cancers, and liver disorders (Park et al., 2004; Géry et al., 2018; Gründemann et al., 2020). Similarly in Japan, Greece, and parts of eastern Europe, the mushroom has a history of use in folk medicine as a treatment for ulcers, gastritis, and tuberculosis (Cha et al., 2006a; Pan et al., 2013). It was recorded that the Grand Duke of Kievan Rus, Tsar Vladimir Monomakh, used Chaga to heal his lip tumors. Finnish soldiers used Chaga as a coffee substitute during World War II (Baek et al., 2018). When the troops’ supply of coffee ran short, they turned to the nearby woodlands for sclerotia, which they used to make Chaga tea.

**FIGURE 4:**
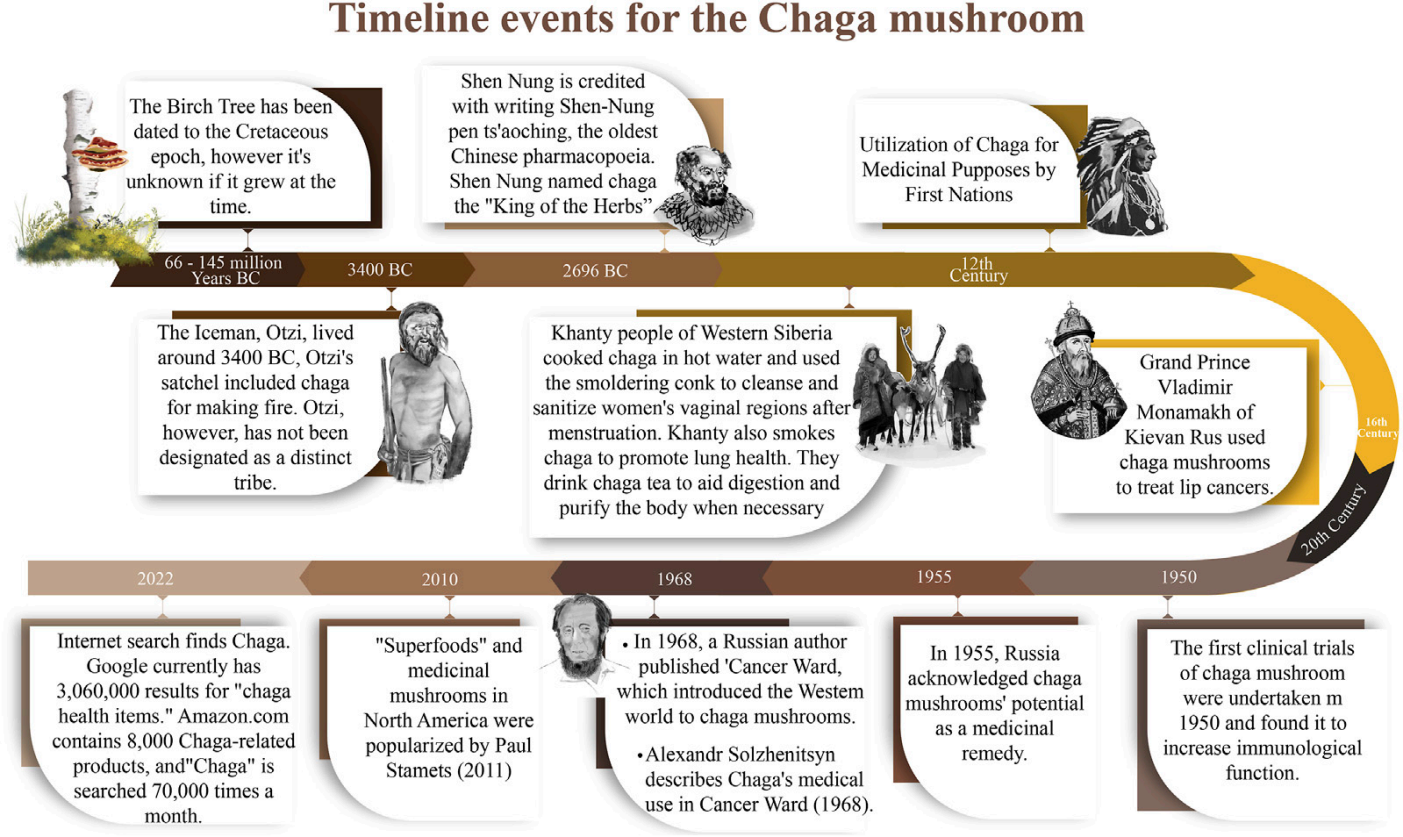
Timeline events for the Chaga mushroom.

According to Gorbunova et al. (2005), inhabitants of Siberia have long relied on Chaga infusions for medicinal purposes. Indeed, the fact that Befungin was the first Chaga product to be isolated and used in clinical therapy has been proven by Russian experts and is now known to be an effective treatment for psoriasis. It is worth mentioning that in the middle ages, the famed physician Avicenna experimented with the use of Chaga as a source of medicinal compounds (Ryzhova et al., 1997; Safin et al., 2018). Various traditional medicinal uses of Chaga around the world are summarized below (Figure 4).

## 5 Morphological characteristics

**FIGURE 5:**
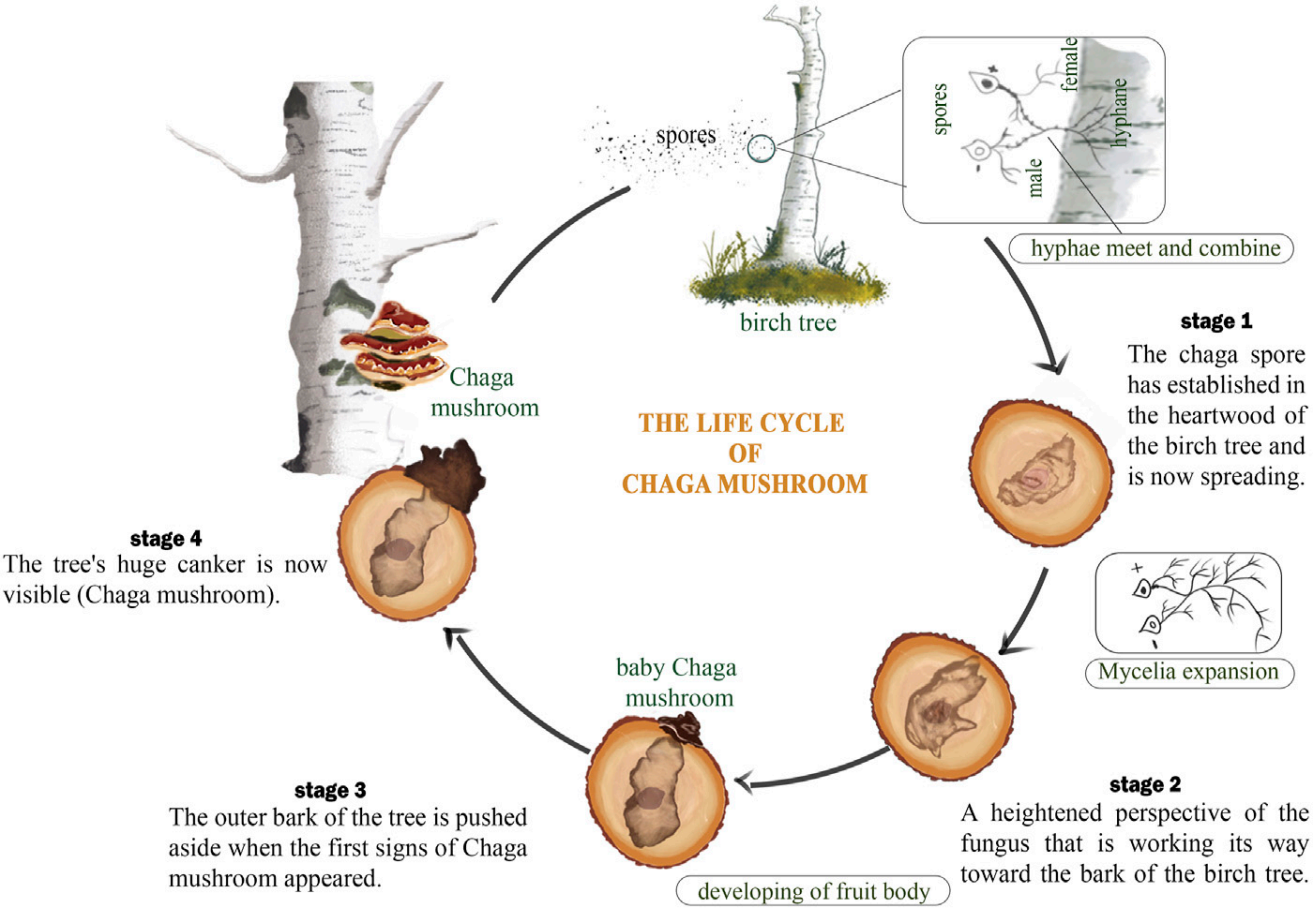
The life cycle of Chaga mushroom.

Chaga mushroom usually grows on the bark of birch trees. This association matches the Cree legend that describes a type of scab growing along the trunk of birch trees (Rogers, 2012). Chaga has a texture similar to loose, rubbery wood or porous, crumbly rocks (Hobbs, 2003). The Chaga is not the fruiting body as seen in other mushrooms, it is the sclerotia that contains the mycelium. Chaga is a parasitic fungus that infects birch trees and looks like hard rock protrusions (Kim, 2005). Close examination using a microscope shows a brown hypha separated without pores as well as a wide partition with 2.five to seven um in diameter. Chaga arises through the action of basidiospores contaminating the duramen of birch trees through unhealed wounds (Brydon-Williams, 2019). Basidiospores, which are reproductive structures produced by the Chaga fungus, can enter the tree through these wounds and then grow and spread within the tree’s inner tissues, or duramen (Figure 5). Over time, the Chaga fungus can form large, woody growths or cankers on the tree’s surface, which are then harvested and processed for various uses (Thomas et al., 2020). The large woody growth is supported by root-like structures of the mushroom known as the mycelium. This structure grows inside the tissues of the tree and as it grows degrades the cell wall of the tree while simultaneously producing decay known as ‘white rot’ (Figure 5). The white rot degrades cellulose, hemicellulose, and lignin the major components found in the cell wall. By breaking down these materials, the Chaga fungus can extract nutrients from the tree and continue to grow and spread throughout its life (Deshpande and Arya, 2022).

The fruiting bodies of Chaga, which appear in the form of black, irregularly shaped masses, develop between the bark and the sapwood of the tree and turn yellow to brown over time. This transformation usually takes between 2 and 12 years after the death of the birch (Bernicchia, 2005; Géry et al., 2018). Although the fruiting body of Chaga is usually harvested, the vegetative component rather contains more proteins as compared to the fruiting body (Rogers, 2006). The sclerotia of Chaga contains up to 30% betulin, a compound that is also found in birch trees. This betulin is believed to be absorbed by the Chaga fungus from the birch tree, where it is present in the form of betulinic acid. In addition to betulin, the Chaga core contains a greater amount of lanostanes, which are triterpenoids that have been shown to have a variety of potential health benefits (Wold et al., 2018). Betulinic acid possesses biological activities such as anti-cancer, anti-inflammatory, antiviral, and antioxidant (Hordyjewska et al., 2019).

The high concentration of melanin in the sclerotia (the hard, black outer layer of the Chaga mass) gives it its characteristic black color (Stamets, 2005). The sclerotia contain up to 30% betulin, while the core contains a greater amount of lanostanes. However, the harvested part of the Chaga is bicolored, consisting of the sclerotia and a golden, cork-like interior. As the Chaga mass ages, a greater proportion of its mass becomes associated with the interior rather than the sclerotia (Figure 5) (Thawthong et al., 2014).

## 6 Mycochemistry

Mycochemical analysis of Chaga extracts revealed several essential primary metabolites including polysaccharides, proteins, and other compounds (Kim et al., 2011; Peng and Shahidi, 2022). As of 2022, more than 250 secondary metabolites have been identified in chaga including betulin, vanillic acid, terpenoids, and lanosterol. Rogers (2006) further reports that the fungus is made up of primary metabolites including minerals, proteins, and polysaccharides. Several macro and micro nutrients have been identified from Chaga extracts which include carbohydrates (*β*-glucans, xylogalactoglucose) (Joo et al., 2010; Jayachandran et al., 2017; Wold et al., 2018), lipids (fecostrol, episterol, *β*-sitosterol) (Cha et al., 2005; Cha et al., 2006b), polyphenols (inonoblins A, phelligridins D, ferulic acid, foscoperianol D, vanillic acid) (Lee et al., 2007; Seo and Lee, 2010).Some of these compounds are presented below:

### 6.1 Polysaccharides

Huang et al. (2012) extracted and purified five *Inonotus obliquus* polysaccharides (IOPS) by column chromatography, namely,; IOP1b, IOP2a, IOP2c, IOP4 and IOP3a (Huang et al., 2012). In one study, the authors suggest that the sugar, -(1.3)- *β-*D-mannan influences *β*-glucan properties (Baek et al., 2012). Recently, both *β*-glucan has been found to have a variety of health-promoting properties, including immune modulation, anti-inflammatory effects, and antioxidant activity (Zhu et al., 2015). Yeast, mushrooms, bacteria, and algae are all sources of a dietary fiber compound called β glucan. This compound is widely recognized as a polysaccharide found in Chaga mushrooms along, with galactomannan (Basal et al., 2021; Eid et al., 2021; Peng and Shahidi, 2022). Chaga contains 8.57% *β*-glucan (Song 2020).

### 6.2 Proteins

According to a study, by Razumov et al. (2020), they found that chaga hydrolyzed products contain many amino acids with aspartic acid, glycine, and glutamic acid making up, around 40% of the amino acids. The remaining 60% consists of lysine, threonine, methionine, alanine, tyrosine, serine, histidine, proline, tryptophan, arginine, and cysteine (Razumov et al., 2020). Other forms of amino acids such as peptides have also been reported by Hyun et al. (2006). In the study, the author identified a new peptide that prevents platelet aggression. In the study, analysis of the peptide using LC/MS revealed a molecular mass of 365 Da (Hyun et al., 2006).

### 6.3 Mineral components

Chaga has been observed to have a high concentration of elements, with potassium accounting for 50% of the total ash content, sodium accounting for 9%–13%, and manganese accounting for 1.2% (Shashkina et al., 2006). Other minerals such as copper, zinc, aluminum, sulfur, magnesium, phosphorus, and calcium have all been identified in chaga (Razumov et al., 2020).

Chaga also contains a significant number of mineral microelements. In their research, the scientists used x-ray fluorescence and atomic absorption spectroscopy to analyze chaga and determine its composition of macro and micro elements. Their findings revealed that chaga contains amounts of iodine, vanadium, zinc, copper, selenium, manganese, and sulphur (0.02%) rubidium (approximately 0.04%) sodium (approximately 0.05%) phosphorus (approximately 0.23%) chlorine (approximately 0.33%) calcium (approximately 0.37%) nitrogen (approximately 0.4%) magnesium (approximately 0.64%) hydrogen (around 3.6%) potassium (around 9 10%) and carbon constituting approximately 39%. (Razumov et al., 2020). These findings suggest that Chaga mushrooms and their derived products possess essential nutrients that are beneficial for crop cultivation (Peng and Shahidi, 2022).

### 6.4 Bioactive compounds

Based on several studies of crude Chaga extracts, numerous bioactive compounds have been isolated and purified (Table 2).

**TABLE 2:** Compounds isolated from Chaga.

Compounds	Structure	References
Polyphenols		
Phelligridin C	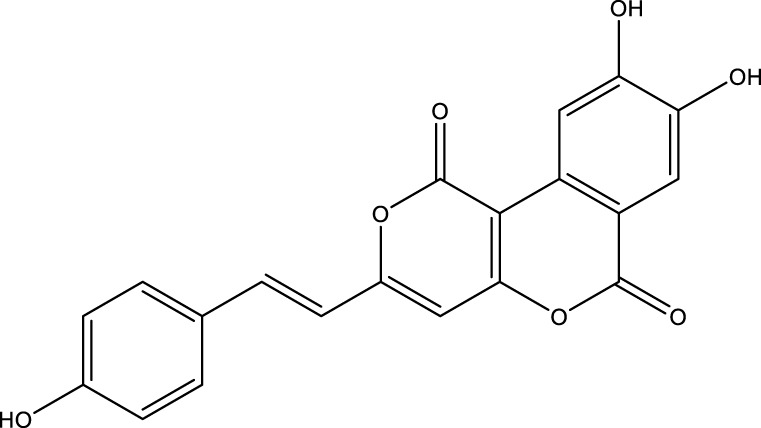	Lee et al. (2007), Zheng et al. (2011b)
Phelligridin D	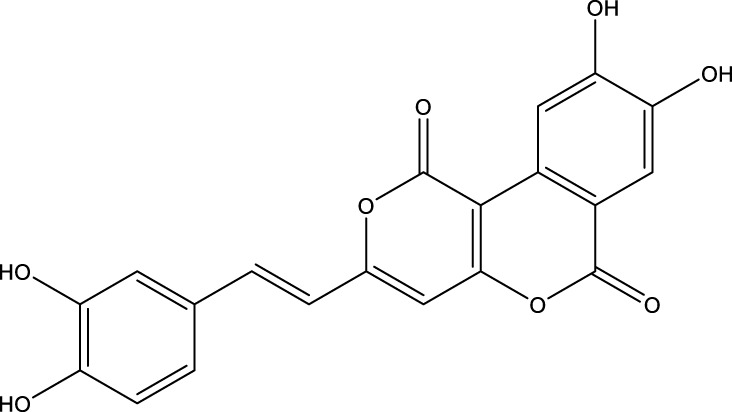	Lee et al. (2007)
Phelligridin E	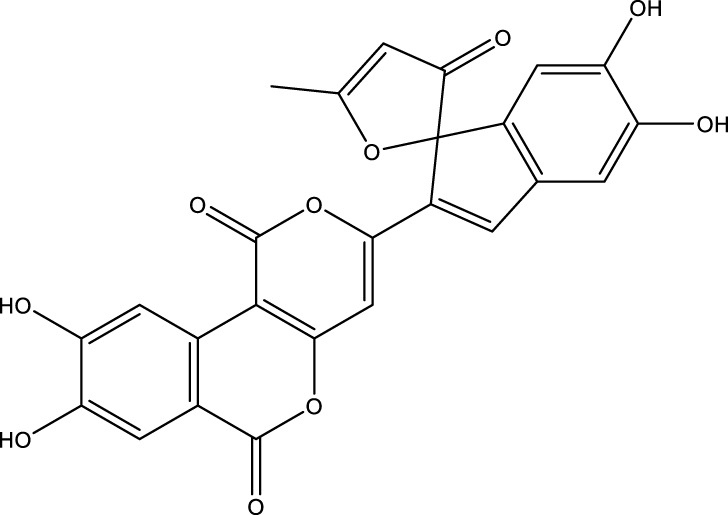	Lee et al. (2007)
Phelligridin F	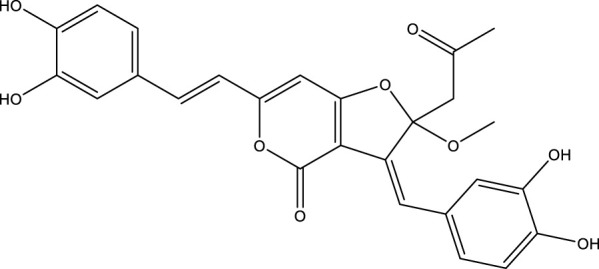	Lee et al. (2007)
Phelligridin G	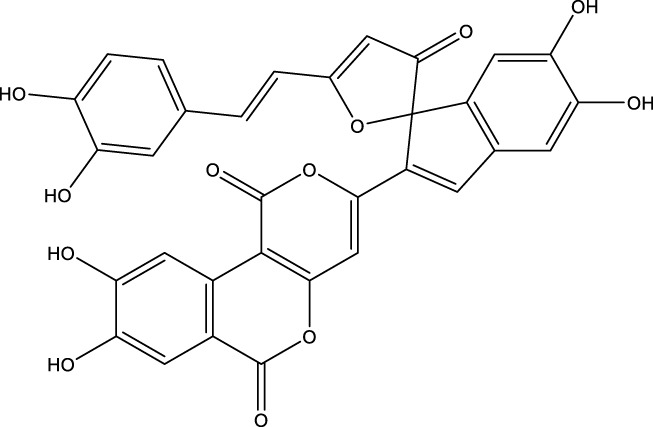	Lee et al. (2007)
Phelligridin H	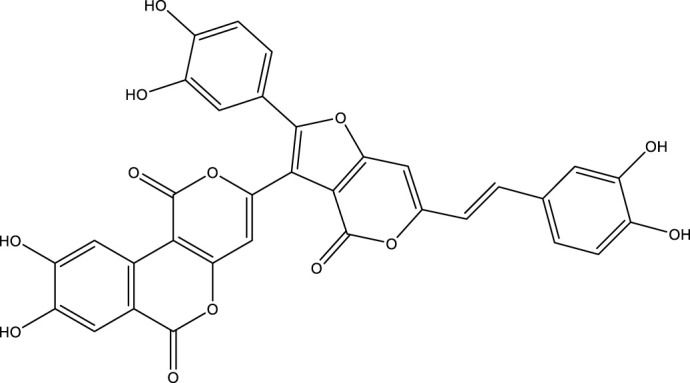	Lee et al. (2007)
Inonoblin A	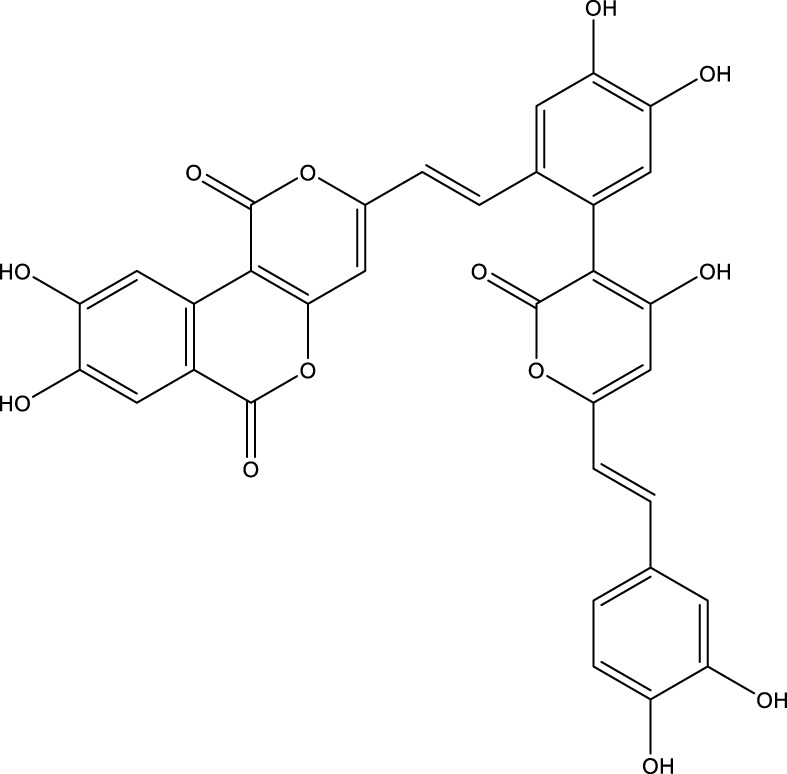	Lee et al. (2007)
Inonoblin B	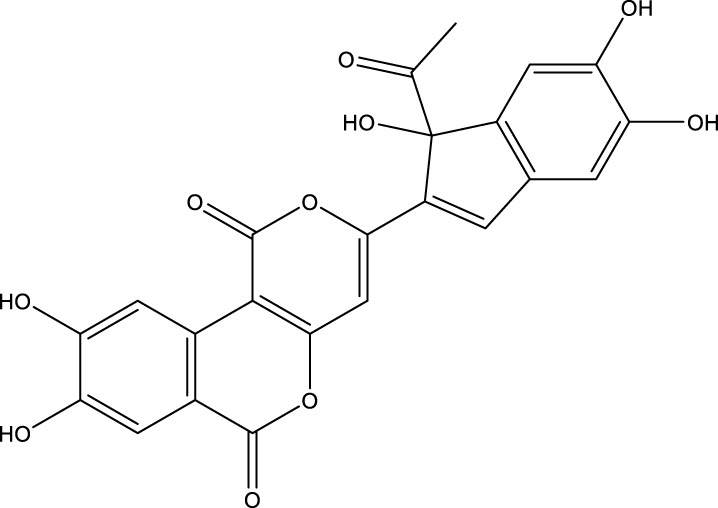	Lee et al. (2007), Zheng et al. (2011a)
Inonoblin C	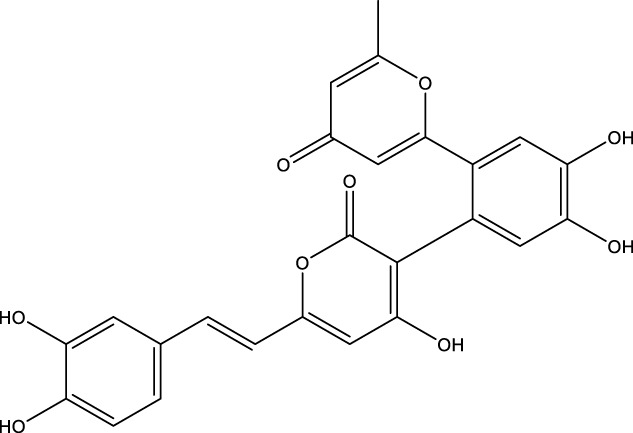	Lee et al. (2007), Zheng et al. (2011b)
Methylinoscavin A	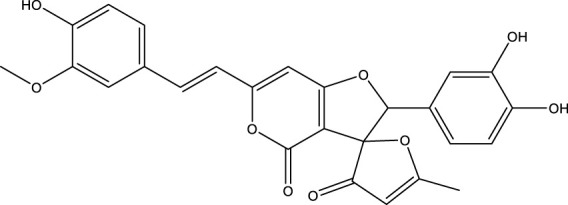	Zheng et al. (2011a)
Terpenes		
Inonotsutriol A	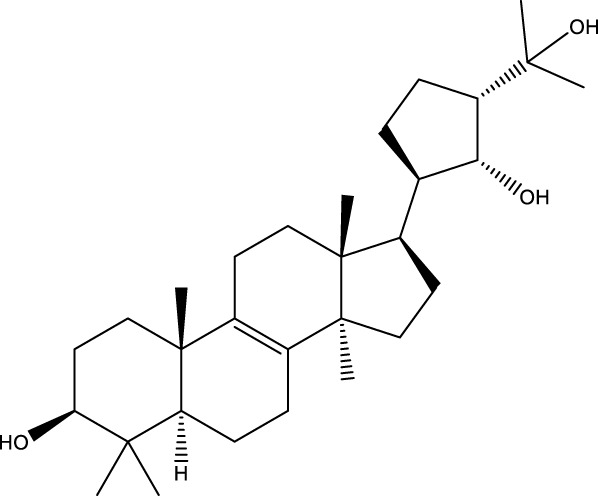	Chang et al. (2022), Zhao et al. (2015)
Inonotsutriol B	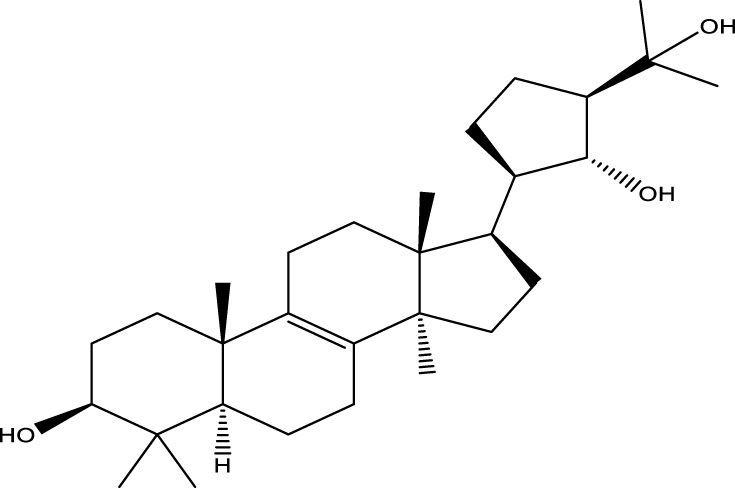	Zhao et al. (2015), Park et al. (2021)
Inonotsutriol C	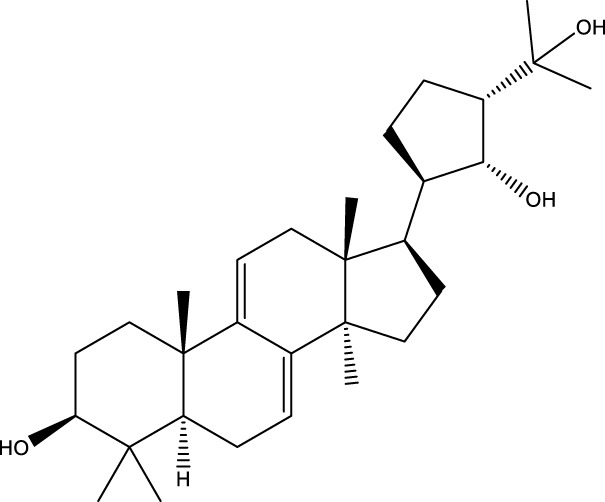	Tanaka et al. (2011)
Ergosterol	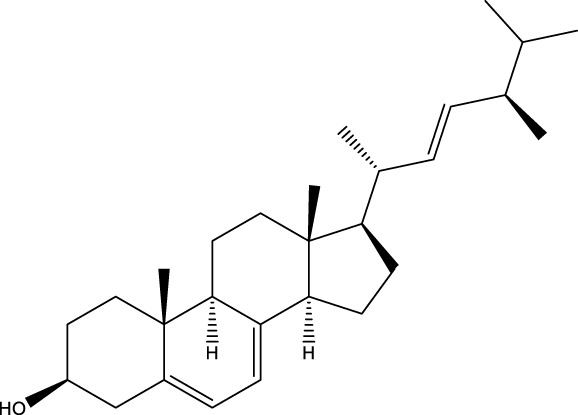	Zhao et al. (2015)
Lanosterol	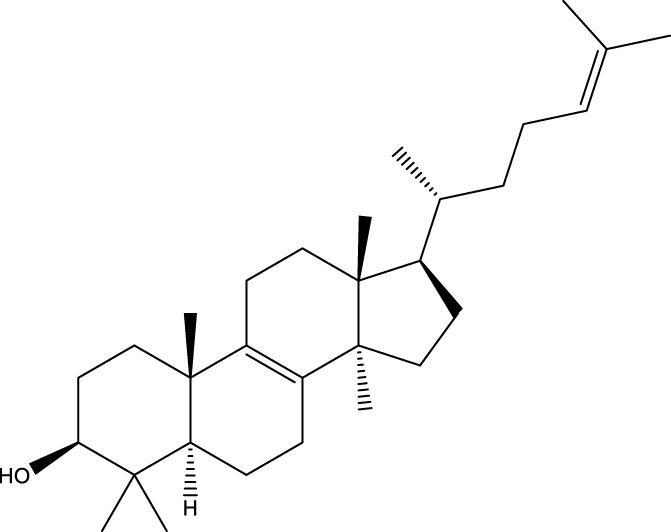	Moon and Lee (2009); Ma et al. (2013)
Betulin	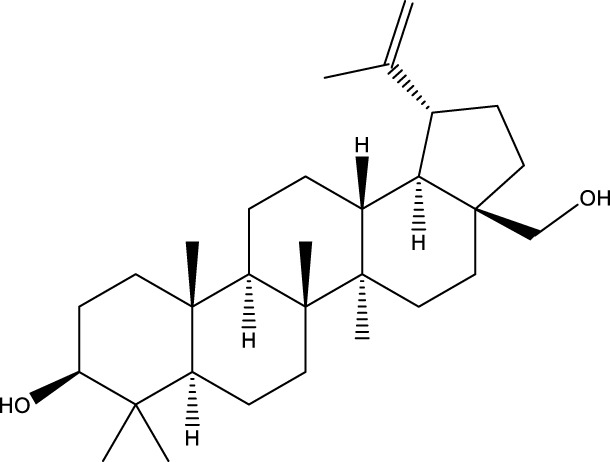	Wold et al. (2018)
Betulinic acid	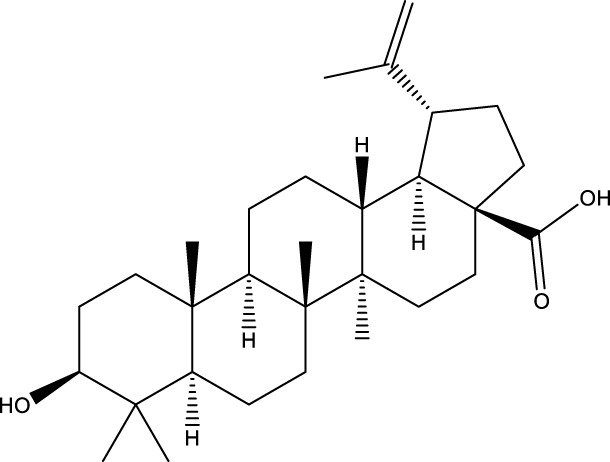	Zhao et al. (2016)
Inonotsuoxide A	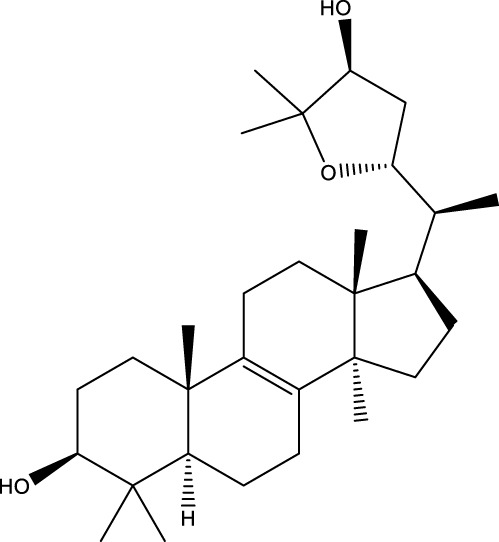	Zhao et al. (2015)
Inonotsuoxide B	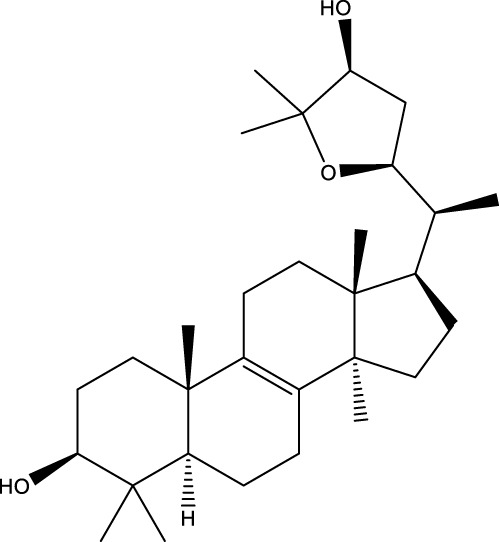	Tanaka et al. (2011)
Inonotusane C	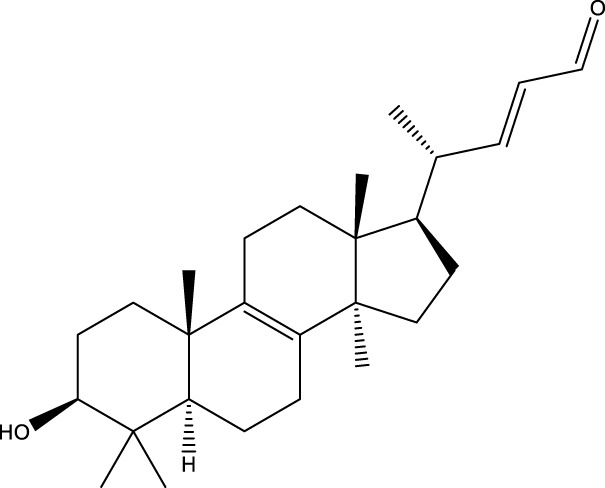	Zhao et al. (2015)
Terpenoids		
Stigmastanol/sitostanol	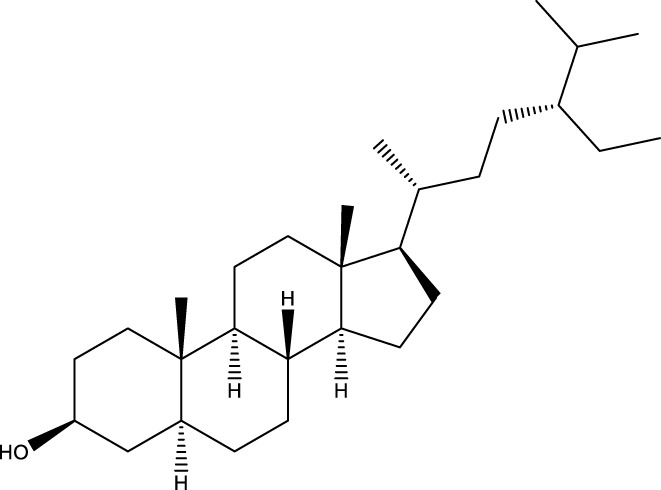	Sun et al. (2008)
Lupeol	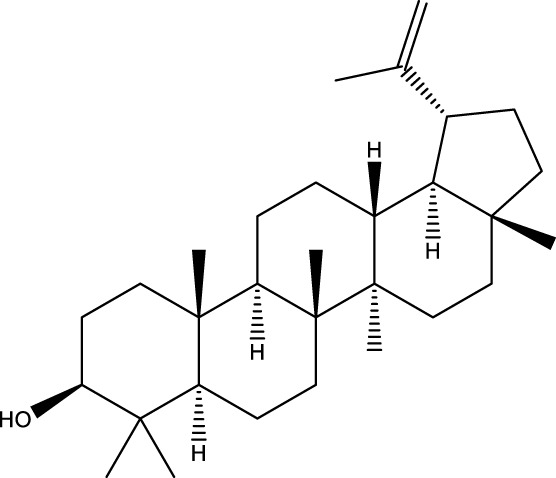	Sun et al. (2008)
Lupenone	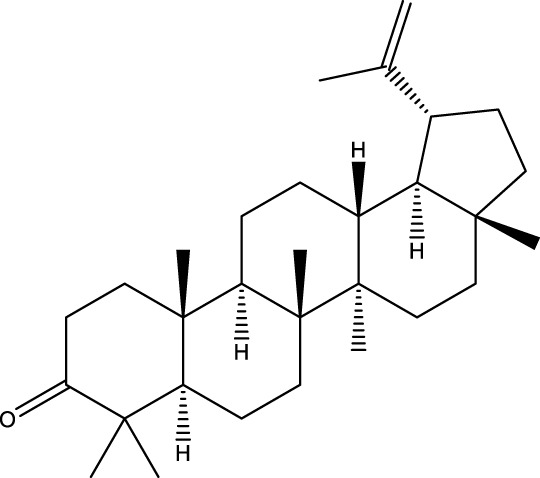	Sun et al. (2008)
α-Curcumene	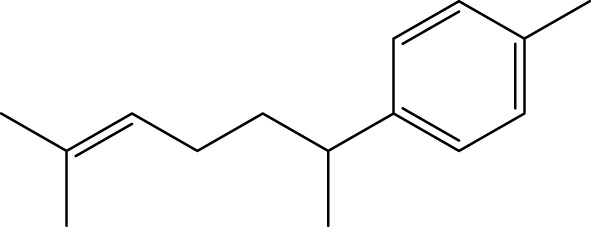	Zhao et al. (2016)
α-Cedrene	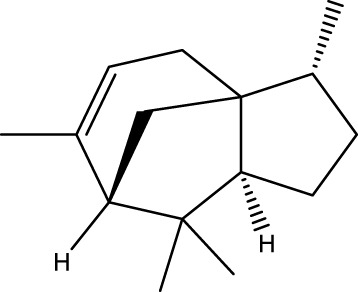	Zhao et al. (2016)
β-Farnesene	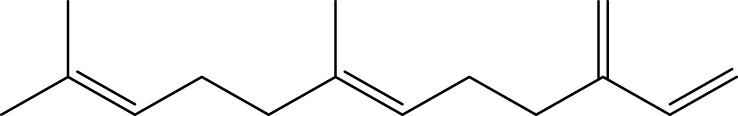	Ying et al. (2014)
α-Bisabolene	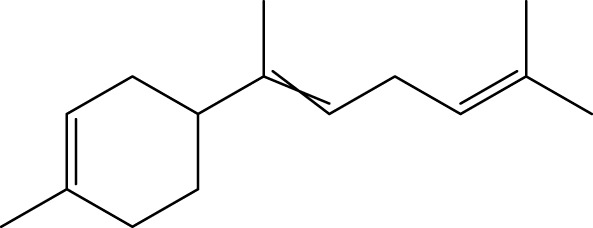	He et al. (2001), Thomas et al. (2020)
*p*-Cymene	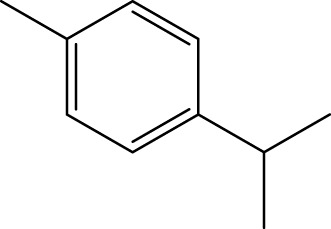	Geng et al. (2013)
Photosantalol	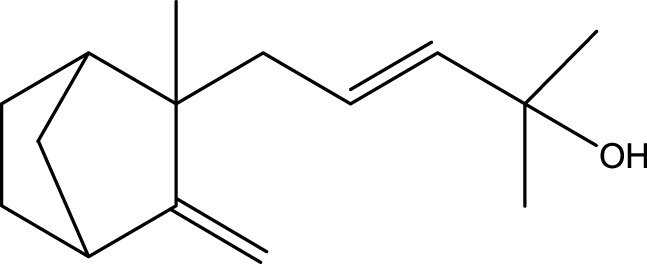	Ayoub et al. (2009)
Triterpenoids		
Lanosterol	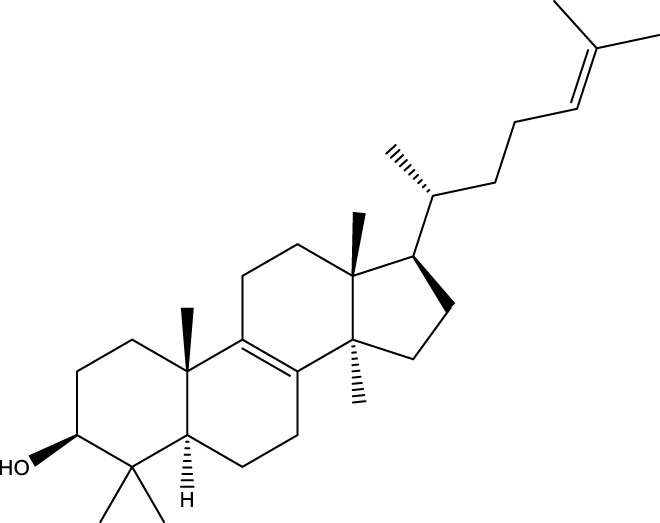	Liu et al. (2014)
3 β - hydroxylanosta-8.24,dien-21-al	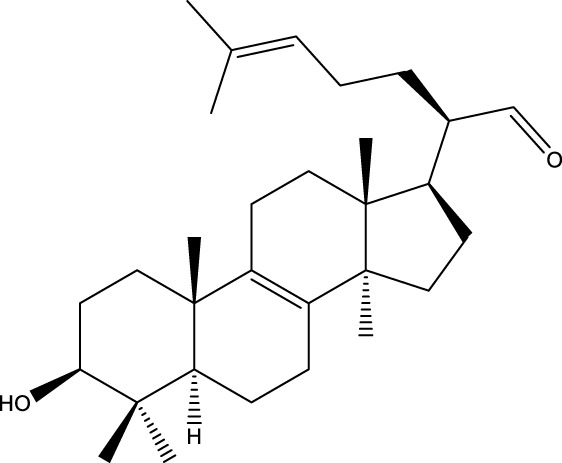	Kahlos et al. (1984)
Inotodial	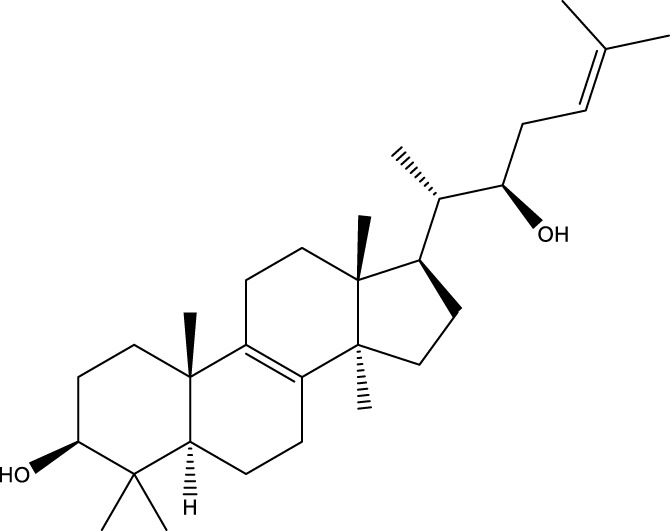	(Yusoo et al., 2002)
Trametanolic acid	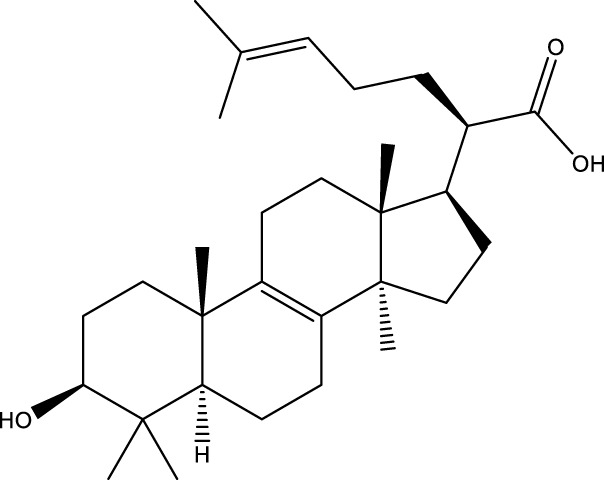	Nakata et al. (2007)
Lanosta-7.9 (11),24-trien-3-β-22,diol	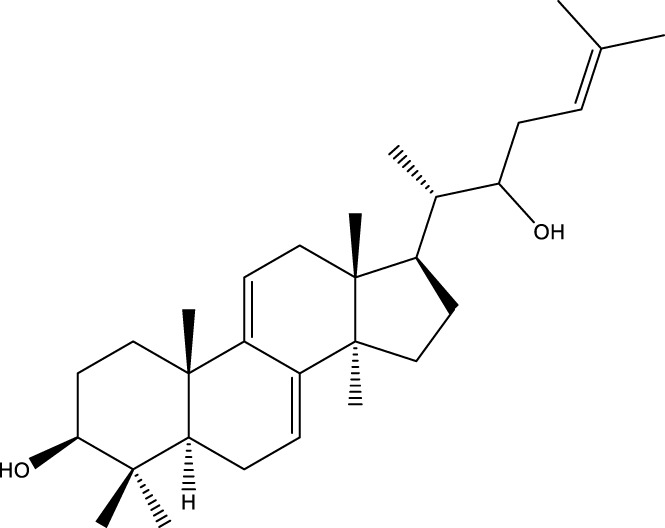	Taji et al. (2008)
Lanosta-8,23E-dien-3 β,22R,25-triol	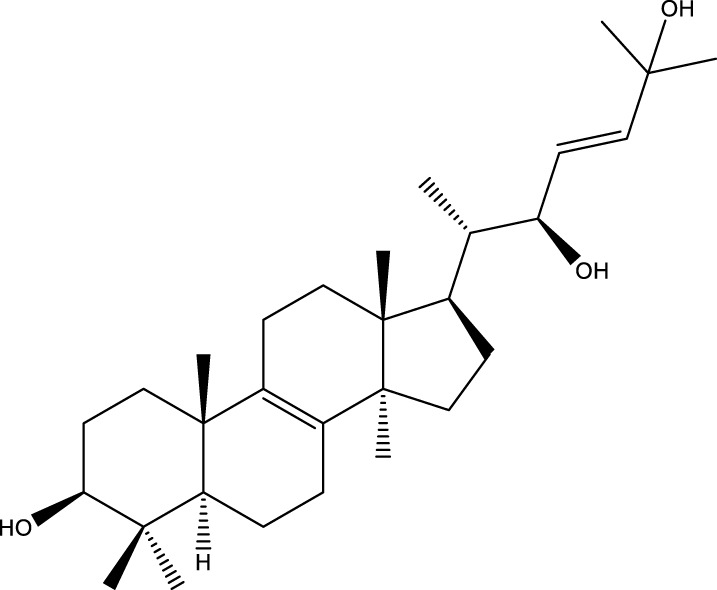	Taji et al. (2008)
Lanosta-7.9 (11),25E-trien-3 β,22R,25-triol	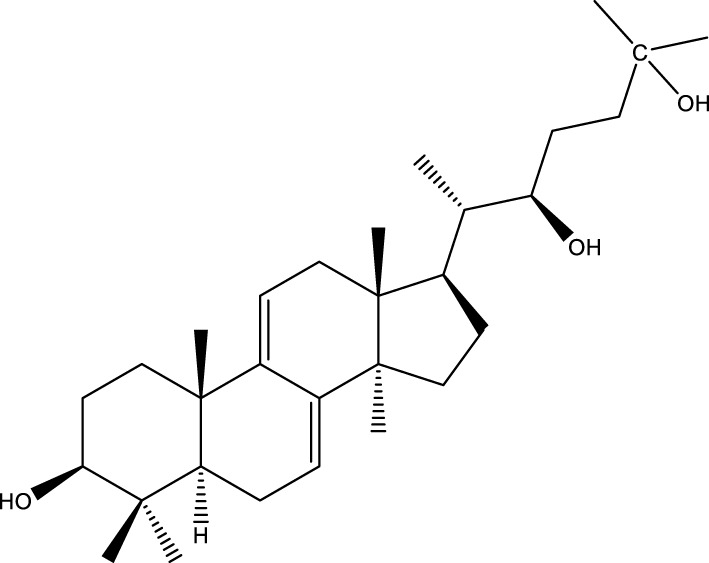	Nakajima et al. (2007)
Lanosta-8-24-dien-3 β −21-diol	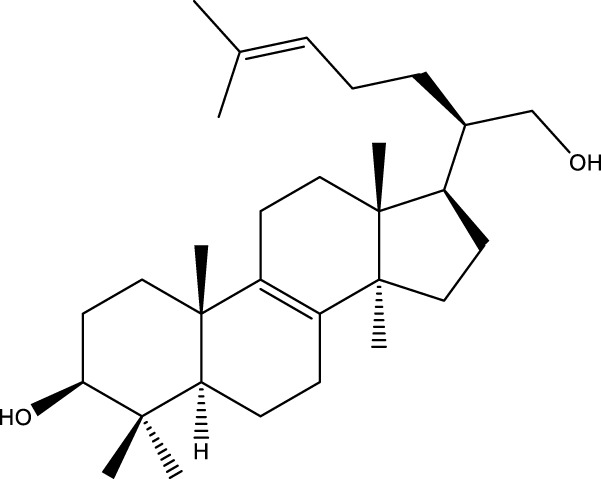	Handa et al. (2010)
Inonotusol A	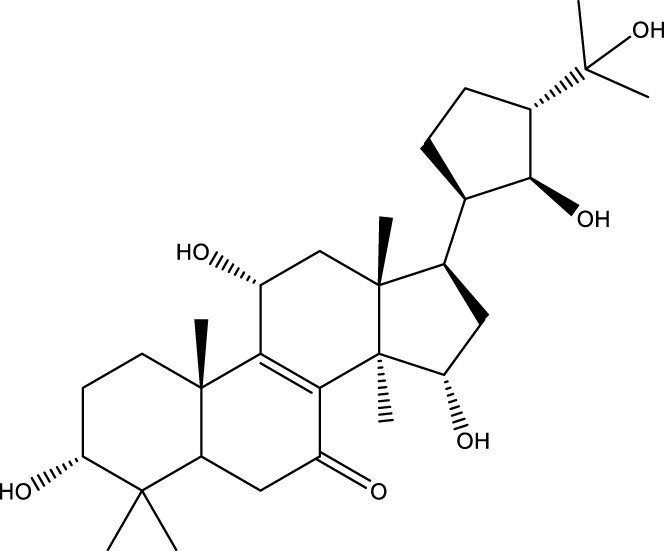	Handa et al. (2010)
Inonotusol B	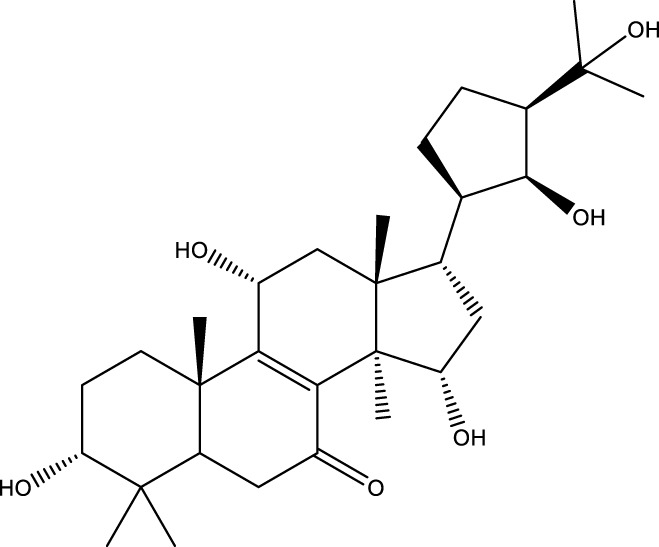	Handa et al. (2010)
Inonotusol C	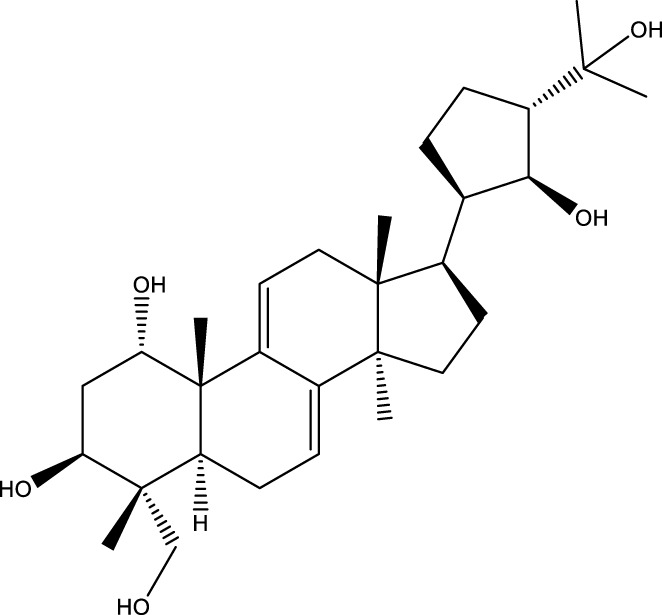	He et al. (2001)
Inonotusol D	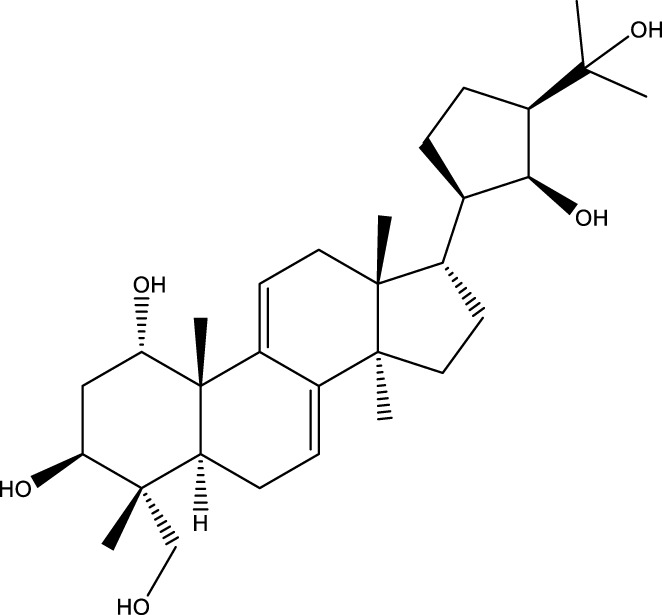	Kim et al. (2011)
Inonotusol E	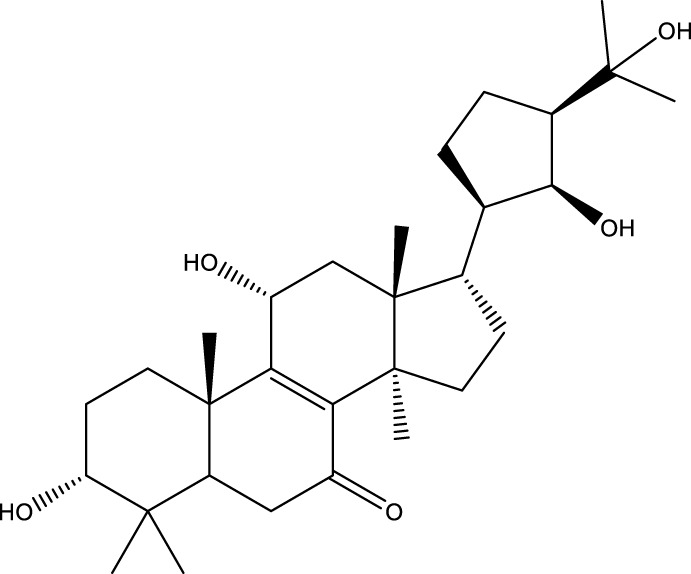	Kim et al. (2011)
Inonotusol F	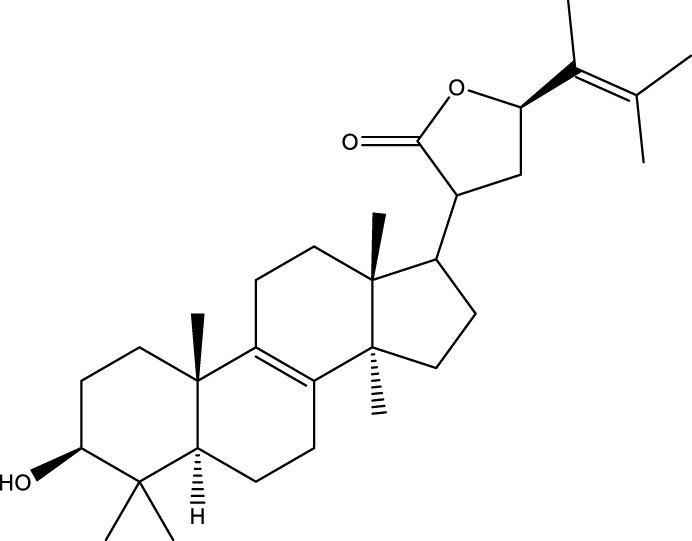	Kim et al. (2011)
Inonotusol G	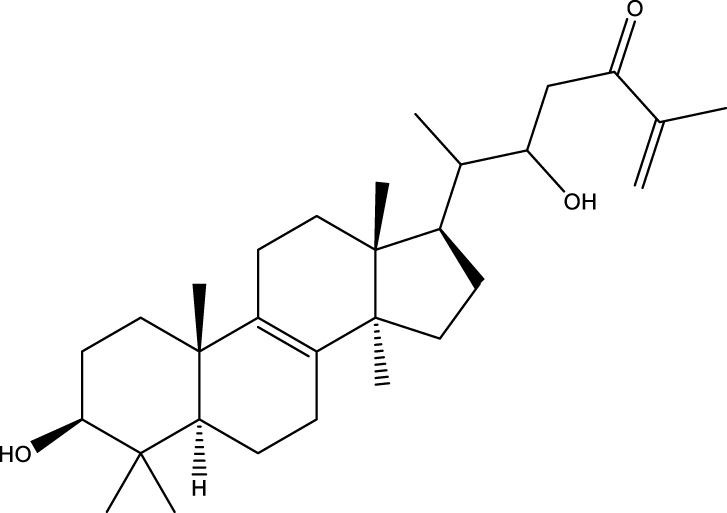	Kim et al. (2011)
3β −22-dihydrolanosta-8-24-diene-7-one	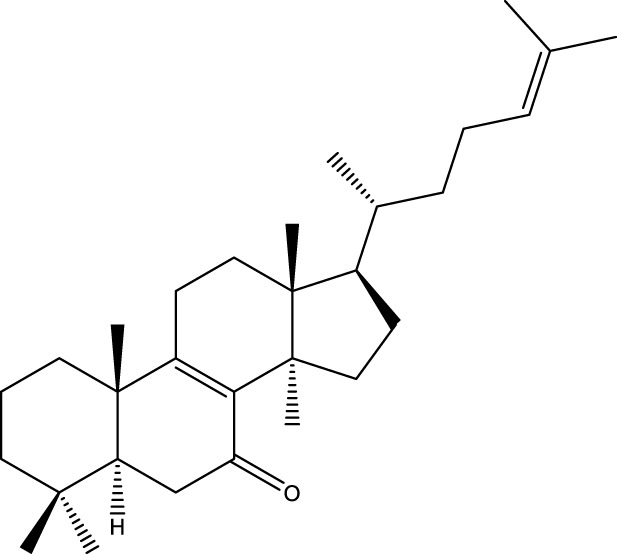	He et al. (2001)
3 β -hydroxylanosta-8,24-diene-21,23-lactone	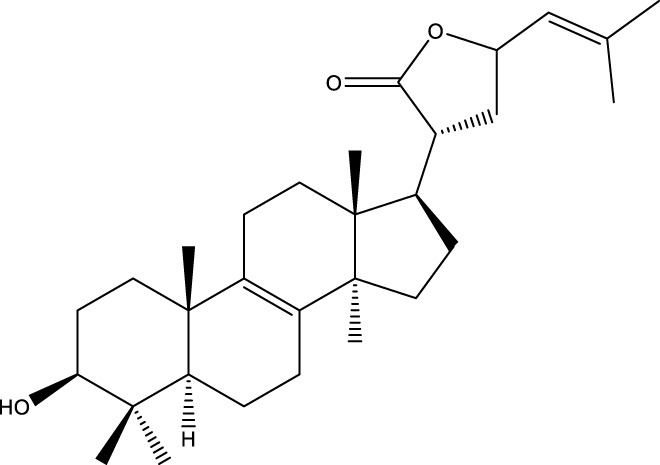	Kahlos et al. (1984), Nakata et al. (2007)
Methyl trametenolate	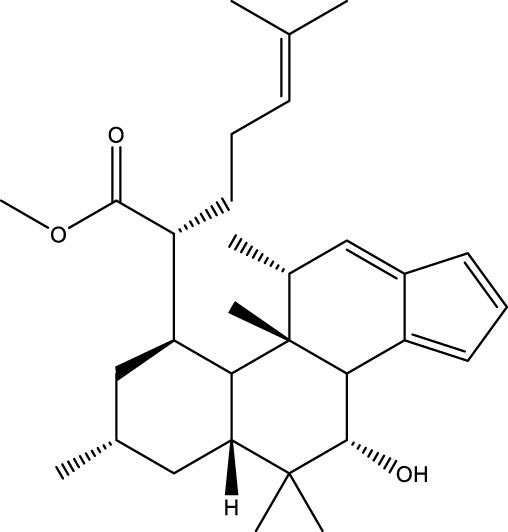	Ying et al. (2014)
21,24-cyclopentalanosta-8-en-3 β -21-25,triol	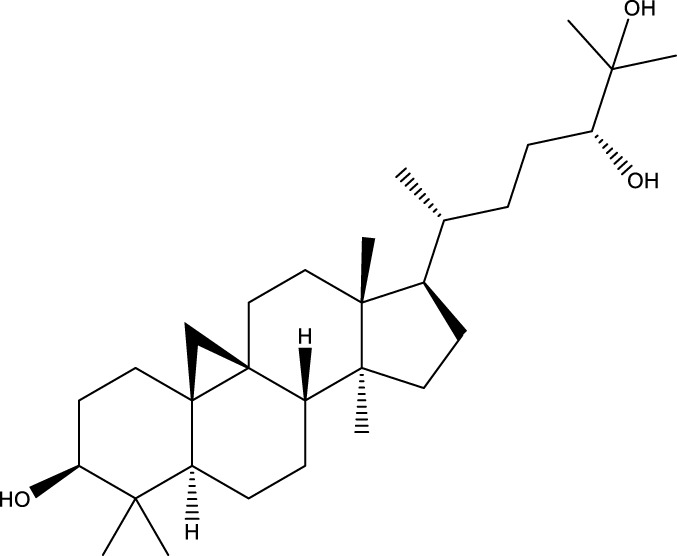	Tanaka et al. (2011)
Lanosta-8-en-3 β- 22.25,triol	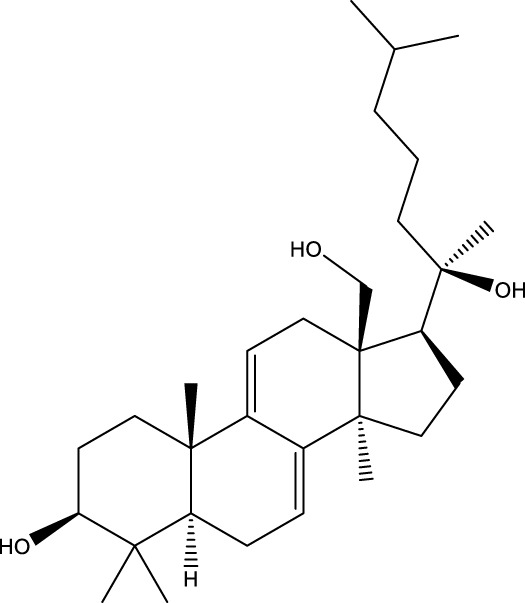	Zheng et al. (2011b)
Sterols		
Ergosterol peroxidase	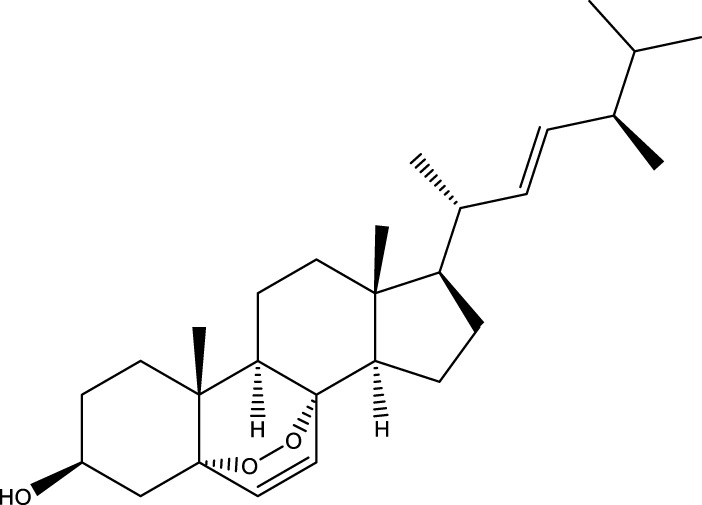	Ma et al. (2013)
*β*-sitosterol	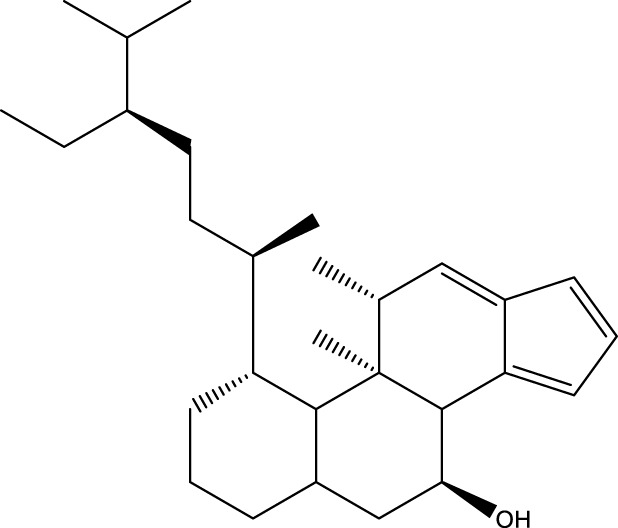	Lu et al. (2010)
Cholesterol	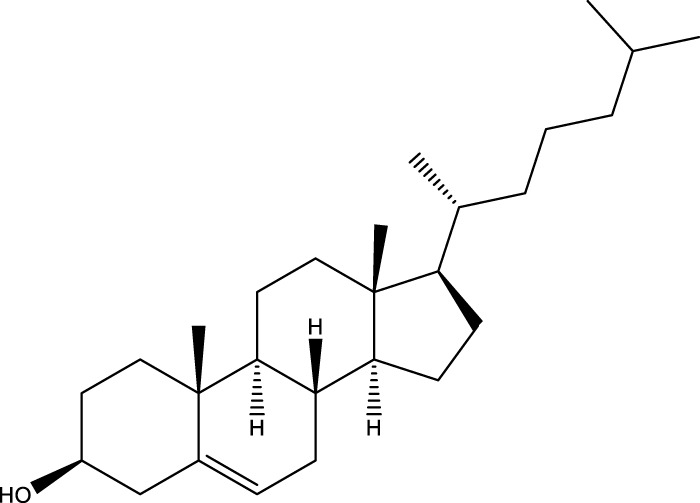	Ma et al. (2013)
Fungisterol	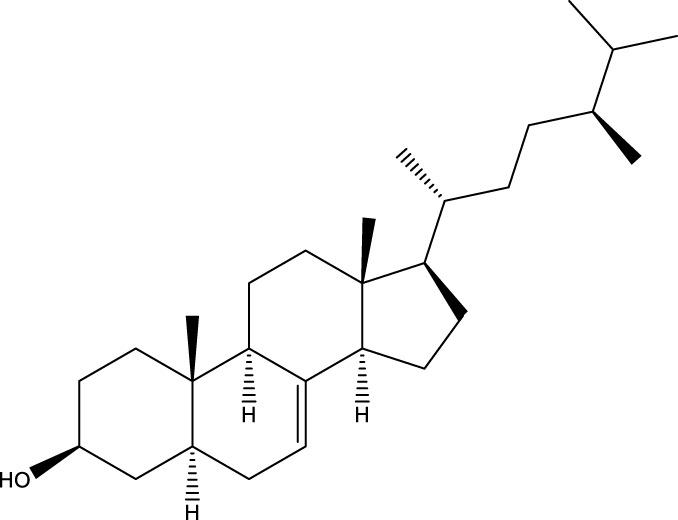	Tanaka et al. (2011)
Episterol	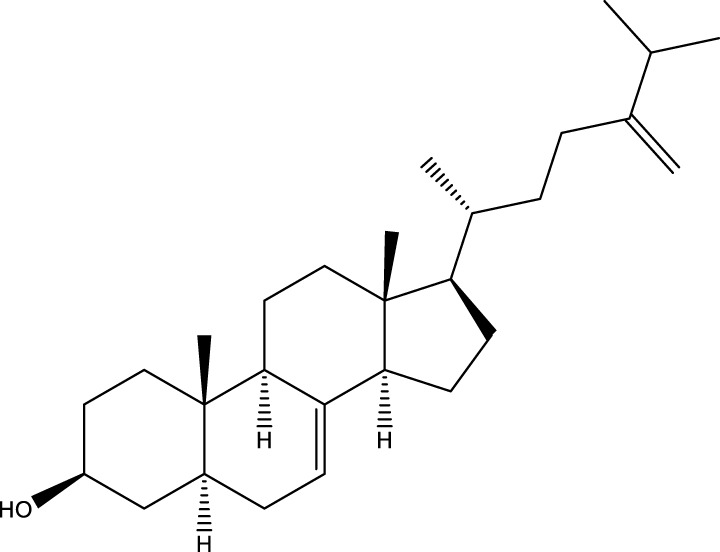	Tanaka et al. (2011)

#### 6.4.1 Polyphenols

Six polyphenols were recovered from Chaga mushrooms following extraction with methanol: inonoblins A, B, C, and phelligridins D, E, and G (Table 1) (Lee et al., 2007). Particularly, chaga contains a variety of phenols, including 4-hydroxy 3,5 dimethoxy benzoic acid, 2-hydroxy-1-hydroxymethyl ethyl ester, protocatechuic acid, caffeic acid, 3,4 dihydroxy benzene formaldehyde, 2,5-dihydroxyterephthalic acid, syringic acid and 3,4-dihydroxybenzalacetone (Nakajima et al., 2007; Nakajima et al., 2007). Additionally, gallic acid and dihydroxy benzoic acid ketones (DHBAs) have been found in Chaga. Show potential in their ability to combat cancer (Kim et al., 2011). Melanins are also generated by Chaga, which exhibit antioxidant and genoprotective properties (Shashkina et al., 2006). Babitskaya et al. (2000) demonstrated that copper ions, catechol, and tyrosine effectively promote the production of melanin, and *o*- and *p*-dephenoloxidase. The acid composition of Chaga includes acetic acid, butyric acid, oxalic acid, and formic acid (Razumov et al., 2020). Chaga extract also contains triterin, sterols (6%–8%), acid-resistant lignin (25%–30%), dietary fiber (2%), hemicellulose (12.5%), and folic acid (Shashkina et al., 2006).

Zheng et al. (2009) conducted a study to extract phenolic components from I. Obliquus by using fermentation. They used continuously stirred reactors to investigate how phenolic compounds accumulated in various cultures and their antioxidant properties. According to the study, Chaga demonstrated the production of melanin and polyphenols in the control medium. The introduction of H_2_O_2_ did not have an impact on the levels of phenols. It did increase the amount of phenol and melanin inside the cell. When Chaga was exposed to both H_2_O_2_ and arbutin there was an increase in phenol synthesis while extracellular phenol accumulation decreased. These findings suggest that incorporating an oxidizing agent into Chaga mycelia could boost the production of compounds such, as vanillin, salicylic acid, pyrocatechol, hydroquinone, and eugenol. This makes Chaga a more dependable source of pharmaceutically essential phenolic compounds.

Chaga contains numerous flavonoids, such as flavones, flavanones, anthocyanins, and catechins (apigenin, mariningin, corin, and quercetin) (Shashkina et al., 2006). As of 2022, a total of 31 flavanoids were detected in a Chaga sample (Peng and Shahidi, 2022). When the phenolic contents of mycelia and wild chaga culture were compared, flavonoids were detected in small quantities in wild Chaga, while other phenols such as melanins and styrylpyrones were present in larger quantities. In contrast, the mycelia cultures contained more flavonoids with trace quantities of melanin and styrylpyrones (Zheng et al., 2008). The Chaga mushroom contains a unique class of phenolics known as styrylpyrones (Peng and Shahidi, 2022). A total of 16 styrylpyrones have been detected previously, including phelligridin C, inoscavin C, hispidin, methylinoscavin C, davallialactone, inonoblin C, davallialactone, inonoblin B, phelligridin E, phelligridin D. Newly detected compounds included: inoscavin A, inoscavin D, phelligridin J, phelligridin A, phelliribsin A, methylinoscavin D.

Peng and Shahidi (2022) further detected several flavonoid derivatives such as apigenin, eriocitrin, rhoifolin, isorhamnetin-3-O-rutinoside, epigallocatechin, and epigallocatechin. However, other non-flavanoid phenolics such as inonoblin A, phelligridin, resveratrol, and phellxinye A were absent. In the same study, the 31 detected flavonoid derivatives included 18 flavones (ols), 5 flavanones (ols), 3 flavans (ols), aurones, isoflavones, ligniflvanoids and chalcones (Razumov et al., 2020).

#### 6.4.2 Steroids

Steroids are another bioactive component of Chaga (Wang et al., 2014), including fungal sterols (Kirk et al., 2008; Chen and Wang, 2014), ergosterol (Shin et al., 2000; Huynh, 2019), ergosterol peroxide (Zheng et al., 2011a; Kim et al., 2011), and *β*–sitosterol (Table 1). Wang et al. (2014) found that (0.01 and 0.1 g/L) dosages of extracts from birch bark and birch core had a substantial stimulatory impact on the generation of *I. obliquus* steroids in submerged Chaga cultures (*p* = 0.05). The aqueous extract (0.01 gL) of birch bark stimulated 97.3 percent greater production than the control (i.e., 225.8 mg/L). Although these extracts promoted both mycelial growth and steroid content, only methanol extracts enhanced the synthesis of betulin, cholesterol, lanosterol, stigmasterol, and sitosterol. Another study also demonstrated that birch bark extract may stimulate the synthesis of steroids in Chaga, which have anti-tumor and anti-inflammatory properties (Debnath et al., 2013).

#### 6.4.3 Terpenoids

In the study by Peng and Shahidi (2022), a total of 108 terpenoids were identified in chaga. Seventy-eight of these terpenoids were newly detected. Apart from terpenoid compound T108, all terpenoids included substituent groups with multiple oxygens, non-oxygenated derivatives. Apart from sesquiterpenoids, monoterpenoids, diterpenoids, tetraterpenoids, pentaterpenoids, the remaining 103 terpenoids were steroids and triterpenoids. Chaga contains esters of triterpenoids including hydroxycinnamoyl indicating the presence of impure terpenoids. However, in contrast to phenolic compounds, the high molecular weight of terpenoid compounds makes classification by structure challenging due to the possibility of having several isomers (Peng and Shahidi, 2022).

## 7 Pharmacological potential

**FIGURE 6:**
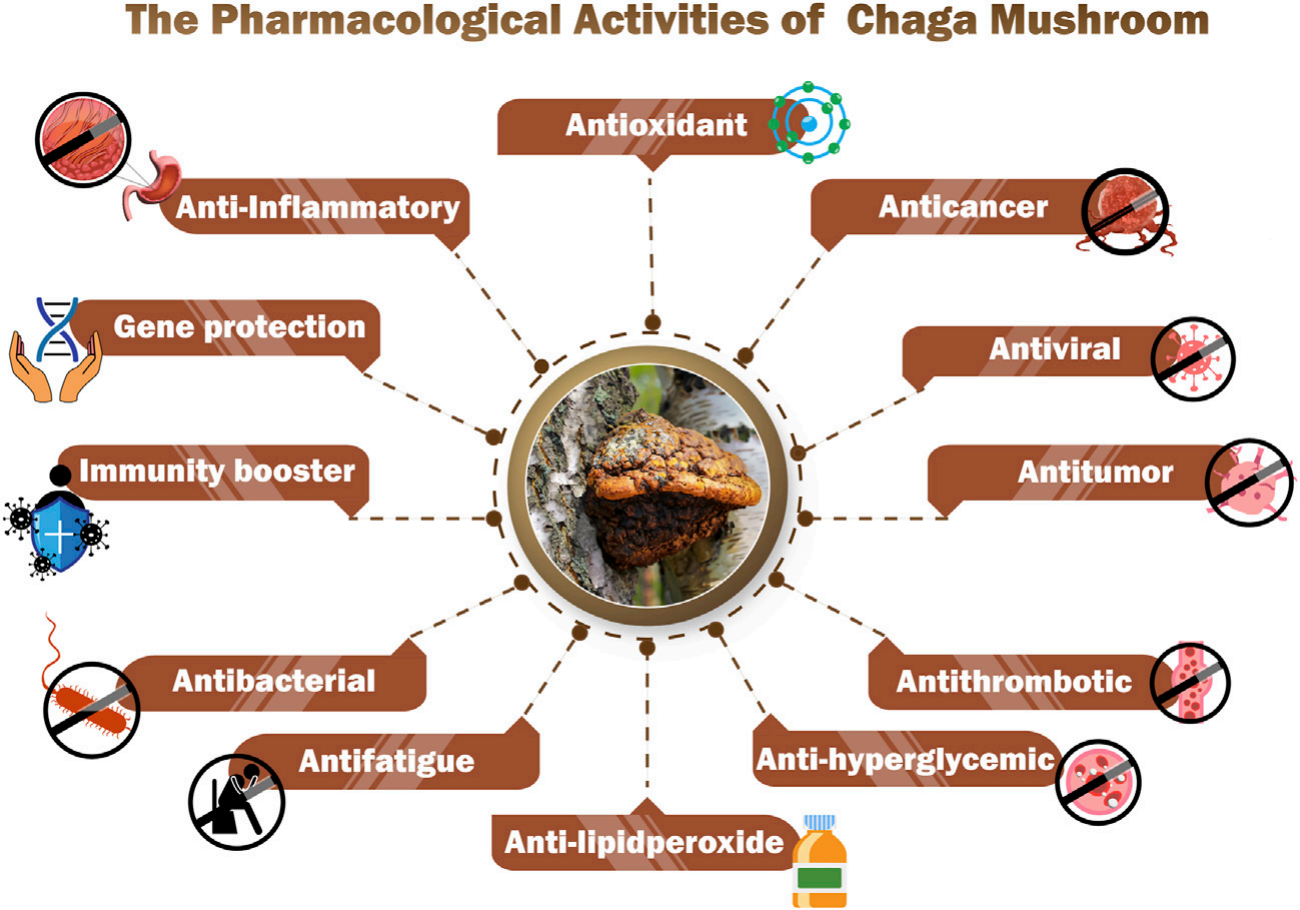
Pharmacological potentials of Chaga Mushroom.

According to several research studies (Figure 6), Chaga mushrooms possess around 130 pharmacological properties including anticancer, immunomodulating, gene-protecting, and antimicrobial capabilities (Chung et al., 2010; Thomas et al., 2020; Kou et al., 2021). The details of some pharmacological activities are discussed below.

### 7.1 Antioxidant properties

Chaga extract is widely recognized for its properties and multiple studies have shown its ability to counteract the effects of free radicals (Zheng et al., 2011b; Haines, 2013). In one study, to examine Chaga’s ability to operate as a superoxide dismutase (SOD) in scavenging radicals, the researchers utilized alcohol and three distinct hot-water extractions (each with a different temperature). According to Hu et al. (2009), their research revealed that the alcohol extract exhibited SOD activity, among all their investigations. On the hand, hot water extracts also showed SOD activity with higher temperatures resulting in stronger antioxidant abilities (Hu et al., 2009). Additionally, these researchers evaluated the four extracts’ capacity to scavenge 2,2 diphenyl 1 picrylhydrazyl (DPPH). Interestingly the alcohol extract displayed a higher DPPH scavenging ability as compared to hot water extracts (Hu et al., 2009).

Notably present in Chaga extracts is hispidin—a compound, with antioxidant and inflammatory properties. However, Chaga extracts’ composition can vary based on the source of the fungus, the extraction procedure, and the section extracted (Lee and Yun, 2006; Yusoo et al., 2002). Among the hispidin analogs are phelligridins, inoscavins, inonoblins, and davalialactone and its derivatives (Zheng et al., 2011b). These analogs contain the active ingredient that confers antioxidant action on the drug and is used to treat disorders associated with oxidative stress. In other studies, Giridharan et al. (2011) investigated the impact of the antioxidant activity of Chaga extract on the cognitive function of mice with amnesia. Researchers studied its effects on the brain and cognition after using the alkaloid scopolamine to induce cognitive impairment. In both tests, Chaga increased glutathione and superoxide dismutase levels (endogenous antioxidants), and improved learning and memory. The antioxidant properties of Chaga have gained recognition, for their ability to combat cancer promote heart health and help manage diabetes (Zhong et al., 2009; Song et al., 2013; Jiang et al., 2020). Moreover, findings demonstrate that Chaga extracts possess antioxidants that can effectively counteract radicals suggesting its potential in preventive and healing capacities, for various diseases (Mu et al., 2012). Song et al. (2004) examined NF-kB and antioxidant activity in malignant human keratinocytes (SCC-13) to determine whether pine bark extract, Chaga (*Inonotus obliquus*), and Chaga mycelium were effective. The activity of NF-BNF-B was downregulated in every substance that was evaluated using a cell-based NF-BNF-B monitoring assay. Using AGI-1120 (one to two mg) as the positive control and Chaga mushroom extract (0.05–0.1 mg), demonstrated a significant inhibitory effect on NF-BNF-B activity which implies that the Chaga mushroom extract was successful in scavenging DPPH radicals.

### 7.2 Anti-inflammatory properties

The extracts, from *Inonotus obliquus* known for their inflammatory properties, have gained popularity in countries like China, Korea, Japan, Russia, and the Baltics. When it was initially discovered in the mid-20th century it was utilized in the treatment of malignancies and digestive issues with no effects (Szychowski et al., 2021). Research has indicated that Chaga extract possesses inflammatory properties. Macrophages can release substances such, as nitric oxide, prostaglandin mediators, and pro-inflammatory cytokines (TNF α, IL 1 β IL 6) (Im et al., 2016). A study on the methanol and ethanol extracts of Chaga has shown that they inhibit macrophage activity by reducing the production of inflammatory mediators such as nitrogen oxides, prostaglandins (PGE2), and certain cytokines (Softa et al., 2019). According to a study conducted by Mishra et al. (2012), it was found that aqueous Chaga extracts have the potential to greatly alleviate effects caused by dextran sodium sulfate (DSS). These effects include reducing edema and mucosal damage and minimizing crypt loss. Additionally, the study revealed that Chaga extracts can effectively suppress the expression of inflammatory cytokines lower the levels of iNOS induced by DSS, and reduce the accumulation of myeloperoxidase in the colon (Mishra et al., 2012). The anti-inflammatory properties of Chaga associated with isolated ergosterol, ergosterol peroxide, and trametenolic acid were further confirmed by Kou et al. (2021). Ishfaq et al. (2022), demonstrated that aqueous extract of chaga possess anti-inflammatory chaacteristics. The study further demonstrated the ability of the extract to suppress expression of p53, caspace-3 and microcystin-LR known to cause inflammation and toxicity to the liver in mice (Ishfaq et al., 2022). In another study, Ishfaq et al. (2021), demonstrated that aqueous extract of chaga suppressed carbon tetrachloride (CCl_4_) induced damage in liver tissues in mice. The study also showed that ergosterol peroxide isolated from chaga has the ability to bind and inhibit activity of pro-inflammatory proteins (Ishfaq et al., 2021).

Betulin and betulinic acid have shown promising potential as antimicrobial and anti-inflammatory agents. With anti-inflammatory activities, these compounds are reported to modulate the activities of immune cells and prevent pro-inflammatory production (Oliveira-Costa et al., 2022). This, therefore, demonstrates its potential ability for the treatment of inflammatory ailments.

### 7.3 Anticancer and antitumor properties

Ma et al. (2013) evaluated the anticancer properties of chaga using bioassay-guided preparative isolation. Acetate and petroleum ether extracts significantly reduced NO generation and NF-kB luciferase activity in macrophage RAW 264.7 cells and induced cytotoxicity *in vitro* in human prostate cancer PC3 and breast cancer MDA-MB-231 cells. Inotodiol, ergosterol peroxide, and trametenolic acid were recovered from these two fractions. Both ergosterol peroxide and trametenolic acid were cytotoxic to the human prostatic carcinoma cell PC3 and the human breast cancer MDA-MB-231. These findings may help in understanding Chaga’s anti-cancer activity. Different cancers, including Walker 256 carcinosarcoma, MCF-7 human breast adenocarcinoma, sarcoma 180, and carcinoma 755, are sensitive to the effects of lanolin triterpenoids isolated from Chaga (Shin et al., 2000). Additionally, hot water extracts of Chaga were shown to have anticancer activity against human colon cancer cells HT-29. The main mechanisms involved in this process were increasing the levels of proteins that promote cell death and decreasing the levels of proteins that prevent cell death. This was shown in a study conducted by (Heo et al., 2017). Another study by Mizuno et al. (1999), found that purified endopolysaccharide from cultivated mycelia of Chaga suppressed tumor cell proliferation and activated B cells and macrophages. Moreover, Chaga mycelium, had an effect, on cdc25 phosphatase, an enzyme that regulates the cycle of cancer cells. In a study using Sepharose, researchers found that the drug contains a water-soluble polysaccharide (ISP2a), which showed antitumor activity *in vivo* and significantly enhanced the immune response in tumor mice. As an added benefit, ISP2a enhanced lymphocyte proliferation and increased the production of TNF-α (Liuping et al., 2012). Satoru et al. (2016) sought to explore whether Chaga water extract might be utilized as a long-term cancer therapy. Chaga water extracts were extremely anti-blastema active in the treatment of cancer. A study published in 2008 by Youn et al. (2008) indicated that Chaga water extract inhibits the growth of cancer cells *in vitro* and triggers apoptosis (programmed cell death, in which the cell breaks down into distinct apoptotic bodies) in various carcinoma cells.

Particularly betulinic acid has demonstrated promise in cancer research as it has demonstrated potential in shrinking cancer growth cells, inhibiting apoptosis, and shrinking tumors (Zhang et al., 2015). Other researchers, attest to its anticancer activity and demonstrate its selective antagonistic activity towards cancer cells (Saha et al., 2015; Kumar et al., 2018).

In 2015, a study examined the anti-cancer activity of ergosterol peroxide isolated from chaga on colon cancer cells. The study demonstrated that the compound increased apoptosis and suppressed cell growth in colon cell lines. The compound further inhibited the β-catenin signaling pathway, a major player in colon cancer development (Kang et al., 2015). Similarly, Mishra et al. (2013) found that aqueous extract of chaga expresses anti-proliferative and anti-inflammatory activity on colon cancer cells and tumors in mice. The study also demonstrated that extracts suppresses the Wnt/β-catenin and NF-κB signaling pathways that are responsible for colon cancer onset and development (Mishra et al., 2013).

### 7.4 Antiviral properties

For anti-viral properties, studies report the inhibitory properties of bioactive compounds in Chaga against a host of viruses including hepatitis C, HIV, and herpes simplex virus make them attractive candidates for antiviral therapies (Navid et al., 2014; Paduch and Kandefer-Szerszen, 2014; Xiao et al., 2018).

Satoru et al. (2016), shows that Chaga extracts have been found to impede the replication of both hepatitis C virus and human immunodeficiency virus (HIV). The intriguing potential benefits of Chaga, in treating diseases have captured the attention of researchers. Jin et al. (2017) investigated the antiviral effects of Chaga extracts on feline viruses. They proved that Chaga therapy was effective in cell assays and had minimal cytotoxicity by using feline calicivirus cell models. According to research on adhesion’s mode of action, the treatment of calicivirus causes an inhibitory effect on viral particles by preventing viral binding and absorption. Research has shown that cats may experience gastrointestinal issues due, to the herpes virus panleukopenia virus, and infectious peritonitis virus. These viruses have a range of activities. In this study, scientists discovered that Chaga polysaccharides could potentially serve as an antiviral treatment, for both feline and human pathogens. Moreover, researchers from Poland have shown in 1998 that botulin and betulinic acid found in Chaga can stop blastema development (Liang et al., 2009). They discovered that betulin and betulinic acid are now potential anti-HIV medicines that inhibit HIV reverse transcriptase, in turn inhibiting HIV type 1 (Gogineni et al., 2015). Human influenza A and B and horse influenza A are also inhibited by constituents of the black exterior surface of Chaga (Zheng et al., 2010). Betulin, mycosterol, and lupeol, which are all found in mushrooms, are thought to be the primary antiviral agents (Pan et al., 2013). Polkovnikova et al. (2014), examined the antiviral efficacy of Chaga extract on Vero cells using Vero cell cultures and HSV (herpes simplex virus) type 1 infected Vero cells. Most of the HSV-infected Vero cells were protected by non-toxic sub-components. Deletion of viral DNA in Chaga-infected cells suggests that it protects Vero cells from HSV cytotoxicity.

More recent research by Eid et al. (2021) has shed light on the impact of Chaga on the novel coronavirus (SARS-CoV-2) that has been responsible for widespread outbreaks around the globe. In this study, the S1-carboxy-terminal domain of the SARS-receptor-binding CoV-2 domain was discovered to be closely related to -glucan, galactomannan, and betulinic acid. At the TRP-436, ASN-437, and ASN-440 sites, this interaction was substantial. The receptor binding domain of the most recent SARS-CoV-2 isolates had a furin cleavage site that was not seen in prior isolates. Since this response interacted with ACE-2 more often, this virus was able to infect more people. Chaga, according to those who took part in the research, might be used in conjunction with other medications to combat SARS-CoV-2. Hence, future anti-SARS-CoV-2 therapeutic development may be aided by the development of naturally derived anti-coronavirus therapies that include Chaga (Eid et al., 2021).

### 7.5 Antithrombotic activity

Platelet aggregation is a complicated event that is most likely the product of several metabolic pathways working in concert. The use of platelet inhibition in the prevention of thrombosis is a potential new treatment. According to a report by Hyun et al. (2006), water and ethanol extracts from 55 different kinds of mushroom mycelium or fruiting bodies were tested for platelet aggregation inhibitory activities *in vitro*. The ethanol extracts of Chaga ASI 74006 mycelia demonstrated the highest platelet aggregation inhibitory activity (71.2%), whereas the impacts of extracts from its fruiting bodies had very low inhibitory activity. The isolated peptide was found to have a strong inhibitory activity of 91.6% and a low molecular mass of 365 Da. It is assumed that the peptide from Chaga which inhibits platelet aggregation is quickly absorbed in the gut, and is commonly employed in the creation of antithrombotic medications and nutritional supplements for human consumption (Huang et al., 2012).

### 7.6 Other therapeutic uses

#### 7.6.1 Effects of chaga on diabetes

Researchers have found that Chaga mushroom extract can lower blood sugar (Rogers, 2012). The effects of Chaga on diabetes were evaluated by Xu et al. (2010). Mice with diabetes were given an extract of the polysaccharide fraction of Chaga, which reduced maleic dialdehyde activity. After histological morphological examination, the study revealed that the Chaga extract significantly reduced damage to their injured pancreatic tissues (Xu et al., 2011). The polysaccharides derived from Chaga have shown properties in combating high blood sugar levels and lipid oxidation (Xu et al., 2011; Hu et al., 2012). Another study investigated the chemical composition and blood sugar-lowering effects of the ethyl acetate fraction extracted from Chaga revealing its hyperglycemic and anti-lipid peroxidative actions, in mice with diabetes induced by alloxan (Lu et al., 2010). Postprandial hyperglycemia is closely associated with type 2 diabetes mellitus and its complications. The research demonstrated that an acidic protein-bound polysaccharide called IOPS derived from Chaga effectively inhibits the activity of β glucosidase at a concentration of 93.3 g/mL. Additionally, Chen et al. (2010) discovered that it also suppresses stress and the generation of compounds reactive to acid during Fe2+/ascorbate induced lipid peroxidation in rat livers (Chen et al., 2010). Exploring substances as inhibitors of β glucosidase could potentially lead to the development of foods or promising compounds, for combating diabetes (Chen et al., 2010). These findings have the potential to contribute to the development of safe inhibitors of β glucosidase. These inhibitors can be derived from sources making them a promising choice, for creating foods or lead compounds in anti diabetic treatment.

#### 7.6.2 Chaga in the prevention of DNA damage

In a study by Kyoung et al. (2004), human lymphocytes were subjected to oxidative DNA damage, and an aqueous extract of Chaga was tested for its protective effect. One-cell electrophoresis for DNA fragmentation was used to detect oxidative damage in the test subjects’ bodies (comet analysis). The DNA fragmentation in cells that were pre-treated with Chaga extract was reduced by more than 40% compared to cells that had been treated with the positive control (100 micron/mole H_2_O_2_).

It has been shown that *Inonotus obliquus* polysaccharides (IOPS) may boost the immune system and reduce oxidative stress throughout the growth process (Youn et al., 2008). However, more studies are required to understand the impact of IOP, on genotoxicity in model organisms. Liuping et al. (2012) conducted experiments where they exposed embryos (12 h post fertilization) to UVB radiation (12 J/m_2_/s, 310 nm) for 10 s and then administered IOP therapy (2.5 mg/L) after 24 h post fertilization continuing for a duration of, up to 7 days (Liuping et al., 2012). Crimson, orange staining, the alkaline comet test, and qRT-PCR screening of DNA repair genes were used to evaluate genotoxic effects. The IOP-treated zebrafish were exposed to UVB at 5 days post-fertilization and subsequently exhibited a substantial decrease in DNA damage and improvement of the distorted structures. The relative mRNA expressions of RAD51, P53, and GADD45 dramatically increased in IOP-treated UVB-exposed zebrafish. DNA repair genes were shown to be coordinated in their response to UVB exposure, indicating a communal response. Finally, the IOP therapy reduced UVB-induced genotoxic effects on zebrafish embryos, allowing them to grow normally (Liuping et al., 2012).

Because of the lack of information on probable interactions between various dietary supplements, there is rising concern about negative effects. For the first time, Živković et al. (2019) investigated the genotoxic effects of Chaga and dihydroquercetin in combination. When Chaga (250 g/mL) and dihydroquercetin at 100 g/mL, 250 g/mL, and 500 g/mL were administered alone or together, no genotoxic impact was found on whole blood cells. The comet test was used to investigate the antigenot1oxic effectiveness against DNA damage caused by hydrogen peroxide (H_2_O_2_) in whole blood cells. Dihydroquercetin alone did not affect H_2_O_2_-induced DNA damage in cells, whereas the combination of Chaga and 500 g/mL dihydroquercetin was most effective. Hydrogen peroxide-induced oxidative damage to genomic material may be prevented *in vitro* by taking Chaga and dihydroquercetin together. Table 3 is the summary of the established literature report on the pharmacological potentials of Chaga mushrooms.

**TABLE 3:** Established literature reports on pharmacological potential of Chaga mushroom.

S/N	Type of extract	Type of assay	Pharmacological activity	Major findings	Country of study	References
1	Methanolic extract (MeOH)	*In vitro*	Anticancer	These results support the use of *Inonotus obliquus* in treating lung cancer and disclose the molecular basis for its cytotoxic effect against human lung cancer cells	South Korea	Baek et al. (2018)
2	Ethyl acetate extract	*In vitro*	Antimutagenic and Antioxidant	According to the findings, three of the components of Chaga, -3-hydroxy lanosta-8, 24-dien-21-al, and inotodiolal — have antimutagenic and antioxidative properties, respectively	South Korea	Ham et al. (2009)
3	Chaga mushroom powder	*In vivo*	Anticancer	The results showed that oxalate crystals were detected in the renal tubular lumen and urinary sediment, and oxalate nephropathy was diagnosed. Kidney function declined and hemodialysis was started. This response is the first report of a case of this kind related to the ingestion of Chaga mushrooms	Japan	Kikuchi et al. (2014)
4	Hot water extract (IOE)	*In vitro Ex vivo*	Immunomodulating effects of Chaga	The results show that Chaga regulates antigen-specific antibody production and release of Th1/Th2 cytokines in immune cells	South Korea	Ko et al. (2011)
5	Hot water extract	*In vitro*	Antitumor	Hot water extracts of Chaga (IOWE) can be used to treat cancer by increasing the expression of pro-apoptotic proteins while decreasing the expression of anti-apoptotic proteins, thereby helping cancer cells die	South Korea	Lee et al. (2009)
6	Aqueous extract (IOAE)	*In vitro*	Anti-inflammatory	IOAE suppresses TNF-α, iNOS and IL-1, indicating that it may be useful in preventing inflammatory bowel illness	South Korea	Mishra et al. (2012)
7	Ethanolic extract	*In vitro*	Prevents inflammatory bowel disease (IBD)	Chaga extract decreases oxidative stress in lymphocytes from IBD patients and healthy people	United Kingdom	Najafzadeh et al. (2007)
8	Polysaccharide extract (IOPE)	*In vitro*	Antitumor	A Chaga extract was found to inhibit tumour cell growth	China	Ning et al. (2014)
9	Aqueous extract (AEIO)	*In vitro*	Antivirus	Herpes simplex virus (HSV) infection was significantly reduced when *I. obliquus* aqueous extract (AEIO) was used	China	Pan et al. (2013)
10	Methanol extract (MEIO)	*In vitro* and *In vivo*	Anti-inflammatory	In LPS-stimulated RAW 264.7 macrophages, Chaga dramatically reduced NO, PGE2, and TNF-α production	South Korea	Park et al. (2005)
			Anti-nociceptive			
11	Dry matter of culture broth (DMCB) of *Inonotus obliquus*	*In vitro*	Antihyperglycemic	DMCB of *Inonotus obliquus* had strong antihyperglycemic, antilipidperoxidative, and antioxidant properties in alloxan-induced diabetic mice	China	Sun et al. (2008)
			Antilipidperoxidative			
12	Polysaccharide extract (IOPE)	*In vitro*	Antifatigue	IOPE increased swimming time in mice but decreased lactic acid and urea levels. Studies on major organs, including the liver, showed no negative consequences of IOP. These findings support the use of IOP as an anti-fatigue medication	China	Xiuhong et al. (2015)
13	Polysaccharides extracted from Chaga	*In vitro*	Antihyperglycemic	In alloxan-induced diabetic mice, Chaga DMCB polysaccharide extract lowered blood glucose levels	China	Xu et al. (2010)
			Antilipidperoxidative			
14	Aqueous extract (IOAE)	*In vitro*	Anticancer	Extract reduced cell growth, causing G0/G1-phase arrest and apoptotic cell death	South Korea	Youn et al. (2008)
15	Aqueous extract (IOAE)	*In vitro*	Anticancer	The *Inonotus obliquus* aqueous extract significantly reduced tumour growth in mice. The *Inonotus obliquus* extract may be utilised as a natural cancer suppressant by improving energy metabolism	South Korea	Arata et al. (2016)
16	Aqueous dried fruiting body Chaga fraction	*In vitro*	Anticancer	The results suggest that Chaga has anticancer benefits that are partially attributed to reduction in tumor cell proliferation, motility, and morphological changes	Poland	Lemieszek et al. (2011)
17	Polyphenolic and Polysaccharide extract (IOPE)	*In vitro*	Antioxidant	The results of the study showed that IOPE protected cells from oxidative stress and scavenged free radicals at concentrations above 5 g/mL	South Korea	Cui et al. (2005)
18	Aqueous extract (IOAE)	*In vitro*	Immunity enhancer	The extract increased blood IL-6 levels when administered orally. Reduced TNF-related pathological abnormalities were observed in the extract-treated animals. These findings support the use of IOAE as an immunity enhancer during chemotherapy	South Korea	Kim (2005)
19	Aqueous extract (IOAE)	*In vitro*	Anticancer	IOAE at 10 μg/mL to 2000 μg/mL concentration suppressed cancer cell development	Poland	Burczyk et al. (1996)
20	Aqueous extract (IOAE)	*In vitro*	Anticancer	French Chaga has cytotoxic effects on normally transformed BEAS-2B cells	France	Géry et al. (2018)
21	Gold nanoparticles (AuNPs) synthesized using *Inonotus obliquus*	*In vitro*	Antibacterial Anticancer	Both the MCF-1 human breast cancer cell line and the NCI-N87 human stomach cancer cell line demonstrated high antibacterial, antioxidant, and cytotoxic activity	South Korea	Lee et al. (2015)
22	Ethanol and water extracts	*In vitro*	Immunity booster	The findings show that the Chaga mushroom can make a small-molecule inhibitor that stops the CTLA-4/CD80 interaction. They believe that Chaga can be used to create a new type of immune checkpoint inhibitor	South Korea	Kim et al. (2020)

## 8 Cosmetic uses

Melanin, a pigment, in the skin plays a vital role in shielding the skin from harmful UV radiation (Kukulyanskaya et al., 2002; Tsatmali et al., 2002). However, when exposed to UV light and other triggers the skin naturally produces melanin as a protective response (Yan et al., 2014). In other studies, two naturally occurring chemicals, birch sap from *Betula alba* and organic extracts from Chaga, were investigated for their potential to block the Sun’s ultraviolet (UV) rays (Alhallaf, 2020). Both pure birch sap and Chaga extracts, as well as a combination containing 5% organic birch sap and 5% Chaga, were tested.

The birch tree sap derived from Betula alba and Chaga proved effective, in reducing the production of cytokines when the skin was exposed to UV light. Keratinocytes are shielded from the DNA damage caused by UV light by extracts of birch sap and Chaga mushrooms. These findings demonstrate for the very first time that extracts of Chaga and Betula alba mushrooms shield skin cells from the damaging effects of UVA and UVB radiation because of their antioxidant, anti-inflammatory, and DNA-preserving and repairing properties. Moreover, they have the potential to be used in anti-aging and sun-screening cosmetics (Softa et al., 2019).

**FIGURE 7:**
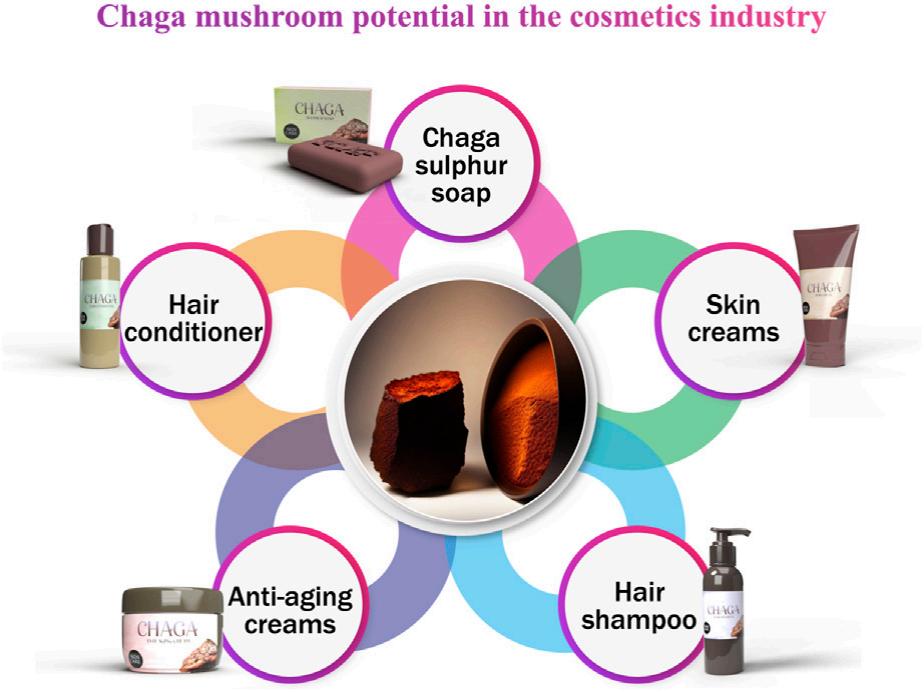
Cosmetics uses of Chaga mushroom.

Based on a study’s findings, Song et al. (2004) concluded that Chaga and Chaga mycelium may inhibit NF-BNF-B in human skin (Figure 7). Chaga possesses antioxidants, like melanin and hispidin analogues (polyphenols) along with dismutase and catalase. This makes it a promising ingredient in the field of dermatology for addressing signs of aging such as wrinkles, sagging skin, and graying hair. Oxidative stress is believed to be a contributing factor to these manifestations of aging. Excessive free radicals resulting from sunlight exposure and other oxidative damage can lead to accelerated skin deterioration due, to the body’s inability to effectively neutralize them (Song et al., 2013). More antioxidants in the body might theoretically delay or even halt the aging process. The efficiency of Chaga in combating other kinds of oxidative stress implies that it may also combat aging, although no clear study has connected it to anti-aging effects (Géry et al., 2018).

Chaga sulphur soap is manufactured in South Korea, where it is said to have anti-aging and hydrating effects, among others (Ţura et al., 2018). Sagayama et al. (2019) conducted cell proliferation experiments to assess the impacts of Mongolians using Chaga sclerotium to wash their hair traditionally. They isolated five lanostane-type triterpenes (1–5) from human hair follicle dermal papilla cells (HFDPCs). These triterpenes outperformed the positive control, minoxidil, for their pro-proliferative effects on HFDPCs.

Yan et al. (2014) investigated the potential of Chaga to reduce melanin pigmentation and explore its use as a skin whitening ingredient in cosmetics. Specifically, they focused on tyrosinase, an enzyme, for stimulating production. It was discovered that the tyrosinase enzyme was inhibited by betulin and trametenolic acid, but other compounds such as inotodiol and lanosterol activated tyrosinase and increase pigment synthesis in laboratory cells. Pigment reduction may assist people with darker patches to achieve a more even skin tone, whilst pigment activation may be beneficial for those who have lost or decreased their pigment. It seems that Chaga has great potential as a cosmetic agent, and further studies are needed to determine how beneficial the various components may be for the cosmeceutical industry.

### 8.1 Industrial applications

Chaga produces ligninolytic enzymes that act as biocatalysts in oxidoreductase processes such as degrading lignin and other parts of the cell wall (Dashtban et al., 2010). Particularly, ligninolytic peroxidase degrades polymers and other complex components in an oxidative process, splitting the entire lignin structure (Silvat, 2013). In a similar fashion, the enzyme degrades polysaccharide compounds in hemicellulose and cellulose. By breaking down these materials, Chaga can access the nutrients present in the wood and utilize them for its growth and survival (Cajthaml and Svobodová, 2012).

This ability of Chaga’s enzymes to degrade lignin and other woody materials has attracted interest for potential applications in various industries. One such application is bioremediation, where these enzymes play a role in the cleanup of polluted environments. By breaking down lignin and other organic pollutants, such as pesticides, dyes, and industrial chemicals, these enzymes contribute to the degradation of toxic compounds, leading to the remediation of contaminated sites and a reduction in environmental pollution (Singh et al., 2021). Another notable application lies in biofuel production. In one study, lignocellulosic biomass, including wood and agricultural residues, was reported to be beneficial as a feedstock for biofuel production. Ligninolytic peroxidase enzyme hydrolyzed the lignocellulosic materials, breaking them down into simpler sugars. These sugars were then fermented to produce biofuels like ethanol. By facilitating the degradation of biomass, these enzymes present a promising avenue for the advancement of biofuel technologies (Ilić et al., 2023).

In the paper and pulp industry, these enzymes have been reported to provide a more environmentally friendly alternative to the conventional pulping and bleaching processes. The enzymes activate the bond between ink and paper particles through hydrolysis which is subsequently removed via a flotation technique (Lee et al., 2013).

Traditionally, these industries rely on harsh chemicals and energy-intensive methods (Kaur et al., 2020). However, the enzyme was found to selectively degrade lignin, increasing the efficiency of the pulping process while reducing the dependence on chemicals. Furthermore, these enzymes can be employed in the bleaching process, minimizing the use of chlorine-based bleaching agents that pose harm to the environment (Singh et al., 2021).

Chaga enzymes have also shown potential in the textile and detergent industries. As bio-catalysts, they can be utilized in the processing of natural fibers like cotton and hemp (Pavithra et al., 2023; Asemoloye et al., 2021 reported that by effectively removing lignin and impurities from these fibers, the ligninolytic peroxidase enzyme contributed to the production of cleaner and higher-quality textiles. In the detergent industry, Chaga enzymes were found to enhance the efficiency of laundry detergents by aiding in stain removal and facilitating the degradation of organic matter (Asemoloye et al., 2021).

## 9 Agricultural potentials

**FIGURE 8:**
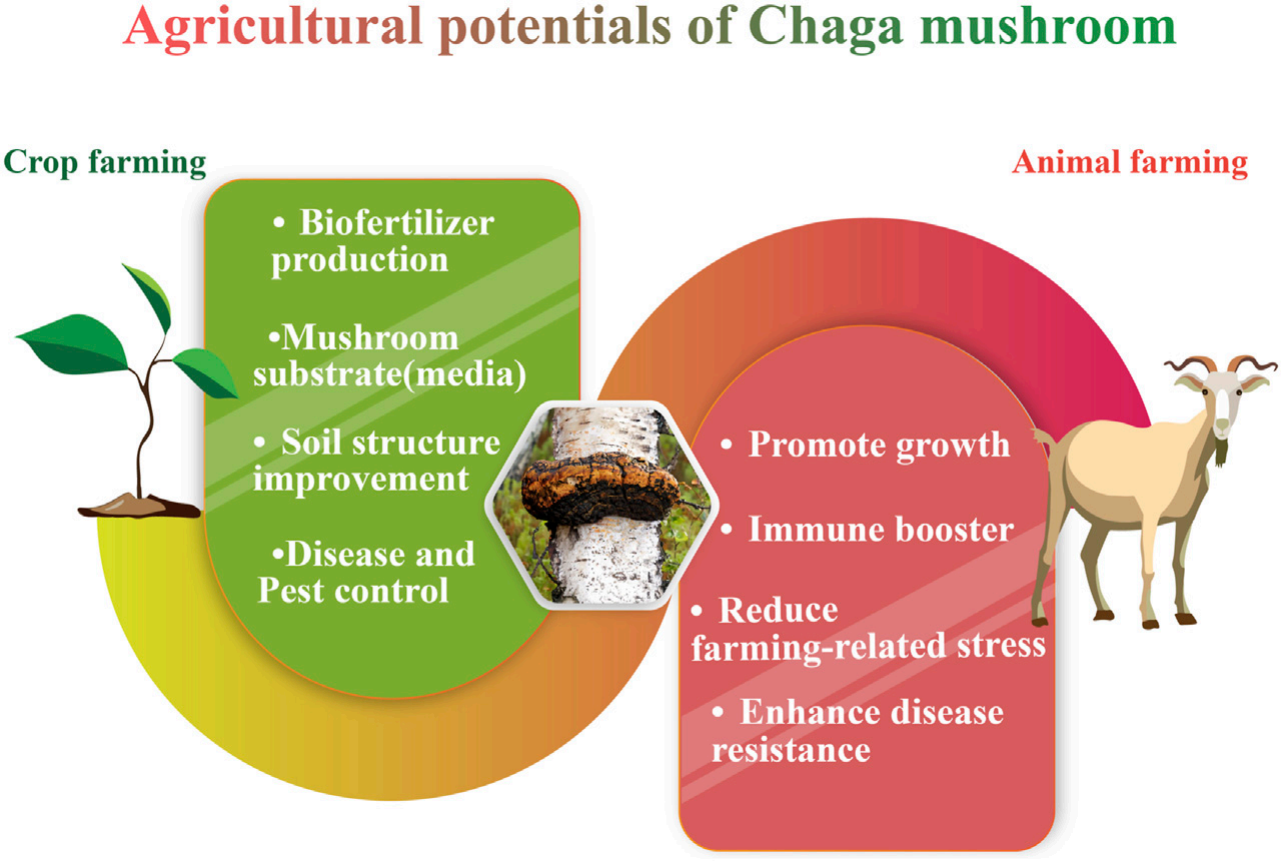
Agricultural potentials of Chaga.

Aside from the pharmacological advantages of Chaga, there have been a few discoveries regarding its use in crop and animal husbandry. However, despite Chaga’s popularity and potential for use in agriculture, this application of the fungus has not been subjected to a rigorous scientific review. Hence, this part of the review will focus on Chaga and its prospective uses in agriculture (Figure 8).

### 9.1 Potentials of chaga in animal farming

The use of these mushrooms as lignocellulosic materials were conceptualized more than a century ago by Falck (1902), who proposed their use for improving agricultural waste and subsequently animal nutrition. After almost a century, several newer studies also focus on improving lignocellulosic materials for fodder (Lindenfelser et al., 1979; Hadar and Cohen-Arazi, 1986). This includes investigating the underlying biochemical mechanisms of the fungal degradation of agricultural waste. Studies have also considered the lignocellulose complex in straw and plant residues, and the reaction mechanisms connecting lignin polymers and hydrolytic enzymes such as cellulases and hemicelluloses (Hadar et al., 1992; Honda et al., 2000).

Antibiotics have become a major aspect of livestock production, especially in developed countries (Rathgeber et al., 2008). The initial evidence, reported by Moore et al. (1946) showed the benefits of administering doses of antibiotics to chickens (Moore et al., 1946). Their study indicated that the inclusion of streptomycin improved the growth rate of the chicks (Moore et al., 1946). Chaga mushrooms contain a concentration (8.57%) of *β* glucan which is recognized for its immune system support properties and has been shown to improve the growth rate of fowls (Chandrasekaran et al., 2011). Currently, few studies conducted to confirm that incorporating β glucan, into animal feed has an overall impact, on the system and the growth of livestock.

Several studies have shown that the addition of *β*-glucan to animal feed positively affects the immune system and the growth of farm animals. One study, in particular, demonstrated that broiler feed supplemented with *β* glucan increased the weight of the broilers significantly as compared to broilers given feed without supplemented *β* glucan (Chae et al., 2006). Rathgeber et al. (2008) conducted studies that revealed β glucan to be equally effective, as virginiamycin, in promoting the growth of broiler chickens. Similarly, Dritz et al. (1995) demonstrated that the addition of β glucan improved the growth of nursery pigs while incorporating Chaga into their diet enhanced piglet growth and facilitated weight gain. Not are β glucans utilized in the food and pharmaceutical industries as anticancer and immune-modulating agents. They are also employed within the aquaculture and livestock sectors to enhance animals’ innate immunity (Jung et al., 2007; Zhu et al., 2016). According to (Jung et al., 2007), β-glucan from *Paenibacillus polymyxa* JB115 can be added to animal feed to enhance immunity and may act as an anticancer agent in livestock. Additionally, rainbow trout (*Oncorhynchus mykiss*) were fed β-glucan pellets, increasing the secretion of specific antibodies and Ig levels in serum (Skov et al., 2012). Owing to the emergence of new diseases and government efforts to prohibit antibiotics that promote growth while enhancing the efficacy of commercial agriculture, new opportunities for natural, highly effective, and inexpensive immunomodulators like Chaga mushrooms can compensate by inducing and enhancing disease resistance while reducing losses. Moreover, these mushrooms produce significant quantities of β-glucan that can be used in feed supplements (Cueno et al., 2004). These supplements can improve immunity, reducing the excessive use of antibiotics. This has been confirmed by the immunostimulation of fowl after being fed with *Pleurotus ostreatus*, which considerably improved without affecting the size or quality of the meat (Li et al., 2006; Zhang et al., 2008).

In addition, these medicinal mushrooms have been investigated extensively as prospective materials for improving the quality of agricultural lignocellulose waste (Bogan et al., 1999). In one study, Chaga mushroom helped to breakdown lignin, releasing cellulose that can be used by ruminants (Kamra and Zadražil, 1986). This breakdown mechanism was linked to the ligninolytic system of the mushroom made up of oxidative enzymes. In another study, the direct use of mushrooms as lignocellulosic residues in animal feed played an important role in the diet of ruminants (Streeter et al., 1982). Studies have also examined the use of *Pleurotus spp* in breaking down wheat straw under different conditions and substrate treatments (Zadrazil, 1997; Salmones et al., 2005; Khan et al., 2013; Muswati et al., 2021). A significant increase in digestibility of wheat straw was observed after treatment with the mushroom. Similarly, Hadar et al. (1992) found that the lignin constituents in wheat straw significantly decreased during 4 weeks of treatment with *P. ostreatus*. *In vitro* digestion also increased and the product was degraded by up to 40% in animals’ diets (Hadar et al., 1992).

A major barrier to the upgrade of straw to fibre is the expensive materials and equipment needed for the preparation of fungal substrates (Hüttermann et al., 2000). Hüttermann et al. (2000) described a new process for degrading agricultural wastes using *Pleurotus* spp. This included using solar heating for pasteurization, and treatment using detergents, tomato pomace, and potato pulp. These methods generated positive results in on-farm applications (Hüttermann et al., 2000). Moreover, in both *in vitro* and *in vivo* studies, *Pleurotus* spp. significantly improved the availability of roughage in animal diets due to its action on cellulose and lignin (Van Kuijk et al., 2015). In a feeding experiment by Hüttermann et al. (2000), rams fed with mushroom-treated straw increased in body weight. At the same time, the treated group demonstrated an increase in nutritive value (Hüttermann et al., 2000).

In a study by Nwafor et al. (2022), a significant increase in the mineral composition of animal feed produced from agro -waste was observed after treatment with extracts of mushrooms. Its amino acid and carbohydrate contents also increased significantly as compared to the positive control, confirming the potential for mushrooms to improve the nutritional composition of animal feed (Nwafor et al., 2022).

### 9.2 Potentials of the chaga mushroom in crop farming

Boulet and Bussières (2018) mentioned that an arborist in Quebec employed Chaga powder and paste to treat beech tree blight caused by *Cryphonectria parasitica*. The incision healed over 2 years, and the trees became immune to blight. This means that Chaga might be used as an inoculation or crop-protection strategy for other tree species. Chaga is also used as a fertilizer to protect cultivated plants from Phytophthora, and as a plant growth stimulant to promote development (Shashkina et al., 2006). Some Russian researchers, especially P. A. Yakimov and others (Bulatov et al., 1959; Shashkina et al., 2006; Sysoeva et al., 2012), conducted detailed investigations of Chaga and its concentrated extracts along with those of other porous fungi about a century ago (Bulatov et al., 1959). The chemical composition of Chaga is significantly different from other polypores. An X-ray fluorescence approach, atomic absorption spectrum, and gravimetric analysis demonstrated that Chaga contains the following: carbon (39%), potassium (9%–10%), hydrogen (3.6%), nitrogen (0.4%), magnesium (0.64%), calcium (0.37%), chlorine (0.33%), phosphorus (0.23%), sodium (0.04%). The nitrogen content of Chaga is primarily part of proteins. The products of hydrolysis exposed 15 different amino acids, the most prevalent of which were glycine, aspartic, and glutamic acids (accounting for approximately 40%), as well as tyrosine, serine, threonine, leucine, methionine, lysine, and histidine (Shashkina et al., 2006).

*Invitro* studies have shown these mushrooms to be useful in the degradation of industrial dyes, phenols, soil decontamination, and wastewater treatment. Yet, no further analysis such as *in situ* tests has been done to further confirm these claims (Irie et al., 2001). Roy et al. (2015) investigated the improvement of plant health for *Capsicum annuum* L by treating it with spent mushroom substrate from button and oyster mushrooms. After several weeks of treatment, growth parameters such as height, branches, and yield improved significantly (Roy et al., 2015; Somnath et al., 2015). Apart from that, the substrate played a role in reducing soil phosphate and increasing root and leaf phosphate. The c9hlorophyll and carotenoid content of the leaves also significantly increased following soil treatment with the mushroom substrate (Roy et al., 2015).

Currently, mushroom substrates are recognized as inexpensive, ecologically friendly sources of organic fertilizers. Features such as high moisture, nutrient retention, high air permeability, loose texture, and rich pellet structure are known to improve soil structure and maintain a beneficial environment for soil microorganisms. Among the numerous applications of mushrooms to crop farming, the use of spent mushroom substrate remains the most effective and economical (Lou et al., 2017). The spent substrate, from mushrooms contains an amount of matter and essential nutrients like nitrogen, phosphorus, and potassium. These elements play a role in promoting plant growth. Through the utilization of mushroom substrate, as a soil amendment, farmers can enhance the quality and fertility of their soil without having to rely on synthetic fertilizers (Esmaielpour et al., 2017). Secondly, the use of spent mushroom substrate can help to reduce waste and save resources. After the mushrooms are harvested, the remaining substrate can be recycled and used as a soil amendment instead of being disposed of as waste (Cunha Zied et al., 2020). Third, the production of spent mushroom substrate is relatively low-cost compared to other types of organic amendments, such as compost or manure. This is because the substrate is already produced as a byproduct of mushroom cultivation, so there are no additional costs associated with its production (Meng et al., 2018).

Another study demonstrated that treatment with beneficial microorganisms improved the biological and nutritional activity of the substrate thereby improving soil structure, efficiency, quality, and ecological function (Mohd Hanafi et al., 2018). In another study, after composting, elements such as phosphorus, potassium, and nitrogen increased after treatment with the mushroom (Akdeniz, 2019; Sánchez, 2010). Moreover, during the composting process, mushroom substrates diminished the function of pathogenic microorganisms and by extension plant diseases, while supplying these essential nutrients.

Other studies have demonstrated the benefits of combining mushroom substrates with organic waste or sludge (Meng et al., 2018; Meng et al., 2019). In another study, substrate-compost mixes improved nitrogen content in plants by more slowly releasing nitrogen from the substrate, increasing overall uptake (Uzun, 2004). Grimm and Wosten (2018) also illustrated that mushroom substrates can be used to replace chemical fertilizers.

Field experiments by Courtney and Mullen (2008) compared a chemical fertilizer to an organic mushroom substrate. The application of mushroom substrate improved soil properties and the yield of barley by 50% relative to the 40% improvement from the chemical fertilizer (Courtney and Mullen, 2008). This improved performance was attributed to the increase of mineral contents by 3 times for the mushroom substrate, the chemical fertilizer yielded no increase. As mentioned earlier medicinal mushrooms like Chaga contain compounds such as polysaccharides, polyphenols, steroids, and terpenoids that have antibacterial and antioxidant properties (Glamočlija et al., 2015). Moreover, the application of mushroom-spent substrate to soils slowly releases these bioactive compounds into the soil. This can potentially benefit plants by acting as a defense against diseases. For example, a research study demonstrated that using this type of biofertilizer led to plant growth and increased resistance against the fungal pathogen Fusarium oxysporum (Awad-Allah et al., 2022). In another study focused on chili pepper plants affected by root rot disease scientists explored the potential of a biofertilizer derived from *Bacillus subtilis* bacteria. The findings revealed that applying this biofertilizer significantly reduced both disease incidence and severity while also promoting plant growth and higher yields (Sivasakthi et al., 2014). Similarly in a study involving tomato plants infected with Rhizoctonia solani fungus researchers investigated the effects of a biofertilizer derived from Trichoderma fungus, on growth and disease resistance levels (Sivasakthi et al., 2014). According to Awad-Allah et al. (2022) when the biofertilizer was applied it led to plant growth. Increased protection, against the pathogen (Awad-Allah et al., 2022). This claim is further supported by Othman et al. (2020) research, which showed that mushroom substrates have properties that can combat plant pathogens. Apart from agriculture, the antibacterial effect of these mushrooms has also been used to suppress food and clinical pathogens (Hearst et al., 2009).

## 10 Chaga safety and side effects

Since antiquity, the use of Chaga and its crude extracts has been completely harmless. Although the useful bioactivities of Chaga have attracted a lot of attention in the last decade, the safety and toxicology of the fungus has not been extensively investigated. Moreover, few studies that have investigated the safety and toxicology present divergent views on the safety of the mushroom.

In one study, researchers claim that the fungus is safe for consumption, whether ingested by animals or plants. However, Cui et al., in their study showed that the ethanol extract of Chaga was found to be harmful to HaCaT keratinocytes cells when applied in concentrations ranging from 100 to 400 g/mL (Cui et al., 2005).

Another study has shown that the primary porcine hepatocytes (PLP2) were not significantly affected by alcohol-based and/or aqueous extracts of Chaga at a concentration of 400 ppm compared to those exposed to alcohol for the first time (Glamočlija et al., 2015). Furthermore, other studies and numerous investigations have concluded that the use of Chaga extract does not result in any impact, on weight fluctuations, or effects on liver and kidney functions in kumming mice and Sprague Dawley rats (Kim et al., 2006; Nakata et al., 2007; Yong et al., 2018). Yet varying concentrations of oxalic acid, a toxic substance found in the fungus are documented in several studies (Kikuchi et al., 2014; Glamočlija et al., 2015; Lee et al., 2020). Although the understanding of the exact mechanism between the concentration of oxalic acid and its excretion ability is unclear, it is possible that with time extended use of chaga in any form may lead to increased levels of oxalate in the blood. This increase in levels may pose a risk, for sensitive individuals and potentially lead to oxalate necrosis.

The overuse of natural fungi such as Chaga is a growing concern among medical experts around the world (Lumlertgul et al., 2018). It is currently widely accepted that conducting trials on every fungus worldwide is nearly impractical due to the high cost and workforce required. Moreover, the nutritional host largely influences the chemical composition and safety of mushroom. However, following strict safety and health regulations can safeguard the safety and efficacy of Chaga products. Moreover, more toxicology studies on chaga products are needed in the present literature to authenticate the safety of these products before their use in treating illnesses.

## 11 Conclusion and prospects

*Inonotus obliquus* (Chaga mushroom), just like other medicinal fungi has been acknowledged for its healing abilities in traditional, folk, and modern medicine. In the current review, the fungus has demonstrated its wide array of medicinal properties including antimicrobial, anti-inflammatory, anticancer, antioxidant, and anti-tumor evaluated *in vitro* and *in vivo*. Yet, current evidence on its biological activity is still to a large extent scanty. Although studies have established the benefits of Chaga’s bioactive properties, more rigorous research is needed to fully understand the fungus’s mechanisms of action and to harness its full potential for future use in modern medicine.

Extraction of bioactive components using water was found to be the most common technique of preparation (or as a decoction) by many researchers and indigenous people. Moreover, most claims about Chaga’s traditional medicinal properties have also been proven true, either by *in vitro* or *in vivo* testing. Further clinical trials and translational studies are however required to further acquire more information on the fungus that will serve as a basis for future research. This could provide the necessary data to support the development of innovative medicines or applications as well as present alternative use for this mushroom. Eventually, this could pave the way for harnessing the bioactive characteristics of Chaga for various treatments.

Most research on Chaga was conducted in Asia and North America, with no available literature related to Africa. This is understandable because of its preference for certain climatic conditions (e.g., cold-loving), its origins in Asia (particularly Russia), and its extensive distribution across Europe and North America. Despite the mushroom’s widespread availability and traditional uses, it may nevertheless be considered underutilized since its full potential has not yet been explored (for example, by confirming its use as a traditional medicine for diseases other than originally used, or by further applying the confirmed *in vitro* properties of pharmacology to *in vivo* research and human or clinical trials for drug development).

Furthermore, Chaga mushrooms have shown promise in agricultural applications. In animal farming, researchers have studied Chaga and its bioactive compound β glucan for their potential to stimulate the system and promote growth in livestock. Adding Chaga or β glucan to animal feed has been found to boost immunity enhance growth performance and lessen the reliance on antibiotics. Moreover, scientists have explored the use of Chaga mushrooms to enhance the quality and digestibility of waste which can serve as feed, for ruminant animals. Utilizing Chaga in animal farming holds the potential, for improving animal wellbeing, and productivity and minimize impact.

In crop farming, Chaga has shown potential as a crop protection strategy and plant growth stimulant. It has been used to treat tree blight caused by *Cryphonectria parasitica* and protect cultivated plants from Phytophthora. The application of Chaga or its extracts has been associated with improved plant growth parameters, increased nutrient content, and enhanced resistance to plant diseases. Furthermore, Chaga mushrooms and their spent substrate have been explored as organic fertilizers, rich in organic matter and nutrients, to improve soil quality and fertility. The use of spent mushroom substrate not only enhances soil structure but also reduces waste and saves resources in agricultural practices.

The most recent pharmaceutical importance of Chaga mushrooms has been reviewed to cater to the huge increase in demand for mushroom derivatives like polysaccharides and antibacterial agents and for its industrial and medicinal uses. Scientists spent many years investigating this fungus before its possible pharmacological effects could be validated, despite extensive Indigenous knowledge indicating that the mushroom has long been utilized medicinally. In summary, this review serves as a benchmark for researchers to further investigate this fungus, to better understand its medicinal value and benefits for crop and animal production, particularly as a potential candidate for biofertilization, and to further exploit its remarkable significance in the cosmeceutical and food industries.

## References

[B1] Abu-Reidah I. M. Critch A. L. Manful C. F. Rajakaruna A. Vidal N. P. Pham T. H. (2021). Effects of pH and temperature on water under pressurized conditions in the extraction of nutraceuticals from chaga (Inonotus obliquus) mushroom. Antioxidants 10 (8), 1322. 10.3390/antiox10081322 34439572 PMC8389277

[B205] Akdeniz N. (2019). A systematic review of biochar use in animal waste composting. Waste Manag. 88, 291–300. 10.1016/j.wasman.2019.03.054 31079642

[B2] Alhallaf W. A. A. (2020). Investigation of anti-inflammatory and antioxidants properties of phenolic compounds from Inonotus obliquus using different extraction methods. The University of Maine.

[B3] Arata S. Watanabe J. Maeda M. Yamamoto M. Matsuhashi H. Mochizuki M. (2016). Continuous intake of the Chaga mushroom (Inonotus obliquus) aqueous extract suppresses cancer progression and maintains body temperature in mice. Heliyon 2 (5), e00111. 10.1016/j.heliyon.2016.e00111 27441282 PMC4946216

[B4] Asemoloye M. D. Marchisio M. A. Gupta V. K. Pecoraro L. (2021). Genome-based engineering of ligninolytic enzymes in fungi. Microb. cell factories 20, 20–18. 10.1186/s12934-021-01510-9 PMC7819241 33478513

[B5] Awad-Allah E. F. Shams A. H. Helaly A. A. Ragheb E. I. (2022). Effective applications of Trichoderma spp. as biofertilizers and biocontrol agents mitigate tomato Fusarium wilt disease. Agriculture 12 (11), 1950. 10.3390/agriculture12111950

[B219] Ayoub N. Lass D. Schultze W. (2009). Volatile constituents of the medicinal fungus chaga Inonotus obliquus (Pers: Fr.) Pilát (Aphyllophoromycetideae). Int. J. Med. Mushrooms. 11 (1).

[B6] Babitskaya V. Shcherba V. Lkonnikova N. (2000). Melanin complex of the fungus Inonotus obliquus. Appl. Biochem. Microbiol. 36 (4), 377–381. 10.1007/bf02738046 10994193

[B7] Baek G.-H. Jeong H.-S. Kim H. Yoon T.-J. Suh H.-J. Yu K.-W. (2012). Pharmacological activity of chaga mushroom on extraction conditions and immunostimulating polysaccharide. J. Korean Soc. Food Sci. Nutr. 41 (10), 1378–1387. 10.3746/jkfn.2012.41.10.1378

[B8] Baek J. Roh H.-S. Baek K.-H. Lee S. Lee S. Song S.-S. (2018). Bioactivity-based analysis and chemical characterization of cytotoxic constituents from Chaga mushroom (Inonotus obliquus) that induce apoptosis in human lung adenocarcinoma cells. J. Ethnopharmacol. 224, 63–75. 10.1016/j.jep.2018.05.025 29800742

[B9] Balandaykin M. E. Zmitrovich I. V. (2015). Review on Chaga medicinal mushroom, Inonotus obliquus (Higher Basidiomycetes): realm of medicinal applications and approaches on estimating its resource potential. Int. J. Med. Mushrooms 17 (2), 95–104. 10.1615/intjmedmushrooms.v17.i2.10 25746615

[B10] Beltrame G. Trygg J. Hemming J. Han Z. Yang B. (2021). Comparison of polysaccharides extracted from cultivated mycelium of Inonotus obliquus with polysaccharide fractions obtained from sterile conk (chaga) and birch heart rot. J. Fungi 7 (3), 189. 10.3390/jof7030189 PMC8000984 33800424

[B206] Barkwell L. J. (2018). La michinn: traditional metis medicines and healing. Winnepeg: Louis Riel Institute Press.

[B207] Basal W. T. Elfiky A. Eid J. (2021). Chaga medicinal mushroom inonotus obliquus (agaricomycetes) terpenoids may interfere with SARS-CoV-2 spike protein recognition of the host cell: a molecular docking study. Int. J. Med. mushrooms, 23(3), 1–14. 10.1615/IntJMedMushrooms.2021037942 33822495

[B208] Courtney R. Mullen G. (2008). Soil quality and barley growth as influenced by the land application of two compost types. Bioresour. Technol. 99(8), 2913–2918. 10.1016/j.biortech.2007.06.034 17702570

[B11] Bernicchia A. (2005). Polyporaceae sl Fungi europaei, 10.

[B12] Bogan B. W. Lamar R. Burgos W. Tien M. (1999). Extent of humification of anthracene, fluoranthene, and benzo[α]pyrene by*Pleurotus ostreatus*during growth in PAH‐contaminated soils. Lett. Appl. Microbiol. 28 (4), 250–254. 10.1046/j.1365-2672.1999.00537.x

[B13] Boulet B. Bussières G. (2018). Regard nouveau sur la biologie du polypore ponctué: fomitiporia punctata (P. Karsten) Murrill. Le. Nat. Can. 142 (3), 59–72. 10.7202/1050999ar

[B14] Brydon-Williams R. (2019). Distribution, presence, Ecology, and harvest Dynamics of the chaga fungus (Inonotus obliquus) in the white mountain. National Forest University of New Hampshire.

[B15] Bulatov P. Berezina M. M. Yakimov P. (1959). The'chaga'and its therapeutical use in stage IV cancer. The'chaga'and its therapeutical use in stage IV cancer.

[B16] Burczyk J. Gawron A. Slotwinska M. Smietana B. Terminska K. (1996). Antimitotic activity of aqueous extracts of Inonotus obliquus. Boll. Chim. Farm. 135 (5), 306–309. 8942059

[B17] Cajthaml T. Svobodová K. (2012). “Biodegradation of aromatic pollutants by ligninolytic fungal strains,” in Microbial degradation of Xenobiotics (Springer), I, 291–316.

[B18] Cha J.-Y. Jun B.-S. Yoo K.-S. Hahm J.-R. Cho Y.-S. (2006b). Fermented chaga mushroom (Inonotus obliquus) effects on hypolipidemia and hepatoprotection in Otsuka Long-Evans Tokushima fatty (OLETF) rats. Food Sci. Biotechnol. 15 (1), 122–127.

[B19] Cha J.-Y. Jun B.-S. Kim J.-W. Park S.-H. Lee C.-H. Cho Y.-S. (2006a). Hypoglycemic effects of fermented chaga mushroom (Inonotus obliquus) in the diabetic otsuka long-evans tokushima fatty (OLETF) rat. Food Sci. Biotechnol. 15 (5), 89–95.

[B20] Cha J.-Y. Jun B.-S. Lee C.-H. Yooi K.-S. Moon J.-C. Cho Y.-S. (2005). Hypoglycemic and antioxidative effects of fermented chaga mushroom (Inonotus obliquus) on streptozotocin-induced diabetic rats. J. Life Sci. 15 (5), 809–818.

[B21] Chae B. Lohakare J. Moon W. Lee S. Park Y. Hahn T.-W. (2006). Effects of supplementation of β-glucan on the growth performance and immunity in broilers. Res. veterinary Sci. 80 (3), 291–298. 10.1016/j.rvsc.2005.07.008 16165172

[B22] Chandrasekaran G. Oh D.-S. Shin H.-J. (2011). Properties and potential applications of the culinary-medicinal cauliflower mushroom, Sparassis crispa Wulf.: Fr. (Aphyllophoromycetideae): a review. Int. J. Med. Mushrooms 13 (2), 177–183. 10.1615/intjmedmushr.v13.i2.100 22135894

[B23] Chen H. Lu X. Qu Z. Wang Z. Zhang L. (2010). Glycosidase inhibitory activity and antioxidant properties of a polysaccharide from the mushroom Inonotus obliquus. J. Food Biochem. 34, 178–191. 10.1111/j.1745-4514.2009.00322.x

[B24] Chen H. Wang J. (2014). Phytochemistry, traditional uses and health benefits of the mushroom Inonotus obliquus (Chaga), I. Nova Science Publishers.

[B218] Chang y. Bai M. Xue X. B. Zou C. X. Huang X. X. Song S. J. (2022). Isolation of chemical compositions as dietary antioxidant supplements and neuroprotectants from Chaga mushroom (Inonotus obliquus). Food Biosci. 47, 101623.

[B25] Chung M. J. Chung C.-K. Jeong Y. Ham S.-S. (2010). Anticancer activity of subfractions containing pure compounds of Chaga mushroom (Inonotus obliquus) extract in human cancer cells and in Balbc/c mice bearing Sarcoma-180 cells. Nutr. Res. Pract. 4 (3), 177–182. 10.4162/nrp.2010.4.3.177 20607061 PMC2895696

[B26] Cueno R. Morillo T. Carter S. Lachmann M. Park J. Schneider J. (2004). Evaluation of Beta-glucan and antibiotics on growth performance and Carcass Traits of Weanling and finishing pigs. Copyright.

[B27] Cui Y. Kim D.-S. Park K.-C. (2005). Antioxidant effect of Inonotus obliquus. J. Ethnopharmacol. 96 (1-2), 79–85. 10.1016/j.jep.2004.08.037 15588653

[B28] Cunha Zied D. Sánchez J. E. Noble R. Pardo-Giménez A. (2020). Use of spent mushroom substrate in new mushroom crops to promote the transition towards a circular economy. Agronomy 10 (9), 1239. 10.3390/agronomy10091239

[B29] Dashtban M. Schraft H. Syed T. A. Qin W. (2010). Fungal biodegradation and enzymatic modification of lignin. Int. J. Biochem. Mol. Biol. 1 (1), 36–50. 21968746 PMC3180040

[B30] Debnath T. Park S. R. Kim D. H. Jo J. E. Lim B. O. (2013). Anti-oxidant and anti-inflammatory activities of Inonotus obliquus and germinated brown rice extracts. Molecules 18 (8), 9293–9304. 10.3390/molecules18089293 23917116 PMC6270324

[B31] Deshpande A. G. Arya A. (2022). “Mushroom biotechnology: developing cultivation protocol for four different mushrooms and accessing their potential in pollution management,” in Biology, cultivation and applications of mushrooms (Springer), 457–485.

[B32] Dritz S. Shi J. Kielian T. Goodband R. Nelssen J. Tokach M. (1995). Influence of dietary β-glucan on growth performance, nonspecific immunity, and resistance to Streptococcus suis infection in weanling pigs. J. Animal Sci. 73 (11), 3341–3350. 10.2527/1995.73113341x 8586593

[B33] Eid J. I. Das B. Al‐Tuwaijri M. M. Basal W. T. (2021). Targeting SARS‐CoV‐2 with Chaga mushroom: an *in silico* study toward developing a natural antiviral compound. Food Sci. Nutr. 9 (12), 6513–6523. 10.1002/fsn3.2576 34900242 PMC8645752

[B34] Esmaielpour B. Rahmanian M. Heidarpour O. Shahriari M. H. (2017). Effect of vermicompost and spent mushroom compost on the nutrient and essential oil composition of basil (Ocimum basilicum L.). J. Essent. Oil Bear. Plants 20 (5), 1283–1292. 10.1080/0972060x.2017.1396931

[B35] Falck R. (1902). Die Cultur der Oidien und ihre Rückführung in die höhere Fruchtform bei den Basidiomyceten. Druck Nischkowsky.

[B36] Federhen S. (2012). The NCBI taxonomy database. Nucleic acids Res. 40 (D1), D136–D143. 10.1093/nar/gkr1178 22139910 PMC3245000

[B37] Geng Y. Lu Z.-M. Huang W. Xu H.-Y. Shi J.-S. Xu Z.-H. (2013). Bioassay-guided isolation of DPP-4 inhibitory fractions from extracts of submerged cultured of Inonotus obliquus. Molecules 18 (1), 1150–1161. 10.3390/molecules18011150 23325103 PMC6270506

[B38] Géry A. Dubreule C. André V. Rioult J.-P. Bouchart V. Heutte N. (2018). Chaga (Inonotus obliquus), a future potential medicinal fungus in oncology? A chemical study and a comparison of the cytotoxicity against human lung adenocarcinoma cells (A549) and human bronchial epithelial cells (BEAS-2B). Integr. cancer Ther. 17 (3), 832–843. 10.1177/1534735418757912 29484963 PMC6142110

[B210] Grimm D. Wösten H. A. (2018). Mushroom cultivation in the circular economy. Appl. Microbiol. Biotechnol. 102, 7795–7803. 10.1007/s00253-018-9226-8 30027491 PMC6132538

[B39] Giridharan V. V. Thandavarayan R. A. Konishi T. (2011). Amelioration of scopolamine induced cognitive dysfunction and oxidative stress by Inonotus obliquus–a medicinal mushroom. Food & Funct. 2 (6), 320–327. 10.1039/c1fo10037h 21779570

[B40] Glamočlija J. Ćirić A. Nikolić M. Fernandes Â. Barros L. Calhelha R. C. (2015). Chemical characterization and biological activity of Chaga (Inonotus obliquus), a medicinal “mushroom”. J. Ethnopharmacol. 162, 323–332. 10.1016/j.jep.2014.12.069 25576897

[B41] Gogineni V. Schinazi R. F. Hamann M. T. (2015). Role of marine natural products in the genesis of antiviral agents. Chem. Rev. 115 (18), 9655–9706. 10.1021/cr4006318 26317854 PMC4883660

[B42] Gorbunova I. A. Perova N. Teplyakova T. V. (2005). Medicinal mushrooms of southwest Siberia. Int. J. Med. Mushrooms 7 (3), 403–404. 10.1615/intjmedmushr.v7.i3.480

[B43] Gründemann C. Reinhardt J. K. Lindequist U. (2020). European medicinal mushrooms: do they have potential for modern medicine? An update. Phytomedicine 66, 153131. 10.1016/j.phymed.2019.153131 31790898

[B44] Hadar Y. Cohen-Arazi E. (1986). Chemical composition of the edible mushroom Pleurotus ostreatus produced by fermentation. Appl. Environ. Microbiol. 51 (6), 1352–1354. 10.1128/AEM.51.6.1352-1354.1986 16347090 PMC239070

[B45] Hadar Y. Kerem Z. Gorodecki B. Ardon O. (1992). “Utilization of lignocellulosic waste by the edible mushroom, Pleurotus,” in Microorganisms to combat pollution (Springer), 65–81.

[B46] Haines A. (2013). Review of medical uses of chaga {Inonotus obliquus}. Available at: http://www.wildfeder.com/ .

[B47] Ham S.-S. Kim S.-H. Moon S.-Y. Chung M. J. Cui C.-B. Han E.-K. (2009). Antimutagenic effects of subfractions of Chaga mushroom (Inonotus obliquus) extract. Mutat. Research/Genetic Toxicol. Environ. Mutagen. 672 (1), 55–59. 10.1016/j.mrgentox.2008.10.002 18992843

[B48] Handa N. Yamada T. Tanaka R. (2010). An unusual lanostane-type triterpenoid, spiroinonotsuoxodiol, and other triterpenoids from Inonotus obliquus. Phytochemistry 71 (14-15), 1774–1779. 10.1016/j.phytochem.2010.07.005 20691456

[B49] He J. Feng X.-Z. Lu Y. Zhao B. (2001). Three new triterpenoids from Fuscoporia obliqua. J. Asian Nat. Prod. Res. 3 (1), 55–61. 10.1080/10286020108042839 11355771

[B50] Hearst R. Nelson D. McCollum G. Millar B. C. Maeda Y. Goldsmith C. E. (2009). An examination of antibacterial and antifungal properties of constituents of Shiitake (Lentinula edodes) and Oyster (Pleurotus ostreatus) mushrooms. Complementary Ther. Clin. Pract. 15 (1), 5–7. 10.1016/j.ctcp.2008.10.002 19161947

[B51] Heo J.-W. No M.-H. Park D.-H. Kang J.-H. Seo D. Y. Han J. (2017). Effects of exercise on obesity-induced mitochondrial dysfunction in skeletal muscle. Korean J. Physiology Pharmacol. 21 (6), 567–577. 10.4196/kjpp.2017.21.6.567 PMC5709473 29200899

[B52] Hobbs C. (2003). Medicinal mushrooms: an exploration of tradition, health and culture. Botanica press.

[B53] Honda Y. Matsuyama T. Irie T. Watanabe T. Kuwahara M. (2000). Carboxin resistance transformation of the homobasidiomycete fungus Pleurotus ostreatus. Curr. Genet. 37 (3), 209–212. 10.1007/s002940050521 10794179

[B54] Hong K. B. Noh D. O. Park Y. Suh H. J. (2015). Hepatoprotective activity of water extracts from chaga medicinal mushroom, Inonotus obliquus (higher basidiomycetes) against tert-butyl Hydroperoxide− induced oxidative liver injury in primary cultured rat hepatocytes. Int. J. Med. Mushrooms 17 (11), 1069–1076. 10.1615/intjmedmushrooms.v17.i11.70 26853962

[B55] Hordyjewska A. Ostapiuk A. Horecka A. Kurzepa J. (2019). Betulin and betulinic acid: triterpenoids derivatives with a powerful biological potential. Phytochem. Rev. 18, 929–951. 10.1007/s11101-019-09623-1

[B212] Mohd Hanafi F. H. Rezania S. Mat Taib S. Md Din M. F. Yamauchi M. Sakamoto M. (2018). Environmentally sustainable applications of agro-based spent mushroom substrate (SMS): an overview. J. Material Cycles Waste Manag. 20, 1383–1396. 10.1007/s10163-018-0739-0

[B56] Hu H. Zhang Z. Lei Z. Yang Y. Sugiura N. (2009). Comparative study of antioxidant activity and antiproliferative effect of hot water and ethanol extracts from the mushroom Inonotus obliquus. J. Biosci. Bioeng. 107 (1), 42–48. 10.1016/j.jbiosc.2008.09.004 19147108

[B57] Hu T. Liu P. Ni Y. Lu C. (2012). Isolation, purification and effects of hypoglycemic functional polysaccharides from Inonotus obliquus. Afr. J. Biotechnol. 11 (30), 7738–7743. 10.5897/AJB11.3536

[B58] Huang S.-q. Ding S. Fan L. (2012). Antioxidant activities of five polysaccharides from Inonotus obliquus. Int. J. Biol. Macromol. 50 (5), 1183–1187. 10.1016/j.ijbiomac.2012.03.019 22484729

[B59] Hüttermann A. Hamza A. Chet I. Majcherczyk A. Fouad T. Badr A. (2000). Recycling of agricultural wastes by white-rot fungi for the production of fodder for ruminants. Agro Food Ind. Hi-Tech 11 (6), 29–32.

[B60] Huynh N. (2019). Supercritical CO2 extraction of triterpenoids from Inonotus obliquus University of Turku]. Finland. 10.3390/molecules27061880 PMC8955864 35335249

[B61] Hwang A. Y. Yang S. C. Kim J. Lim T. Cho H. Hwang K. T. (2019). Effects of non-traditional extraction methods on extracting bioactive compounds from chaga mushroom (Inonotus obliquus) compared with hot water extraction. LWT 110, 80–84. 10.1016/j.lwt.2019.04.073

[B62] Hyun K. W. Jeong S. C. Lee D. H. Park J. S. Lee J. S. (2006). Isolation and characterization of a novel platelet aggregation inhibitory peptide from the medicinal mushroom, Inonotus obliquus. Peptides 27 (6), 1173–1178. 10.1016/j.peptides.2005.10.005 16289471

[B63] Ilić N. Milić M. Beluhan S. Dimitrijević-Branković S. (2023). Cellulases: from lignocellulosic biomass to improved production. Energies, 16(8), 3598. 10.3390/en16083598

[B64] Il’ina I. Uljašev O. (2012). Gender and myth in traditional Komi-Zyrjan culture, 308. Mythic Discourses.

[B65] Im K. H. Nguyen T. K. Choi J. Lee T. S. (2016). *In vitro* antioxidant, anti-diabetes, anti-dementia, and inflammation inhibitory effect of Trametes pubescens fruiting body extracts. Molecules 21 (5), 639. 10.3390/molecules21050639 27196881 PMC6273937

[B66] Irie T. Honda Y. Watanabe T. Kuwahara M. (2001). Efficient transformation of filamentous fungus Pleurotus ostreatus using single-strand carrier DNA. Appl. Microbiol. Biotechnol. 55 (5), 563–565. 10.1007/s002530000535 11414321

[B67] Ishfaq P. M. Mishra A. Mishra S. Ahmad Z. Gayen S. Jain S. K. (2021). Inonotus obliquus aqueous extract suppresses carbon tetrachloride-induced hepatic injury through modulation of antioxidant enzyme system and anti-inflammatory mechanism. Clin. Cancer Drugs 8 (2), 122–136. 10.2174/2212697x08666211130130119

[B68] Ishfaq P. M. Mishra S. Mishra A. Ahmad Z. Gayen S. Jain S. K. (2022). Inonotus obliquus aqueous extract prevents histopathological alterations in liver induced by environmental toxicant Microcystin. Curr. Res. Pharmacol. Drug Discov. 3, 100118. 10.1016/j.crphar.2022.100118 35992377 PMC9389225

[B69] Jayachandran M. Xiao J. Xu B. (2017). A critical review on health promoting benefits of edible mushrooms through gut microbiota. Int. J. Mol. Sci. 18 (9), 1934. 10.3390/ijms18091934 28885559 PMC5618583

[B70] Jiang S. Shi F. Lin H. Ying Y. Luo L. Huang D. (2020). Inonotus obliquus polysaccharides induces apoptosis of lung cancer cells and alters energy metabolism via the LKB1/AMPK axis. Int. J. Biol. Macromol. 151, 1277–1286. 10.1016/j.ijbiomac.2019.10.174 31751687

[B71] Jin T. Xiaoliang H. Dafei L. Hongxia W. Liandong Q. (2017). Identification of Inonotus obliquus polysaccharide with broad-spectrum antiviral activity against multi-feline viruses. Int. J. Biol. Macromol. 95, 160–167. 10.1016/j.ijbiomac.2016.11.054 27865960 PMC7185483

[B72] Joo J. I. Kim D. H. Yun J. W. (2010). Extract of Chaga mushroom (Inonotus obliquus) stimulates 3t3‐l1 adipocyte differentiation. Phytotherapy Res. 24 (11), 1592–1599. 10.1002/ptr.3180 21031614

[B73] Jung H.-K. Hong J.-H. Park S.-C. Park B.-K. Nam D.-H. Kim S.-D. (2007). Production and physicochemical characterization of β-glucan produced byPaenibacillus polymyxa JB115. Biotechnol. Bioprocess Eng. 12 (6), 713–719. 10.1007/bf02931090

[B74] Kahlos K. Hiltunen R. v Schantz M. (1984). 3beta-Hydroxy-lanosta-8,24-dien-21-al, a new triterpene from inontus obliquus. Planta medica. 50 (02), 197–198. 10.1055/s-2007-969674 17340294

[B75] Kamra D. Zadražil F. (1986). Influence of gaseous phase, light and substrate pretreatment on fruit-body formation, lignin degradation and *in vitro* digestibility of wheat straw fermented with Pleurotus spp. Agric. wastes 18 (1), 1–17. 10.1016/0141-4607(86)90103-4

[B76] Kang J.-H. Jang J.-E. Mishra S. K. Lee H.-J. Nho C. W. Shin D. (2015). Ergosterol peroxide from Chaga mushroom (Inonotus obliquus) exhibits anti-cancer activity by down-regulation of the β-catenin pathway in colorectal cancer. J. Ethnopharmacol. 173, 303–312. 10.1016/j.jep.2015.07.030 26210065

[B77] Kaur R. Tyagi R. D. Zhang X. (2020). Review on pulp and paper activated sludge pretreatment, inhibitory effects and detoxification strategies for biovalorization. Environ. Res. 182, 109094. 10.1016/j.envres.2019.109094 31927243

[B78] Khan M. W. Ali M. A. Khan N. A. Khan M. A. Rehman A. Javed N. (2013). Effect of different levels of lime and pH on mycelial growth and production efficiency of oyster mushroom (Pleurotus spp.). Pak. J. Bot. 45 (1), 297–302.

[B79] Kikuchi Y. Seta K. Ogawa Y. Takayama T. Nagata M. Taguchi T. (2014). Chaga mushroom-induced oxalate nephropathy. Clin. Nephrol. 81 (6), 440–444. 10.5414/CN107655 23149251

[B80] Kim T. I. Choi J.-G. Kim J. H. Li W. Chung H.-S. (2020). Blocking effect of chaga mushroom (Inonotus oliquus) extract for immune checkpoint CTLA-4/CD80 interaction. Appl. Sci. 10 (17), 5774. 10.3390/app10175774

[B81] Kim Y. J. Park J. Min B. S. Shim S. H. (2011). Chemical constituents from the sclerotia of Inonotus obliquus. J. Korean Soc. Appl. Biol. Chem. 54 (2), 287–294. 10.3839/jksabc.2011.045

[B82] Kim Y. O. Park H. W. Kim J. H. Lee J. Y. Moon S. H. Shin C. S. (2006). Anti-cancer effect and structural characterization of endo-polysaccharide from cultivated mycelia of Inonotus obliquus. Life Sci. 79 (1), 72–80. 10.1016/j.lfs.2005.12.047 16458328

[B83] Kim Y.-R. (2005). Immunomodulatory activity of the water extract from medicinal mushroom Inonotus obliquus. Mycobiology 33 (3), 158–162. 10.4489/MYCO.2005.33.3.158 24049493 PMC3774877

[B84] Kirk P. M. Cannon P. F. Minter D. Stalpers J. (2008). Dictionary of the fungi. 10th edition. Wallingford, UK: CAB International.

[B85] Ko S.-k. Jin M. Pyo M.-y. (2011). Inonotus obliquus extracts suppress antigen-specific IgE production through the modulation of Th1/Th2 cytokines in ovalbumin-sensitized mice. J. Ethnopharmacol. 137 (3), 1077–1082. 10.1016/j.jep.2011.07.024 21820502

[B86] Kou R.-W. Han R. Gao Y.-Q. Li D. Yin X. Gao J.-M. (2021). Anti-neuroinflammatory polyoxygenated lanostanoids from Chaga mushroom Inonotus obliquus. Phytochemistry 184, 112647. 10.1016/j.phytochem.2020.112647 33434790

[B87] Kukulyanskaya T. Kurchenko N. Kurchenko V. Babitskaya V. (2002). Physicochemical properties of melanins produced by the sterile form of Inonotus obliquus (“Chagi”) in natural and cultivated fungus. Appl. Biochem. Microbiol. 38 (1), 58–61. 10.1023/a:1013204706055 11852571

[B88] Kumar P. Bhadauria A. S. Singh A. K. Saha S. (2018). Betulinic acid as apoptosis activator: molecular mechanisms, mathematical modeling and chemical modifications. Life Sci. 209, 24–33. 10.1016/j.lfs.2018.07.056 30076920

[B89] Kyoung P. Y. Burm L. H. Eun-Jae J. Sung J. H. Myung-Hee K. (2004). Chaga mushroom extract inhibits oxidative DNA damage in human lymphocytes as assessed by comet assay. Biofactors 21 (1-4), 109–112. 10.1002/biof.552210120 15630179

[B90] Lee C. K. Ibrahim D. Omar I. C. (2013). Enzymatic deinking of various types of waste paper: efficiency and characteristics. Process Biochem. 48 (2), 299–305. 10.1016/j.procbio.2012.12.015

[B91] Lee I.-K. Kim Y.-S. Jang Y.-W. Jung J.-Y. Yun B.-S. (2007). New antioxidant polyphenols from the medicinal mushroom Inonotus obliquus. Bioorg. Med. Chem. Lett. 17 (24), 6678–6681. 10.1016/j.bmcl.2007.10.072 17980585

[B92] Lee I.-K. Yun B.-S. (2006). Hispidin analogs from the mushroom Inonotus xeranticus and their free radical scavenging activity. Bioorg. Med. Chem. Lett. 16 (9), 2376–2379. 10.1016/j.bmcl.2006.01.121 16488146

[B93] Lee K. Nagajyothi P. Sreekanth T. Park S. (2015). Eco-friendly synthesis of gold nanoparticles (AuNPs) using Inonotus obliquus and their antibacterial, antioxidant and cytotoxic activities. J. Industrial Eng. Chem. 26, 67–72. 10.1016/j.jiec.2014.11.016

[B94] Lee M.-W. Hur H. Chang K.-C. Lee T.-S. Ka K.-H. Jankovsky L. (2008). Introduction to distribution and ecology of sterile conks of Inonotus obliquus. Mycobiology 36 (4), 199–202. 10.4489/MYCO.2008.36.4.199 23997626 PMC3755195

[B95] Lee S. Lee H. Y. Park Y. Ko E. J. Ban T. H. Chung B. H. (2020). Development of end stage renal disease after long-term ingestion of chaga mushroom: case report and review of literature. J. Korean Med. Sci. 35 (19), e122. 10.3346/jkms.2020.35.e122 32419395 PMC7234858

[B96] Lee S. H. Hwang H. S. Yun J. W. (2009). Antitumor activity of water extract of a mushroom, Inonotus obliquus, against HT‐29 human colon cancer cells. Phytotherapy Res. 23 (12), 1784–1789. 10.1002/ptr.2836 19367670

[B97] Lemieszek M. K. Langner E. Kaczor J. Kandefer-Szerszen M. Sanecka B. Mazurkiewicz W. (2011). Anticancer effects of fraction isolated from fruiting bodies of Chaga medicinal mushroom, Inonotus obliquus (Pers.: Fr.) Pilát (Aphyllophoromycetideae): *in vitro* studies. Int. J. Med. Mushrooms 13 (2), 131–143. 10.1615/intjmedmushr.v13.i2.50 22135889

[B98] Li J. Li D. Xing J. Cheng Z. Lai C. (2006). Effects of β-glucan extracted from Saccharomyces cerevisiae on growth performance, and immunological and somatotropic responses of pigs challenged with Escherichia coli lipopolysaccharide. J. Animal Sci. 84 (9), 2374–2381. 10.2527/jas.2004-541 16908640

[B99] Li N. Senotrusova T. Son O. Podvolotskaya A. Ershova T. Levchuk T. (2022). APPLICATION OF A NATURAL ANTIOXIDANT FOR PROTECT FEED VITAMIN A. J. Agric. Environ. 8 (28). 10.23649/jae.2022.28.8.008

[B100] Liang L. Zhang Z. Wang H. (2009). Antioxidant activities of extracts and subfractions from Inonotus Obliquus. Int. J. food Sci. Nutr. 60 (2), 175–184. 10.1080/09637480903042279 19585318

[B101] Lindenfelser L. Detroy R. Ramstack J. Worden K. (1979). Biological modification of the lignin and cellulose components of wheat straw by Pleurotus ostreatus. Dev. Ind. Microbiol. 20, 541–551.

[B102] Lindequist U. Niedermeyer T. H. Jülich W.-D. (2005). The pharmacological potential of mushrooms. Evidence-based complementary Altern. Med. 2, 285–299. 10.1093/ecam/neh107 PMC1193547 16136207

[B103] Liu C. Zhao C. Pan H.-H. Kang J. Yu X.-T. Wang H.-Q. (2014). Chemical constituents from Inonotus obliquus and their biological activities. J. Nat. Prod. 77 (1), 35–41. 10.1021/np400552w 24359303

[B104] Liuping F. Shaodong D. Lianzhong A. Kequan D. (2012). Antitumor and immunomodulatory activity of water-soluble polysaccharide from Inonotus obliquus. Carbohydr. Polym. 90 (2), 870–874. 10.1016/j.carbpol.2012.06.013 22840014

[B105] Lou Z. Sun Y. Bian S. Baig S. A. Hu B. Xu X. (2017). Nutrient conservation during spent mushroom compost application using spent mushroom substrate derived biochar. Chemosphere 169, 23–31. 10.1016/j.chemosphere.2016.11.044 27855328

[B106] Lu X. Chen H. Dong P. Fu L. Zhang X. (2010). Phytochemical characteristics and hypoglycaemic activity of fraction from mushroom Inonotus obliquus. J. Sci. Food Agric. 90 (2), 276–280. 10.1002/jsfa.3809 20355042

[B107] Lukina N. (1975). Narodnye sredstva po sohraneniyu zdorovya i zhizni u vostochnyh khantov, Etnograficheskiye aspekty izucheniya narodnoi meditsiny, Tezisy vsesoyuznoi nauchnoi konferentsii 1–12 marta 1975 g, 26–27. Avaiable at: https://www.scopus.com/inward/record.uri?eid=2-s2.0-84915375596&partnerID=40&md5=6b2ad12f9337c69396f449e54962ea96 .

[B108] Lumlertgul N. Siribamrungwong M. Jaber B. L. Susantitaphong P. (2018). Secondary oxalate nephropathy: a systematic review. Kidney Int. Rep. 3 (6), 1363–1372. 10.1016/j.ekir.2018.07.020 30450463 PMC6224620

[B109] Ma L. Chen H. Dong P. Lu X. (2013). Anti-inflammatory and anticancer activities of extracts and compounds from the mushroom Inonotus obliquus. Food Chem. 139 (1-4), 503–508. 10.1016/j.foodchem.2013.01.030 23561137

[B110] May T. W. Redhead S. A. Bensch K. Hawksworth D. L. Lendemer J. Lombard L. (2019). Chapter F of the international code of nomenclature for algae, fungi, and plants as approved by the 11th international mycological congress, san juan, Puerto Rico. IMA fungus 10 (1), 1–14. 10.1186/s43008-019-0019-1 32647625 PMC7325661

[B111] Meng X. Dai J. Zhang Y. Wang X. Zhu W. Yuan X. (2018). Composted biogas residue and spent mushroom substrate as a growth medium for tomato and pepper seedlings. J. Environ. Manag. 216, 62–69. 10.1016/j.jenvman.2017.09.056 28958462

[B211] Meng X. Liu B. Zhang H. Wu J. Yuan X. Cui Z. (2019). Co-composting of the biogas residues and spent mushroom substrate: physicochemical properties and maturity assessment. Bioresour. Technol. 276, 281 –287. 10.1016/j.biortech.2018.12.097 30640023

[B112] Milyuhina A. Kyzdarbek U. Romazyaeva I. (2022). Assessment of antimicrobial activity of medicinal plants’ extracts. AIP Conf. Proc. 10.1063/5.0071327

[B113] Mishra S. Kang J. Song K. Park M. Kim D. Park Y. (2013). Inonotus obliquus suppresses proliferation of colorectal cancer cells and tumor growth in mice models by downregulation of β-catenin/NF-κB-signaling pathways. Eur. J. Inflamm. 11 (3), 615–629. 10.1177/1721727x1301100306

[B114] Mishra S. K. Kang J.-H. Kim D.-K. Oh S. H. Kim M. K. (2012). Orally administered aqueous extract of Inonotus obliquus ameliorates acute inflammation in dextran sulfate sodium (DSS)-induced colitis in mice. J. Ethnopharmacol. 143 (2), 524–532. 10.1016/j.jep.2012.07.008 22819687

[B115] Mizuno T. Zhuang C. Abe K. Okamoto H. Kiho T. Ukai S. (1999). Antitumor and hypoglycemic activities of polysaccharides from the sclerotia and mycelia of Inonotus obliquus (Pers.: Fr.) Pil. (Aphyllophoromycetideae). Int. J. Med. Mushrooms 1 (4), 301–316. 10.1615/intjmedmushr.v1.i4.20

[B116] Moon B.-H. Lee W.-C. (2009). Studies on the anti-cancer activity of chaga mushroom extract. J. Korean Med. 30 (4), 1–12.

[B117] Moore P. Evenson A. Luckey T. McCoy E. Elvehjem C. Hart E. (1946). Use of sulfasuxidine, streptothricin, and streptomycin in nutritional studies with the chick. J. Biol. Chem. 165 (2), 437–441. 10.1016/s0021-9258(17)41154-9 20276107

[B118] Moss R. W. (2016). Chaga mushroom and cancer. Retrieved January, 16, 2023 from http://www.nrocrc.com/news/headline_news/2016/07/16/160.html.

[B119] Mu H. Zhang A. Zhang W. Cui G. Wang S. Duan J. (2012). Antioxidative properties of crude polysaccharides from Inonotus obliquus. Int. J. Mol. Sci. 13 (7), 9194–9206. 10.3390/ijms13079194 22942760 PMC3430291

[B120] Muswati C. Simango K. Tapfumaneyi L. Mutetwa M. Ngezimana W. (2021). The effects of different substrate combinations on growth and yield of oyster mushroom (Pleurotus ostreatus). Int. J. Agron. 2021, 1–10. 10.1155/2021/9962285

[B121] Najafzadeh M. Reynolds P. D. Baumgartner A. Jerwood D. Anderson D. (2007). Chaga mushroom extract inhibits oxidative DNA damage in lymphocytes of patients with inflammatory bowel disease. Biofactors 31 (3), 191–200. 10.1002/biof.5520310306 18997282

[B122] Nakajima Y. Sato Y. Konishi T. (2007). Antioxidant small phenolic ingredients in Inonotus obliquus (persoon) Pilat (Chaga). Chem. Pharm. Bull. 55 (8), 1222–1226. 10.1248/cpb.55.1222 17666849

[B123] Nakata T. Yamada T. Taji S. Ohishi H. Wada S.-i. Tokuda H. (2007). Structure determination of inonotsuoxides A and B and *in vivo* anti-tumor promoting activity of inotodiol from the sclerotia of Inonotus obliquus. Bioorg. Med. Chem. 15 (1), 257–264. 10.1016/j.bmc.2006.09.064 17049251

[B124] Navid M. H. Laszczyk-Lauer M. Reichling J. Schnitzler P. (2014). Pentacyclic triterpenes in birch bark extract inhibit early step of herpes simplex virus type 1 replication. Phytomedicine, 21(11), 1273–1280. 10.1016/j.phymed.2014.06.007 25172789

[B125] Ning X. Luo Q. Li C. Ding Z. Pang J. Zhao C. (2014). Inhibitory effects of a polysaccharide extract from the Chaga medicinal mushroom, Inonotus obliquus (higher Basidiomycetes), on the proliferation of human neurogliocytoma cells. Int. J. Med. Mushrooms 16 (1), 29–36. 10.1615/intjmedmushr.v16.i1.30 24940902

[B126] Nwafor S. O. Adenipekun C. O. Aruwa G. Asemoloye M. D. (2022). Biotreatment of selected agrowaste products with some culinary-medicinal mushrooms enhances their nutrient composition for use as animal feed in Nigeria. Int. J. Med. Mushrooms 24 (6), 57–68. 10.1615/IntJMedMushrooms.2022043952 35695638

[B127] Oliveira-Costa J. F. Meira C. S. Neves M. V. G. d. Dos Reis B. P. Z. C. Soares M. B. P. (2022). Anti-inflammatory activities of betulinic acid: a review. Front. Pharmacol., 13, 883857. 10.3389/fphar.2022.883857 35677426 PMC9168372

[B128] Othman N. Z. Sarjuni M. N. H. Rosli M. A. Nadri M. H. Yeng L. H. Ying O. P. (2020). Spent mushroom substrate as biofertilizer for agriculture application. Valoris. Agro-industrial Residues, 37–57. Volume I: Biological Approaches. 10.1007/978-3-030-39137-9_2

[B129] Paduch R. Kandefer-Szerszen M. (2014). Antitumor and antiviral activity of pentacyclic triterpenes. Mini. Rev. Org. Chem. 11, 262–268. 10.2174/1570193x1103140915105240

[B130] Pan H.-h. Yu X.-t. Li T. Wu H.-l. Jiao C.-w. Cai M.-h. (2013). Aqueous extract from a Chaga medicinal mushroom, Inonotus obliquus (higher Basidiomycetes), prevents herpes simplex virus entry through inhibition of viral-induced membrane fusion. Int. J. Med. Mushrooms 15 (1), 29–38. 10.1615/intjmedmushr.v15.i1.40 23510282

[B131] Park S. Shin H. Park D. Kim H. Byun Y. Lee K. Y. (2021). Structure elucidation of a new triterpene from Inonotus obliquus. Magnetic Reson. Chem. 59 (4), 489–494. 10.1002/mrc.5102 32959923

[B132] Park Y. K. Lee H. B. Jeon E.-J. Jung H. S. Kang M.-H. (2004). Chaga mushroom extract inhibits oxidative DNA damage in human lymphocytes as assessed by comet assay. Biofactors 21 (1-4), 109–112. 10.1002/biof.552210120 15630179

[B133] Park Y.-M. Won J.-H. Kim Y.-H. Choi J.-W. Park H.-J. Lee K.-T. (2005). *In vivo* and *in vitro* anti-inflammatory and anti-nociceptive effects of the methanol extract of Inonotus obliquus. J. Ethnopharmacol. 101 (1-3), 120–128. 10.1016/j.jep.2005.04.003 15905055

[B134] Pavithra S. Jayaprakash J. Gummadi S. N. Giri Dev V. R. (2023). Assessment of process integration approach for coir biosoftening and lignin‐modifying enzyme production from agro residues. Biofuels, Bioprod. Biorefining, 17, 921–932. 10.1002/bbb.2484

[B135] Peng H. Shahidi F. (2022). Qualitative analysis of secondary metabolites of chaga mushroom (Inonotus Obliquus): phenolics, fatty acids, and terpenoids. J. Food Bioact. 17. 10.31665/jfb.2022.17304

[B136] Pilz D. (2004). Chaga and other fungal resources: assessment of sustainable commercial harvesting in Khabarovsk and Primorsky Krais, Russia. Khabarovsk, Russia: Report prepared for Winrock International, Morrilton, Arkansas and the FOREST Project.

[B137] Polkovnikova M. Nosik N. Garaev T. Kondrashina N. Finogenova M. Shibnev V. (2014). A study of the antiherpetic activity of the chaga mushroom (Inonotus obliquus) extracts in the Vero cells infected with the herpes simplex virus. Vopr. Virusol. 59 (2), 45–48. 25069286

[B138] Rathgeber B. Budgell K. MacIsaac J. Mirza M. Doncaster K. (2008). Growth performance and spleen and bursa weight of broilers fed yeast beta-glucan. Can. J. animal Sci. 88 (3), 469–473. 10.4141/cjas07101

[B139] Razumov E. Y. Safin R. Mukhametzyanov S. R. Baigildeeva E. Safina A. Lebedev D. (2020). Studies of the composition of the cryogenic ground chaga. IOP Conf. Ser. Mater. Sci. Eng. 986, 012029. 10.1088/1757-899x/986/1/012029

[B140] Rhee S. J. Cho S. Y. Kim K. M. Cha D.-S. Park H.-J. (2008). A comparative study of analytical methods for alkali-soluble β-glucan in medicinal mushroom, Chaga (Inonotus obliquus). LWT-Food Sci. Technol. 41 (3), 545–549. 10.1016/j.lwt.2007.03.028

[B141] Rogers R. (2012). The true tinder conk: first Nation’s use. Fungi 5 (3), 56–57.

[B142] Rogers R. (2016). Mushroom essences: vibrational healing from the kingdom fungi. North Atlantic Books.

[B143] Rogers R. D. (2006). The fungal pharmacy: medicinal mushrooms of Western Canada. Prairie Deva Press. Retrieved January, 16, 2023 from Avaiable at: https://www.herbalgram.org/resources/herbalgram/issues/79/table-of-contents/article3297/ .

[B144] Roy S. Barman S. Chakraborty U. Chakraborty B. (2015). Evaluation of spent mushroom substrate as biofertilizer for growth improvement of Capsicum annuum L. J. Appl. Biol. Biotechnol. 3 (3), 0–2. 10.7324/JABB.2015.3305

[B145] Ryzhova G. Kravtsova S. Matasova S. Gribel H. (1997). Chemical and Parmacuetical characteristics of dried Chaga extract. Chemistry-pharmaceutical J. 10, 44–47.

[B146] Saar M. (1991). Fungi in Khanty folk medicine. J. Ethnopharmacol. 31 (2), 175–179. 10.1016/0378-8741(91)90003-v 2023426

[B147] Safin R. R. Razumov E. Y. Khasanshin R. R. Gubernatorov V. V. Saerova K. V. (2018). Effect of growth conditions on qualitative characteristics of chaga mushroom. Int. Multidiscip. Sci. GeoConference SGEM 18 (3.2), 781–788. 10.5593/sgem2018/3.2/S14.100

[B148] Sagayama K. Tanaka N. Fukumoto T. Kashiwada Y. (2019). Lanostane-type triterpenes from the sclerotium of Inonotus obliquus (Chaga mushrooms) as proproliferative agents on human follicle dermal papilla cells. J. Nat. Med. 73 (3), 597–601. 10.1007/s11418-019-01280-0 30706371

[B149] Saha S. Ghosh M. Dutta S. K. (2015). A potent tumoricidal co-drug ‘Bet-CA’-an ester derivative of betulinic acid and dichloroacetate selectively and synergistically kills cancer cells. Sci. Rep. 5 (1), 7762. 10.1038/srep07762 25585916 PMC4293591

[B150] Salmones D. Mata G. Waliszewski K. N. (2005). Comparative culturing of Pleurotus spp. on coffee pulp and wheat straw: biomass production and substrate biodegradation. Bioresour. Technol. 96 (5), 537–544. 10.1016/j.biortech.2004.06.019 15501659

[B213] Sánchez C. (2010). Cultivation of Pleurotus ostreatus and other edible mushrooms. Appl. Microbiol. Biotechnol. 85, 1321–1337. 10.1007/s00253-009-2343-7 19956947

[B151] Satoru A. Jun W. Masako M. Masato Y. Hideto M. Mamiko M. (2016). Continuous intake of the Chaga mushroom (Inonotus obliquus) aqueous extract suppresses cancer progression and maintains body temperature in mice. Heliyon 2 (5), e00111. 10.1016/j.heliyon.2016.e00111 27441282 PMC4946216

[B152] Seo H.-K. Lee S.-C. (2010). Antioxidant activity of subcritical water extracts from Chaga mushroom (Inonotus obliquus). Sep. Sci. Technol. 45 (2), 198–203. 10.1080/01496390903423899

[B153] Shashkina M. Y. Shashkin P. Sergeev A. (2006). Chemical and medicobiological properties of chaga (review). Pharm. Chem. J. 40 (10), 560–568. 10.1007/s11094-006-0194-4

[B154] Shikov A. N. Pozharitskaya O. N. Makarov V. G. Wagner H. Verpoorte R. Heinrich M. (2014). Medicinal plants of the Russian Pharmacopoeia; their history and applications. J. Ethnopharmacol. 154 (3), 481–536. 10.1016/j.jep.2014.04.007 24742754

[B155] Shin Y. Tamai Y. Terazawa M. (2000). Chemical Constituents of Inonotus obliquus Ⅰ.: a new triterpene, 3β-hydroxy-8, 24-dien-lanosta-21, 23-lactone from sclerotium. Eurasian J. For. Res. 1, 43–50. 10.1615/IntJMedMushr.v2.i3.30

[B156] Silvat D. (2013). Biodegradation of vinasse: fungal lignolytic enzymes and their application in the bioethanol industry, I. Taylor & Francis.

[B157] Singh A. K. Bilal M. Iqbal H. M. Meyer A. S. Raj A. (2021). Bioremediation of lignin derivatives and phenolics in wastewater with lignin modifying enzymes: status, opportunities and challenges. Sci. Total Environ., 777, 145988–146021. 10.1016/j.scitotenv.2021.145988 33684751

[B158] Sivasakthi S. Usharani G. Saranraj P. (2014). Biocontrol potentiality of plant growth promoting bacteria (PGPR)-Pseudomonas fluorescens and Bacillus subtilis: a review. Afr. J. Agric. Res. 9 (16), 1265–1277. 10.5897/AJAR2013.7914

[B159] Skov J. Kania P. W. Holten-Andersen L. Fouz B. Buchmann K. (2012). Immunomodulatory effects of dietary β-1, 3-glucan from Euglena gracilis in rainbow trout (Oncorhynchus mykiss) immersion vaccinated against Yersinia ruckeri. Fish shellfish Immunol. 33 (1), 111–120. 10.1016/j.fsi.2012.04.009 22548789

[B160] Softa M. Percoco G. Lati E. Bony P. (2019). Birch Sap (&lt;i&amp;gt;Betula alba&amp;lt;/i&amp;gt;) and Chaga Mushroom (&lt;i&amp;gt;Inonotus obliquus&amp;lt;/i&amp;gt;) Extracts Show Anti-Oxidant, Anti-Inflammatory and DNA Protection/Repair Activity &lt;i&amp;gt;*in vitro*&amp;lt;/i&amp;gt;. J. Cosmet. Dermatological Sci. Appl. 9 (02), 188–205. 10.4236/jcdsa.2019.92016

[B161] Somnath R. Shibu B. Usha C. Bishwanath C. (2015). Evaluation of spent mushroom substrate as biofertilizer for growth improvement of Capsicum annuum L. J. Appl. Biol. Biotechnol. 3 (3), 22–27. 10.7324/JABB.2015.3305

[B162] Song F.-Q. Liu Y. Kong X.-S. Chang W. Song G. (2013). Progress on understanding the anticancer mechanisms of medicinal mushroom: Inonotus obliquus. Asian Pac. J. Cancer Prev. 14 (3), 1571–1578. 10.7314/apjcp.2013.14.3.1571 23679238

[B163] Song H.-S. Lee Y.-J. Kim S.-K. Moon W.-K. Kim D.-W. Kim Y.-S. (2004). Downregulatory effect of AGI-1120$({\alpha}-glucosidase inhibitor) $ and chaga mushroom (Inonotus obliquus) on cellular $ NF-{\kappa} B $ activation and their antioxidant activity. Korean J. Pharmacogn. 35 (1), 92–97.

[B214] Stamets P. (2005). Mycelium running: how mushrooms can help save the world. Berkeley: Ten speed Press.

[B164] Streeter C. Conway K. Horn G. Mader T. (1982). Nutritional evaluation of wheat straw incubated with the edible mushroom, Pleurotus ostreatus. J. Animal Sci. 54 (1), 183–188. 10.2527/jas1982.541183x

[B165] Sun J.-E. Ao Z.-H. Lu Z.-M. Xu H.-Y. Zhang X.-M. Dou W.-F. (2008). Antihyperglycemic and antilipidperoxidative effects of dry matter of culture broth of Inonotus obliquus in submerged culture on normal and alloxan-diabetes mice. J. Ethnopharmacol. 118 (1), 7–13. 10.1016/j.jep.2008.02.030 18434051

[B166] Sysoeva M. Yumaeva L. Kuznetsova O. Y. Ziyatdinova G. Budnikov G. Mel’nikova N. (2012). Study of the composition of biologically active compounds in chaga meal. Perspectives of application of chaga meal in pharmaceutical industry. Russ. J. General Chem. 82 (3), 586–594. 10.1134/s1070363212030383

[B167] Szychowski K. A. Skóra B. Pomianek T. Gmiński J. (2021). Inonotus obliquus–from folk medicine to clinical use. J. traditional complementary Med. 11 (4), 293–302. 10.1016/j.jtcme.2020.08.003 PMC8240111 34195023

[B168] Taji S. Yamada T. Wada S.-i. Tokuda H. Sakuma K. Tanaka R. (2008). Lanostane-type triterpenoids from the sclerotia of Inonotus obliquus possessing anti-tumor promoting activity. Eur. J. Med. Chem. 43 (11), 2373–2379. 10.1016/j.ejmech.2008.01.037 18387711

[B169] Tanaka R. Toyoshima M. Yamada T. (2011). New lanostane-type triterpenoids, inonotsutriols D, and E, from Inonotus obliquus. Phytochem. Lett. 4 (3), 328–332. 10.1016/j.phytol.2011.07.001

[B170] Teplyakova T. V. Pyankov O. V. Safatov A. S. Ovchinnikova A. S. Kosogova T. A. Skarnovich M. O. (2022). Water extract of the chaga medicinal mushroom, Inonotus obliquus (agaricomycetes), inhibits SARS-CoV-2 replication in Vero E6 and Vero cell culture experiments. Int. J. Med. Mushrooms 24 (2), 23–30. 10.1615/IntJMedMushrooms.2021042012 35446519

[B215] Turner N. J. Cuerrier A. (2022). ‘Frog’s umbrella’and ‘ghost’s face powder’: the cultural roles of mushrooms and other fungi for Canadian Indigenous Peoples. Botany 100 (2), 183–205. 10.1139/cjb-2021-0052

[B171] Thawthong A. Karunarathna S. C. Thongklang N. Chukeatirote E. Kakumyan P. Chamyuang S. (2014). Discovering and domesticating wild tropical cultivatable mushrooms. Chiang Mai J. Sci. 41 (4), 731–764.

[B172] Thomas P. W. Elkhateeb W. A. Daba G. M. (2020). Chaga (Inonotus obliquus): a medical marvel but a conservation dilemma. Sydowia 72, 123–130. 10.12905/0380.sydowia72-2020-0123

[B173] Tsatmali M. Ancans J. Thody A. J. (2002). Melanocyte function and its control by melanocortin peptides. J. Histochem. Cytochem. 50 (2), 125–133. 10.1177/002215540205000201 11799132

[B174] Ţura D. Wasser S. P. Zmitrovich I. V. (2018). Wood-inhabiting fungi: applied aspects. CRC Press, 245–292. Fungi.

[B216] Uzun I. (2004). Use of spent mushroom compost in sustainable fruit production. J. Fruit Ornam. Plant Res. 12, 157–165. Available at: http://www.inhort.pl/files/journal_pdf/journal_2004spec/full2004-18spec.pdf.

[B175] Van Kuijk S. Sonnenberg A. Baars J. Hendriks W. Cone J. (2015). Fungal treated lignocellulosic biomass as ruminant feed ingredient: a review. Biotechnol. Adv. 33 (1), 191–202. 10.1016/j.biotechadv.2014.10.014 25447421

[B176] Wang L.-X. Lu Z.-M. Geng Y. Zhang X.-M. Xu G.-H. Shi J.-S. (2014). Stimulated production of steroids in Inonotus obliquus by host factors from birch. J. Biosci. Bioeng. 118 (6), 728–731. 10.1016/j.jbiosc.2014.05.022 25027706

[B177] Wasser S. (2021). Book review: medicinal mushrooms: the essential guide. Int. J. Med. Mushrooms 23, 97–101. 10.1615/intjmedmushrooms.2021040287

[B178] Wasser S. P. Volz P. A. (2017). Medicinal mushrooms in human clinical studies. Part I. Anticancer, oncoimmunological, and immunomodulatory activities: a review. Int. J. Med. Mushrooms 19 (6), 279–317. 10.1615/IntJMedMushrooms.v19.i4.10 28605319

[B179] Wold C. W. Kjeldsen C. Corthay A. Rise F. Christensen B. E. Duus J. Ø. (2018). Structural characterization of bioactive heteropolysaccharides from the medicinal fungus Inonotus obliquus (Chaga). Carbohydr. Polym. 185, 27–40. 10.1016/j.carbpol.2017.12.041 29421057

[B180] Xiao S. Tian Z. Wang Y. Si L. Zhang L. Zhou D. (2018). Recent progress in the antiviral activity and mechanism study of pentacyclic triterpenoids and their derivatives. Bentham Sci. 38, 951–976. 10.1002/med.21484 PMC7168445 29350407

[B181] Xiuhong Z. Yue Z. Shuyan Y. Zhonghua Z. (2015). Effect of Inonotus obliquus polysaccharides on physical fatigue in mice. J. Traditional Chin. Med. 35 (4), 468–472. 10.1016/s0254-6272(15)30126-6 26427119

[B182] Xu X. Pang C. Yang C. Zheng Y. Xu H.-Y. Lu Z. (2010). Antihyperglycemic and antilipidperoxidative effects of polysaccharides extracted from medicinal mushroom Chaga, Inonotus obliquus (Pers.: Fr.) Pilat (Aphyllophoromycetideae) on alloxan-diabetes mice. Int. J. Med. Mushrooms 12 (3), 235–244. 10.1615/intjmedmushr.v12.i3.20

[B183] Xu X. Wu Y. Chen H. (2011). Comparative antioxidative characteristics of polysaccharide-enriched extracts from natural sclerotia and cultured mycelia in submerged fermentation of Inonotus obliquus. Food Chem. 127 (1), 74–79. 10.1016/j.foodchem.2010.12.090

[B184] Yan Z.-F. Yang Y. Tian F.-H. Mao X.-X. Li Y. Li C.-T. (2014). Inhibitory and acceleratory effects of Inonotus obliquus on tyrosinase activity and melanin formation in B16 melanoma cells. Evidence-based complementary Altern. Med. 2014, 259836. 10.1155/2014/259836 PMC4145793 25197307

[B185] Ying Y.-M. Zhang L.-Y. Zhang X. Bai H.-B. Liang D.-E. Ma L.-F. (2014). Terpenoids with alpha-glucosidase inhibitory activity from the submerged culture of Inonotus obliquus. Phytochemistry 108, 171–176. 10.1016/j.phytochem.2014.09.022 25446238

[B186] Yong T. Chen S. Liang D. Zuo D. Diao X. Deng C. (2018). Actions of Inonotus obliquus against hyperuricemia through XOD and bioactives screened by molecular modeling. Int. J. Mol. Sci. 19 (10), 3222. 10.3390/ijms19103222 30340390 PMC6214139

[B187] Youn M.-J. Kim J.-K. Park S.-Y. Kim Y. Kim S.-J. Lee J. S. (2008). Chaga mushroom (Inonotus obliquus) induces G0/G1 arrest and apoptosis in human hepatoma HepG2 cells. World J. gastroenterology WJG 14 (4), 511–517. 10.3748/wjg.14.511 PMC2681140 18203281

[B217] Yusoo S. Yutaka T. Minoru T. (2002). Triterpenoids, steroids, and a new sesquiterpen from Inonotus obliquus (Pers.: Fr.) Pilat. Int. J. Med. Mushrooms 4 (2), 8. 10.1615/IntJMedMushr.v4.i2.10

[B188] Zabel R. A. (1976). Basidiocarp development in Inonotus obliquus and its inhibition by stem treatments. For. Sci. 22 (4), 431–437. 10.1093/forestscience/22.4.431

[B189] Zadrazil F. (1997). Changes in *in vitro* digestibility of wheat straw during fungal growth and after harvest of oyster mushrooms (Pleurotus spp.) on laboratory and industrial scale. J. Appl. animal Res. 11 (1), 37–48. 10.1080/09712119.1997.9706159

[B190] Zhang B. Guo Y. Wang Z. Sun J. Cheng J. M. Liu R. (2008). Prognostic analysis of pulmonary metastases from hepatocellular carcinoma. Asian-Australasian J. Animal Sci. 21 (2), 237–243. 10.1007/s12072-008-9052-7 PMC2716849 19669310

[B191] Zhang D. M. Xu H. G. Wang L. Li Y. J. Sun P. H. Wu X. M. (2015). Betulinic acid and its derivatives as potential antitumor agents. Med. Res. Rev., 35(6), 1127–1155. 10.1002/med.21353 26032847

[B192] Zhao F. Mai Q. Ma J. Xu M. Wang X. Cui T. (2015). Triterpenoids from Inonotus obliquus and their antitumor activities. Fitoterapia 101, 34–40. 10.1016/j.fitote.2014.12.005 25542686

[B193] Zhao F. Xia G. Chen L. Zhao J. Xie Z. Qiu F. (2016). Chemical constituents from Inonotus obliquus and their antitumor activities. J. Nat. Med. 70 (4), 721–730. 10.1007/s11418-016-1002-4 27180084

[B194] Zheng W. Miao K. Liu Y. Zhao Y. Zhang M. Pan S. (2010). Chemical diversity of biologically active metabolites in the sclerotia of Inonotus obliquus and submerged culture strategies for up-regulating their production. Appl. Microbiol. Biotechnol. 87, 1237–1254. 10.1007/s00253-010-2682-4 20532760

[B195] Zheng W. Zhang M. Zhao Y. Miao K. Pan S. Cao F. (2011a). Analysis of antioxidant metabolites by solvent extraction from sclerotia of Inonotus obliquus (Chaga). Phytochem. Anal. 22 (2), 95–102. 10.1002/pca.1225 21259372

[B196] Zheng W. Zhao Y. Zhang M. Wei Z. Miao K. Sun W. (2009). Oxidative stress response of Inonotus obliquus induced by hydrogen peroxide. Med. Mycol. 47 (8), 814–823. 10.3109/13693780802653933 19184774

[B197] Zheng W. Zhao Y. Zheng X. Liu Y. Pan S. Dai Y. (2011b). Production of antioxidant and antitumor metabolites by submerged cultures of Inonotus obliquus cocultured with Phellinus punctatus. Appl. Microbiol. Biotechnol. 89 (1), 157–167. 10.1007/s00253-010-2846-2 20830471

[B198] Zheng W.-F. Zhao Y.-X. Zhang M. Yin Z. Chen C. Wei Z. (2008). Phenolic compounds from Inonotus obliquus and their immune stimulating effects. Mycosystema 27 (4), 574–581.

[B199] Zhong X.-h. Ren K. Lu S.-j. Yang S.-y. Sun D.-z. (2009). Progress of research on Inonotus obliquus. Chin. J. Integr. Med. 15 (2), 156–160. 10.1007/s11655-009-0156-2 19407959

[B200] Zhou L.-W. Wang X.-Y. (2015). <I&gt;Inonotus griseus&lt;/I&gt; sp. nov. from eastern China. Mycotaxon 130 (3), 661–669. 10.5248/130.661

[B201] Zhu F. Du B. Bian Z. Xu B. (2015). Beta-glucans from edible and medicinal mushrooms: characteristics, physicochemical and biological activities. J. Food Compos. Analysis 41, 165–173. 10.1016/j.jfca.2015.01.019

[B202] Zhu F. Du B. Xu B. (2016). A critical review on production and industrial applications of beta-glucans. Food Hydrocoll. 52, 275–288. 10.1016/j.foodhyd.2015.07.003

[B203] Zmitrovich I. V. Denisova N. P. Balandaykin M. E. Belova N. V. Bondartseva M. A. Perevedentseva L. G. (2020). Chaga and its bioactive complexes: history and perspectives. Pharm. Formulas 2 (2), 84–93. 10.17816/phf34803

[B204] Zyryanova O. A. Terazawa M. Koike T. Zyryanov V. I. (2010). White birch trees as resource species of Russia: their distribution, ecophysiological features, multiple utilizations. Eurasian J. For. Res. 13 (1), 25–40.

[B209] Glamočlija J. Ćirić A. Nikolić M. Fernandes Â. Barros L. Calhelha R. C. (2015). Chemical characterization and biological activity of Chaga (Inonotus obliquus), a medicinal “mushroom”. J. Ethnopharmacol. 162, 323–332. 10.1016/j.jep.2014.12.069 25576897

